# Structure-guided selection of puromycin *N*-acetyltransferase mutants with enhanced selection stringency for deriving mammalian cell lines expressing recombinant proteins

**DOI:** 10.1038/s41598-021-84551-9

**Published:** 2021-03-04

**Authors:** Alessandro T. Caputo, Oliver M. Eder, Hana Bereznakova, Heleen Pothuis, Albert Ardevol, Janet Newman, Stewart Nuttall, Thomas S. Peat, Timothy E. Adams

**Affiliations:** 1 Biomedical Manufacturing, Commonwealth Scientific and Industrial Research Organisation, 343 Royal Parade, Parkville, Victoria 3052 Australia; 2 CSIRO Synthetic Biology Future Science Platform, GPO Box 1700, Canberra, ACT 2601 Australia

**Keywords:** Enzymes, Biochemistry, Structural biology, X-ray crystallography, Biologics, Expression systems, Protein design

## Abstract

Puromycin and the *Streptomyces alboniger*-derived puromycin *N*-acetyltransferase (PAC) enzyme form a commonly used system for selecting stably transfected cultured cells. The crystal structure of PAC has been solved using X-ray crystallography, revealing it to be a member of the GCN5-related *N*-acetyltransferase (GNAT) family of acetyltransferases. Based on structures in complex with acetyl-CoA or the reaction products CoA and acetylated puromycin, four classes of mutations in and around the catalytic site were designed and tested for activity. Single-residue mutations were identified that displayed a range of enzymatic activities, from complete ablation to enhanced activity relative to wild-type (WT) PAC. Cell pools of stably transfected HEK293 cells derived using two PAC mutants with attenuated activity, Y30F and A142D, were found to secrete up to three-fold higher levels of a soluble, recombinant target protein than corresponding pools derived with the WT enzyme. A third mutant, Y171F, appeared to stabilise the intracellular turnover of PAC, resulting in an apparent loss of selection stringency. Our results indicate that the structure-guided manipulation of PAC function can be utilised to enhance selection stringency for the derivation of mammalian cell lines secreting elevated levels of recombinant proteins.

## Introduction

The emergence of therapeutic proteins produced in mammalian cells as a major drug class has been facilitated by the adaptation of drug selection strategies to enable the derivation of stably transfected cell lines synthesising and secreting high levels of recombinant proteins. While a variety of drug-enzyme combinations have been developed for the selection of cells stably incorporating exogenous DNA, two approaches have dominated the biopharmaceutical landscape^1^. Dihydrofolate reductase (DHFR)-driven cell rescue and gene amplification involves, first, the transfection of a DHFR-deficient cell line e.g. the Chinese hamster ovary (CHO) cell line, CHO-DG44, followed by selection in medium lacking hypoxanthine and thymidine; subsequent culture in the presence of successive rounds of increased concentrations of methotrexate (MTX) results in gene amplification and an accompanying increase in productivity from linked transgenes. While effective, gene amplification is time-consuming and a significant number of clonal lines recovered exhibit genomic instability in the absence of selection pressure^2^. Glutamine synthetase (GS)-mediated cell line selection can be performed following transfection of cells expressing endogenous GS by inhibiting the endogenous enzyme with methionine sulfoximine (MSX)^3^. For cells naturally deficient in GS activity, for example NSO myeloma cells^4^, or those where inactivation of the GS locus has been performed, selection for stable transfectants can be achieved by growth in glutamine-deficient medium^5^. Like DHFR, GS is an amplifiable marker^6^; that said, there is a question as to whether increasing selection stringency via increasing MSX concentration contributes to increased productivity via gene amplification^5,7^. A key (and ongoing) feature of the original GS selection system was the use of the weak, simian virus 40 (SV40) late promoter element to drive transcription of the GS transgene^1^. This in itself provides a high degree of selection stringency, requiring the integration of one or more copies of the DNA vector into a transcriptionally active chromosomal locus in order to effect cell survival in the presence of MSX. This concept has been validated in a study showing GS selection performed with transcriptionally compromised promoter elements was associated with an overall increase in the productivity of recombinant monoclonal antibodies in stably transfected GS-knockout CHO cells^8^.

Puromycin, an aminonucleoside antibiotic, is a potent inhibitor of protein translation in both eukaryotic and prokaryotic organisms. The gene encoding resistance to puromycin, puromycin *N*-acetyltransferase (*pac*), was isolated from *Streptomyces alboniger*^9^ and first used as a dominant selection marker for the isolation of stably transfected mammalian cells lines in 1988^10^. The speed with which selection can be achieved (several days), the low cost of puromycin, and the compact size of the *pac* gene has seen the widespread utilisation of this versatile selection system in basic and applied biology. Despite this, very little is known about the PAC protein in the context of how structure relates to functional activity, and to our knowledge there are no reports describing manipulations of PAC activity. Nor are we aware of attempts to preference the recovery of stably transfected cell lines expressing high levels of recombinant proteins in a manner to that achieved using, for example, the GS selection platform. To address this, we have solved the first crystal structures of PAC, and have used structure-guided mutagenesis to identify mutations to enhance the recovery of stably transfected cell lines secreting elevated levels of a co-expressed recombinant protein.

## Results

### Crystal structure of recombinant PAC

Despite the extensive use of PAC as a selection marker, little effort has been made to further characterise this enzyme. Expression of recombinant PAC in *Escherichia coli* (*E. coli*) was easily achieved; purification was carried out in an automated two-step fashion utilising an N-terminal hexa-histidine tag (Supplementary Fig. S1a,b). In the absence of cofactor or substrate, PAC was highly unstable and readily precipitated. Addition of either acetyl-CoA (AcCoA) or puromycin to PAC in solution greatly improved its thermal stability, with a 12 °C increase in melting temperature (Supplementary Table S3). The addition of AcCoA enabled crystallisation of PAC and the determination of its three-dimensional structure using X-ray crystallography. The limited size of the crystals, (Supplementary Fig. S1c) and the loss of diffraction upon addition of heavy atoms complicated phasing of crystallographic data. Fortunately, the combination of molecular replacement and Rosetta^11,12^ using the closest structural homologue (PDB ID: 2QEC, 29% amino acid identity) allowed the initial structure to be solved with AcCoA bound (Supplementary Table S1). This structure was used to solve a second, higher-resolution data set which had been collected from crystals grown from protein with both AcCoA and puromycin added.

**Figure 1:**
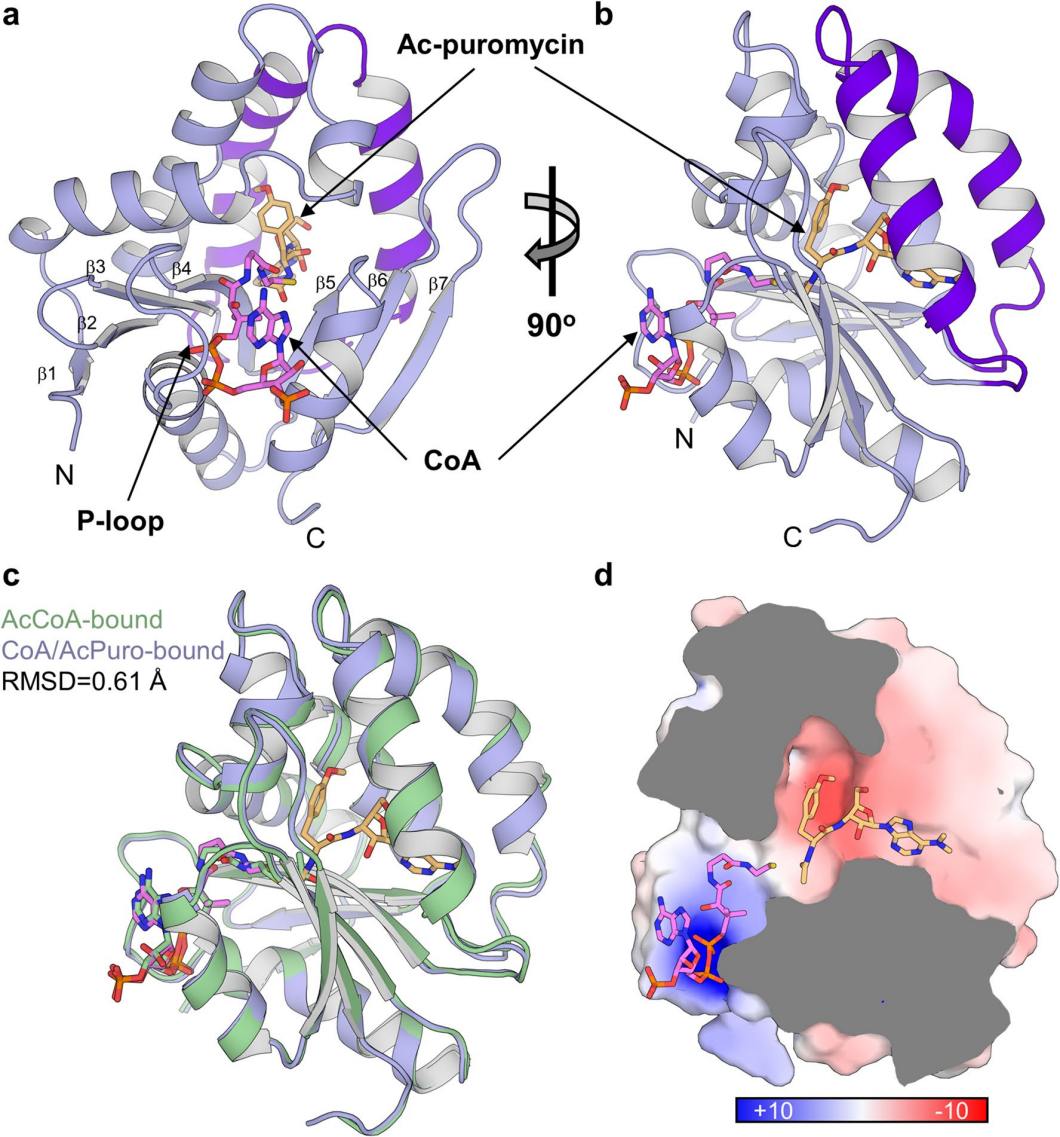
Crystal structures of PAC. (**a**, **b**) Two orientations of the structure of PAC in complex with the reaction products CoA (pink) and acetylated-puromycin (light orange). The seven β-strands that make up the central sheet of the fold are numbered. The two-helix hairpin insertion unique to PAC is highlighted in dark purple. (**c**) Overlay of the two structures presented in this work with the same representation as (**b**). The green structure represents PAC with AcCoA bound and shows the little deviation in global structure. (**d**) Electrostatic potential mapping onto the surface of PAC calculated using Adaptive Poisson-Boltzmann Solver Tool and pdb2pqr in PyMOL^48,49^. The depiction is of a cross-section of the active site in the same orientation as shown in (**b**, **c**) and the surface is represented and a scale between − 10 and + 10 kT/e.

PAC adopts a fold consistent with the GCN5-related *N*-acetyltransferase (GNAT) family (Fig. 1a-b and Supplementary Fig. S2), consisting of a seven-stranded β-sheet interspersed with four α-helices^13^. PAC displays the typical structural hallmarks of this family which includes a cleft in the middle of the sheet between the parallel strands 4 and 5 that facilitates AcCoA binding. AcCoA binding is primarily through a “P-loop” formed by a conserved Q–X–X–G–X–G motif in the loop after β-strand 4, which allows the coordination of the pyrophosphate moiety of the CoA by the protein backbone. The poor initial electron density around the adenine moiety of the CoA made its unambiguous placement difficult and the adenine moiety has been modelled in both orientations (Supplementary Fig. S3). In the two crystal structures obtained in this work, with either AcCoA bound, or the trapped reaction products (CoA and acetylated-puromycin) in the other, the absence of puromycin causes little change to the global structure of PAC (Fig. 1c).

The entry point of the acetyl acceptor substrate puromycin is proximal to the AcCoA-binding region, and this is also where most structural differences can be observed relative to other GNAT enzymes. The most distinctive feature is the insertion of an α-helical hairpin between β-strands 3 and 4 (Fig. 1b dark-purple helices); this forms the entry point for puromycin into the active site. The electrostatic potential of the lining of the active site is polarised: it has a predominantly negative potential for the puromycin-binding site and the opposed positive potential for the AcCoA-binding site (Fig. 1d).

### Characterisation of amino acids residues that impact PAC enzyme function

GNAT enzymes catalyse *N*-acetylation by enabling the acceptor to carry out a nucleophilic attack on the AcCoA thioester^14^. The amino group undergoing the group transfer reaction must be uncharged in order to be an effective nucleophile, but the pK_a_ of the primary amine in puromycin is around 7.2^15^, negating the need for a general base typically seen in other GNAT enzymes. A general acid is required for protonation of the nascent thiol, which is predominantly a tyrosine in other GNAT proteins^16^.

**Figure 2:**
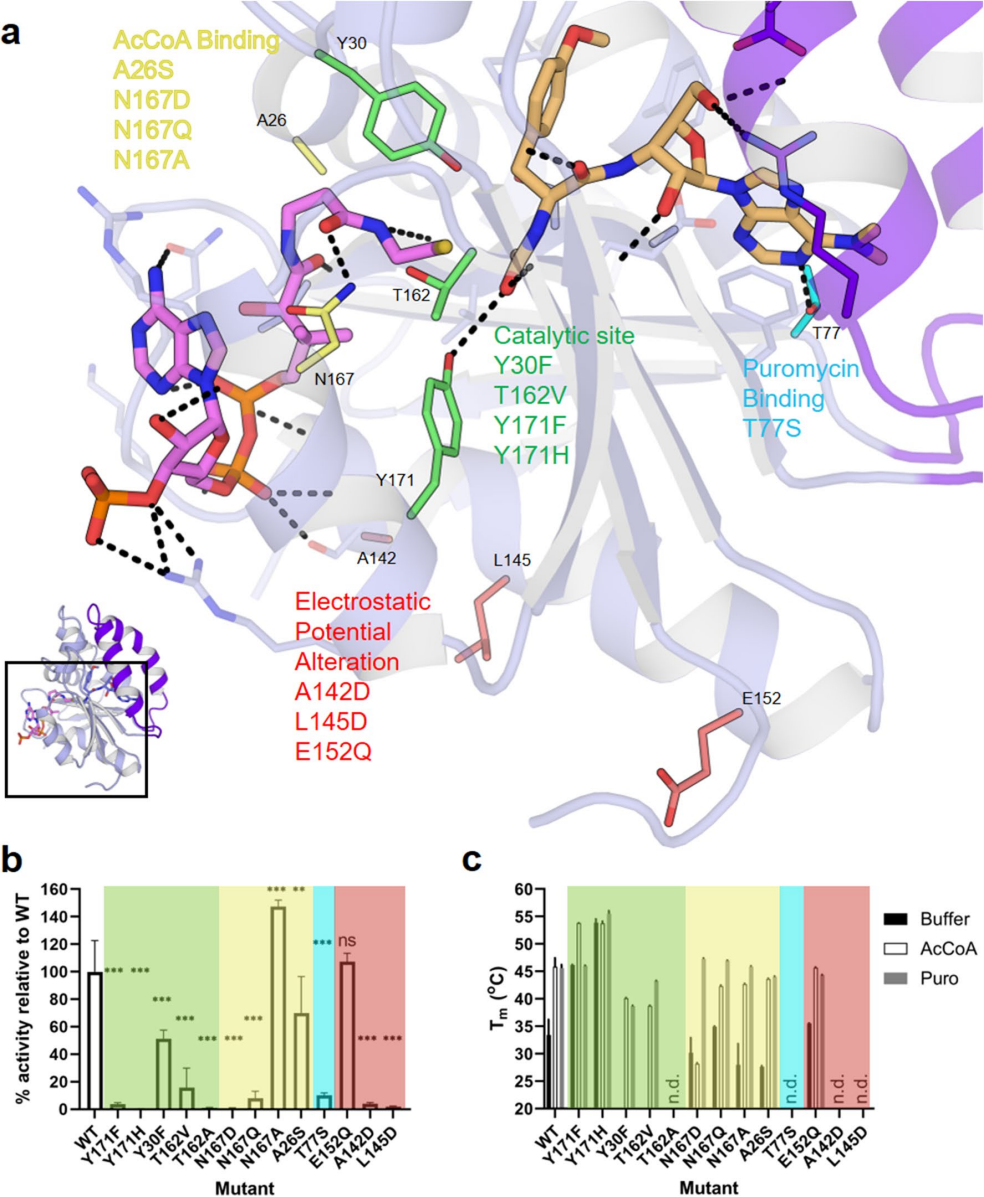
Structure-based enzyme engineering of PAC. (**a**) A network of molecular interactions between the PAC sidechains and (Ac)CoA (pink) and (acetylated-)puromycin (orange) identified for mutagenesis based on their role in substrate binding and catalysis. (**b**) Relative enzyme activities of PAC mutants under steady-state conditions normalised to protein concentration. Numerical values can be found in Supplemental Table S2. Comparisons in activity measurements are using a one-way ANOVA analysis: ***p < 0.001, **p < 0.01. “ns” not significant. (**c**) Differential scanning fluorimetry derived melting temperatures (T_m_) of PAC mutants in purification buffer with or without the addition of either 0.2 mM AcCoA or 0.2 mM puromycin. Numerical values can be found in Supplementary Table S3. Error bars indicate standard deviations from at least three repetitions. “n.d.” indicates melting curves that showed no measurable transition.

Based on the crystal structures, four classes of mutations were designed to reduce the acetylation activity of PAC (visually summarised in Fig. 2a). The catalytic site class (in green) aims to substitute the residues that are directly involved in the reaction mechanism. The goal of the acetyl donor and acceptor binding residue mutation classes (in yellow and in blue, respectively) was to directly affect hydrogen bonds by perturbing the interactions to either substrate. The fourth class (in red) was designed to destabilize the transition state by perturbing the electrostatic potential in the active site with mutations of second shell residues.

The final class of mutations uses a strategy is based on the assumption that the electrostatic organization of the enzyme residues contribute to accelerate the chemical reaction by stabilizing the transition state with a local electric field (LEF)^17,18^. The low dielectric constant of the protein and the asymmetrically scattered charged residues results in an intense LEF that can be particularly important in reactions involving charge transfer. Rational protein engineering efforts have aimed at modifying the LEF to enhance the enzymatic reactivity in enzymes^19^. In contrast, we designed a series of mutants with the intention to decrease the intensity of the LEF in the active site, hence reducing the enzymatic activity of PAC.

An assay measuring relative enzymatic activity was used to measure the effect of the designed point mutations following purification of mutant, recombinant enzyme (Supplementary Fig. S4). PAC activity was measured with real-time detection of the CoA product with Ellman’s reagent (DNTB) after the acetylation of puromycin. The initial velocities under steady-state conditions of the mutants were normalized to the wild-type by protein concentration. This allowed a relative comparison of the effect of the point mutants on activity (Fig. 2b and Supplementary Table S2). All point mutants, with the exception of N167A and E152Q, resulted in a statistically significant reduction of activity relative to the WT enzyme. Most of the point mutants showed a decrease in enzyme activity, likely due to the direct role these residues play in catalysis or in substrate binding. The increase in activity of the N167A mutation is surprising as it was expected that this mutation would lead to the abolition of key interactions with the CoA carbonyl oxygen proximal to thiol as well as to the adenine moiety. The E152Q mutant was the solitary mutant with a null effect on activity.

A number of mutants could not be expressed, possibly as a result of deleterious effects of the mutations on folding. The thermal stability of the expressed PAC mutants was characterised by differential scanning fluorimetry (DSF), where the melting temperatures (T_m_) were assessed in three different conditions: in the buffer used during purification and enzyme assays; supplemented with 0.2 mM AcCoA; or supplemented with 0.2 mM puromycin (Fig. 2c and Supplementary Table S3). Several mutants with negligible activity did not have measurable unfolding transitions, indicating poor folding/stability. In these cases, where both stability and activity were reduced, an overall detrimental effect on folding cannot be fully ruled out and would require more detailed examination of their folding status. There was generally little correlation between the melting temperatures and *N*-acetyltransferase activity. The addition of either binding partner generally increased the thermal stability of the mutant protein. There was little difference in T_m_ between AcCoA and puromycin for the wild-type enzyme but a marked increase (> 10 °C) compared to the absence of either substrate. In the class of mutations that were designed to alter AcCoA binding, the difference in T_m_ in the presence of puromycin had little effect. However, some mutants had a more pronounced decrease in T_m_ in the presence of AcCoA compared to the WT, indicating a disruption to AcCoA binding. The sole puromycin-binding mutant, T77S, showed both poor activity and folding defects that precluded T_m_ determination suggesting a crucial role in puromycin binding.

### Selection of stably transfected mammalian cell pools secreting higher levels of a soluble, recombinant protein using mutant PAC

**Figure 3:**
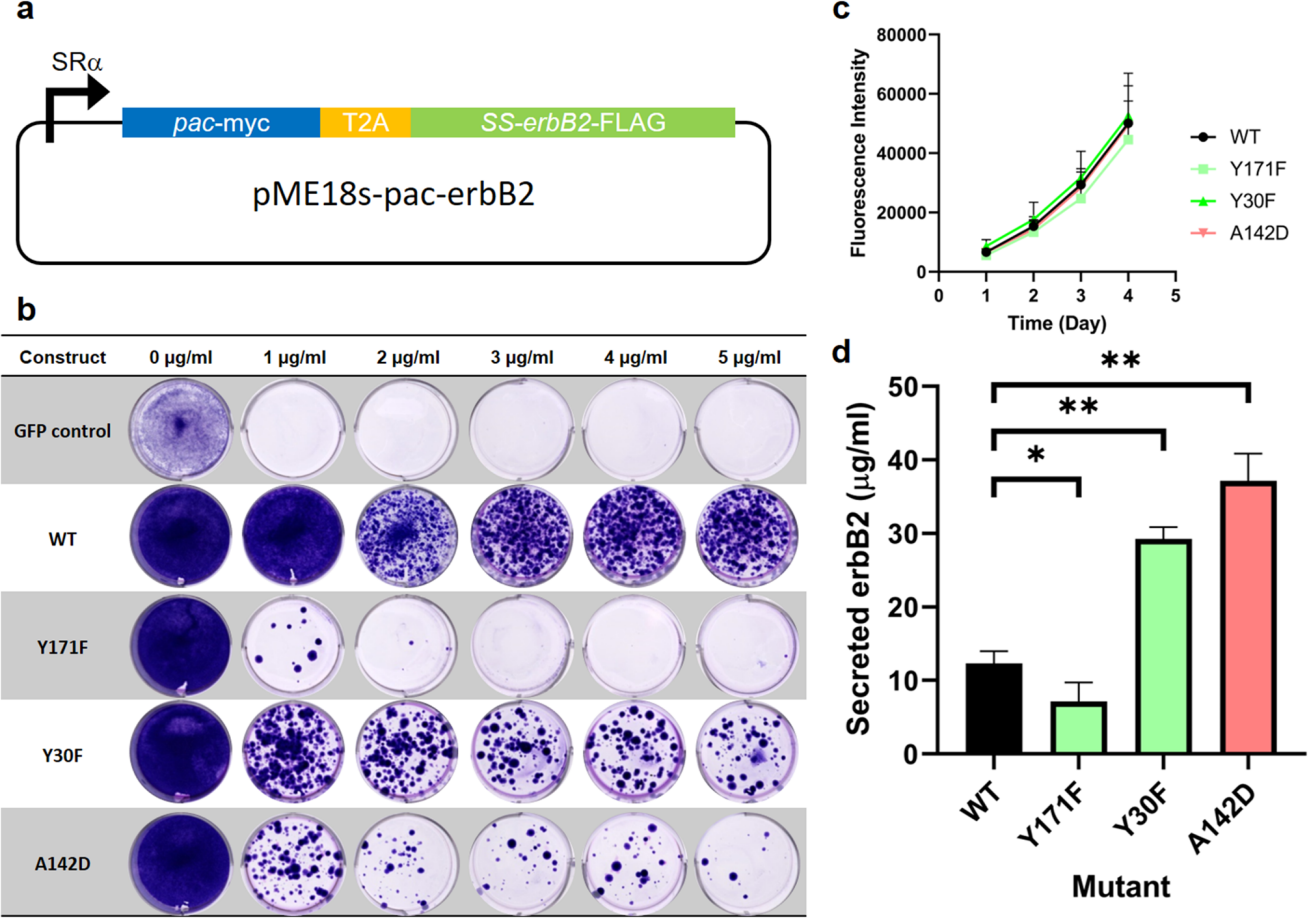
Assessment of the effects of select PAC mutations on puromycin cell selection and recombinant protein production in stably integrated cell pools. (**a**) Schematic of the vector used in this study. (**b**) Colony formation with increasing puromycin concentrations of three selected PAC mutants relative to the wild-type PAC in HEK293-F cells. (**c**) Effect of PAC mutations on the cell growth kinetics assessed by alamarBlue assay over the time period used for assessing ErbB2 production. (**d**) Quantitation of secreted ErbB2 from stable cell pools of different PAC mutants after 3 days using a sandwich ELISA. Colour coding is consistent with the classes of mutations described in Fig. 2. Error bars represent standard deviations from four biological repeats each measured in triplicate for (**c**, **d**) and comparisons are using a one-way ANOVA analysis: *0.034 and **0.0001.

The 13 PAC mutants were subcloned into the pME18s:*pac-T2A-erbB2* mammalian expression vector (Fig. 3a). This vector drives the transcription of a single mRNA that encodes both PAC and the soluble ectodomain of the human ErbB2 cell-surface tyrosine kinase^20^, linked in-frame with the T2A peptide derived from the foot-and-mouth disease virus^21^. The secreted ErbB2 ectodomain comprises a 623 amino acid protein that includes two cysteine-rich domains and seven *N*-linked glycans. The panel of mutants was then assessed for the ability to promote colony formation following transfection of adherent cultures of 293-F cells and subsequent drug selection employing a range of puromycin concentrations (0–5.0 μg/mL) (Fig. 3b and Supplementary Table S4). A clear trend was established; PAC mutants with low levels of enzyme activity such as Y171F generated very few colonies at low (1 μg/mL) puromycin concentrations and none at higher concentrations (3–5 μg/mL; Fig. 3b), while PAC mutants with higher levels of enzyme activity such as Y30F, retained their ability to promote efficient colony formation across all puromycin concentrations used, albeit with fewer colonies than the WT (Fig. 3b and Supplementary Table S4).

Stably transfected cell pools derived with WT, Y171F, Y30F and A142D *pac-T2A-erbB2* vector constructs and selection with 2 μg/mL puromycin were expanded for further analysis. While there was a clear correlation between mutation-induced reduction in enzyme activity and colony formation under drug selection (Fig. 3b), there was no significant difference in the rate of cellular proliferation between the four lines when using established cultures (Fig. 3c). To quantitate the levels of secreted ErbB2, supernatants were harvested after equal numbers of cells were plated and assayed using an ELISA employing the anti-ErbB2 monoclonal antibody, pertuzumab, as a capture antibody and anti-FLAG:HRP as the reporter antibody. Polyclonal transfectants generated with Y30F and A142D PAC mutants secreted between 2-to-3-fold more ErbB2 than WT cells (Fig. 3c). In contrast, cell pools derived using the Y171F mutant produced approximately half the level of ErbB2 as WT cell cultures.

**Figure 4:**
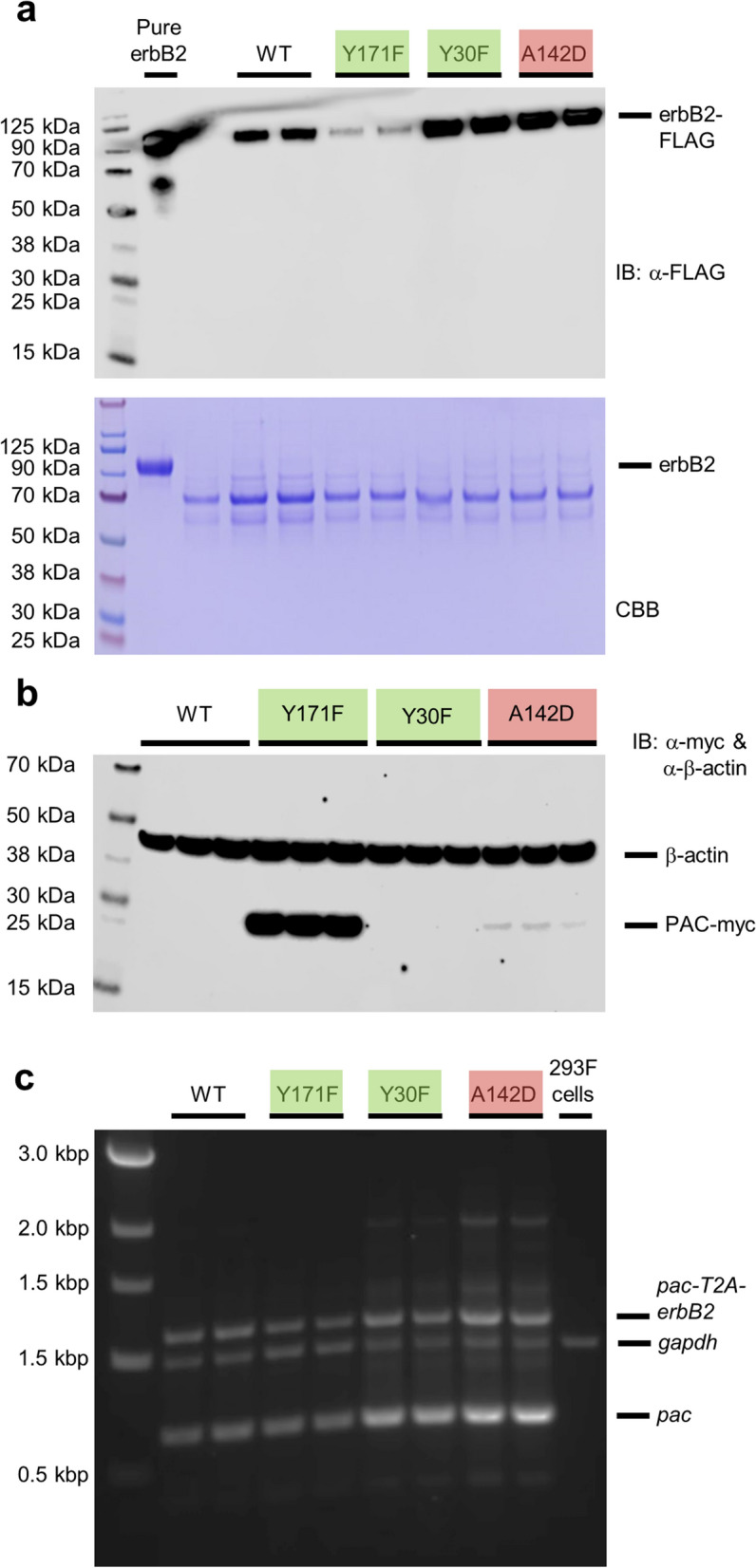
Assessment of stably transfected HEK293-F pools derived with the wild-type and mutant pac-T2A-erbB2 cassettes at the protein and RNA levels. (**a**) Verification of ErbB2 molecular weight by immunoblotting and Coomassie Brilliant Blue stained gels of culture supernatants used for ELISA studies. (**b**) Intracellular expression of myc-tagged PAC relative to the β-actin loading control. (**c**) A multiplexed reverse transcriptase PCR of the *pac-T2A-erbB2* transcript from isolated cellular RNA. Replicates are of individual wells grown and processed in parallel and are carried out in triplicate in (**a**, **b**) and duplicate in (**c**). Colour coding is consistent with the classes of mutations described in Fig. 2.

To validate the integrity of secreted ErbB2 ectodomain, western blot analysis was performed on culture supernatants from transfected cells using an anti-FLAG monoclonal antibody specific for the C-terminal FLAG tag. A single band with an approximate molecular weight of 90 kDa (Fig. 4a) was observed in supernatants from all transfected cultures, with the relative intensity of signal between cultures generated with the different PAC isoforms consistent with the quantitative ELISA data. To see if this was reflected in the levels of intracellular PAC, cell lysates were prepared, and the presence of myc-tagged PAC established by western blotting. No signal was observed in lysates from WT and Y30F cell pools, while a weak signal was detected in lysates from A142D cells (Fig. 4b); in contrast, the level of Y171F PAC was dramatically elevated in comparison. RT-PCR of total RNA isolated from all transfectants revealed the presence of *pac* transcripts, with similar signals generated from the WT and Y171F RNA templates, while elevated signals were associated with Y30F and A142D RNA (Fig. 4c).

## Discussion

A variety of strategies have been employed to bias the recovery of stably transfected mammalian cell lines secreting high levels of therapeutic proteins. While both gene amplification and increased stringency of selection through the use of weakened transcription of the selectable marker remain standard approaches, elevated productivity has also been achieved by targeting the enzyme driving selection, either through engineered destabilisation of both the mRNA encoding the enzyme and the enzyme itself^22^ or the introduction of mutations that result in attenuated enzyme activity^5^. Our structural characterisation of PAC has allowed the identification of mutations that functionally impair the enzyme and can be used to enhance selection stringency in mammalian cell lines that is accompanied by increased productivity.

While puromycin has been widely used to enhance our understanding of protein synthesis, as well as a versatile tool in a variety of biological applications^23^, surprisingly little is known about the PAC enzyme. Acetylation is a common strategy of microbes to inactivate antibiotics^24^. The function of PAC is thus an unsurprising addition to the list of other enzymes made by microbes that have evolved to counter the chemical warfare waged between microbes. In this case, puromycin is the chemical agent that *Streptomyces alboniger* makes to kill competing microbes along with the enzyme PAC to prevent its own self-destruction. Many members of the GNAT family are enzymes that inactivate antibiotics including the aminoglycosides streptomycin and kanamycin^25^.

The structural characterisation of many GNAT enzymes has resulted in general conclusions of their catalytic properties. The most obvious difference to other acetyltransferases is the absence of the catalytic cysteine-histidine-aspartate triad found commonly in other *N*-acetyltransferases^26^. PAC does not have a cysteine in the catalytic site that would otherwise be needed to create an acetylated enzyme intermediate using a ping-pong mechanism. Rather, it is likely PAC facilitates the direct acetyl group transfer through the geometric placement of the puromycin amino group to carry out a nucleophilic attack on the acetyl group of the donor. The order of substrate binding has not been determined but across the GNAT family there does not appear to be a clear trend to ordered or random, sequential binding of AcCoA and the acceptor substrate. Based on our crystal structure, the general acid required to protonate the nascent CoA thiol is very likely to be Y171, which is consistent with other GNAT enzymes by its proximity to the CoA thiol. The removal of this residue greatly reduces activity, but the aqueous environment probably makes up for the lack of acid for protonation of the CoA thiol as observed in some GNAT enzymes^27^.

The absence of a general base is more difficult to explain without a more detailed enzymological analysis of the reaction mechanism. The neutral pK_a_ of puromycin makes it unlikely that it would require a general base to abstract a proton to allow the nucleophilic attack to occur. However, there is a precedent in related GNAT enzymes to have a distal general base that “conducts” the proton through a series of water molecules^28,29^. A candidate for this could be E161, but its direct engagement in puromycin binding would make it difficult to dissect which of these roles would be affected by mutation. Similar to PAC, a yeast *N*-acetyltransferase substrate, glucosamine-6-phosphate, has a pK_a_ of 7.75 and appears to lack a general base typically used to increase the nucleophilicity of the attacking group^30^.

Of the three mutants that were designed to reduce the activity of PAC by perturbing the intensity of the local electric field in the wild type, two of them (A142D and L145D) showed a decrease in acetylation activity. Because these residues are far from the active site, we assume that they are not directly related to the catalytic mechanism. Despite this, at least one of these mutants (A142D) showed the ability to confer cell survivability and increased production of ErbB2 from stable cell pools.

The utilisation of mutant forms of GS with attenuated enzyme activity to derive antibody-secreting CHO-K1 cell lines with enhanced stability and higher productivity than cell lines derived with WT GS has recently been reported^31^. The first mutation analysed—R324C—was identified as a congenital mutation with less than 5% of WT enzyme activity, involved in ATP binding by GS^32^. A panel of mutations with reduced activity, identified by alanine-scanning mutagenesis of the conserved, substrate-binding domain of GS for ATP, glutamate and ammonia, was subsequently identified. In both stably transfected cell pools and clonally derived cell lines, there was an inverse correlation between enzyme activity and productivity. In our study, which was limited to stably transfected cell pools, two of the three selected PAC mutants—Y30F (51.1% of WT activity) and A142D (4%)—resulted in increased productivity of secreted ErbB2 in the range of two-to-three-fold over that obtained in WT cultures, with no impairment to cell growth at a puromycin concentration of 2 μg/mL (Fig. 3c). The elevated level of productivity was associated with an increase in the level of transcripts for the mutant *pac*-*T2A*-*erbB2* mRNA cassette relative to WT. The level of secreted ErbB2 in culture supernatants derived from the Y30F and A142D 293-F transfectants (30–35 μg/mL) after 72 h in monolayer culture was higher than we have previously obtained using optimised conditions for the culture of suspension-adapted cultures of stably transfected 293EBNA cells expressing ErbB2 from the episomal expression vector, pApex-3P^33^, where we obtained 27 μg/mL after 7 days in culture (unpublished observations).

In contrast, the 293-F cell pool generated with Y171F (3.6%) was characterised by a lower level of ErbB2 productivity (Fig. 3d). This appears to be the result of an increase in stability of PAC protein: while expression of WT and mutant PAC in cell lysates from stably transfected cell pools was below the level of detection achievable using western blotting, Y171F is easily detected. We have found that following transient transfection of HEK293T cells, we can detect WT, A142D and Y171F protein in cell lysates, but not Y30F (Supplementary Fig. S5). RT-PCR revealed that the level of transcript encoding Y171F was similar to that encoding WT. It would therefore appear that the potential increase in selection stringency as a result of mutagenesis is offset by the significantly elevated levels of Y171F protein.

The structure-guided identification of mutants that result in attenuated PAC enzyme activity extend the flexibility of the puromycin-PAC drug selection platform to be configured to favour the recovery of stably transfected cells lines producing elevated levels of target proteins. It is anticipated that this refinement may encourage the increased utilisation of this approach for the production of biopharmaceutical proteins of clinical interest.

## Methods

### Bacterial and mammalian *pac* expression vectors

The native full-length *pac* gene was amplified from the retroviral vector, pBABE-puro^34^, using PrimeSTAR HS DNA polymerase (Takara Bio) according to the manufacturer’s protocol and primers detailed in Supplementary Table S5 and subcloned in-frame into pET43a bacterial expression vector incorporating a N-terminal His_6_-tag.

Mutagenesis was carried out using the Q5 Site-directed mutagenesis kit (New England Biolabs) following the manufacturers protocol. All primers and annealing temperatures used for the PCR can be found in Supplementary Table S6.

For expression in mammalian cell lines, a DNA cassette comprising the coding region for PAC (carrying a C-terminal c-Myc tag) fused in-frame with a furin cleavage site, the foot-and-mouth disease virus T2A peptide^21^ and the signal peptide and N-terminal 623 amino acids of the ectodomain of human ErbB2, terminating in a C-terminal FLAG tag, was assembled and subcloned into the mammalian expression vector, pME18S^35^. This was achieved by amplifying both the vector and the inserts (primers in Supplementary Table S5) and assembling using NEBuilder HiFi DNA Assembly Kit (New England Biolabs) according to the manufacturer's protocol. All constructs were verified using Sanger Sequencing by Micromon Genomics (Monash University, Clayton, Australia).

### Expression and purification of recombinant PAC isoforms

Bacterial expression of wild-type and mutant PAC was performed in *E. coli* BL21 (DE3). Bacterial transformants were inoculated into 1 L Terrific Broth containing 100 μg/mL carbenicillin (GoldBio) and grown at 37 °C at 190 rpm in 2 L baffled flasks. Upon reaching an OD_600_ of 1, the temperature was reduced to 18 °C and cultures induced with 0.5 mM isopropyl-β-d-thiogalactoside (GoldBio). The following day, the cells were harvested by centrifugation at 5000*g*.

The cells were resuspended in lysis buffer (50 mM Tris–HCl, pH 8.0, 300 mM NaCl, 10% w/v glycerol, 2 mM MgCl_2_, 5 mM imidazole) containing manufacturer’s recommended amounts of cOmplete EDTA-free Protease Inhibitor Cocktail (Roche) and Benzonase (Merck-Millipore). Homogenisation was carried out in either an EmulsiFlex C3 or C5 (Avestin) with at least two passages at > 15,000 psi, and the lysate cleared from debris by centrifugation at 48,000*g* at 4 °C for 20 min.

The PAC proteins were purified using an automated two-step method on an ÄKTAxpress (GE Healthcare) using a 1 mL HiTrap Talon crude column (GE Healthcare) followed by injection of the peak eluate onto a HiLoad Superdex 75 16/600 prep grade column (GE Healthcare). The immobilised metal affinity chromatography (IMAC) column was equilibrated and after loading, samples washed with IMAC Buffer A (phosphate buffered saline, supplemented with an extra 150 mM NaCl and 10% glycerol). The protein was eluted with IMAC Buffer A supplemented with 200 mM imidazole. The Superdex column was run in PAC Sample Buffer (20 mM Tris–HCl, pH 8.0, 500 mM NaCl, 5% w/v glycerol). Appropriate fractions were pooled and concentrated using 10 kDa MWCO Amicon Ultra-15 (Merck Millipore) ultrafiltration units.

### Crystallisation, crystallography and structure determination

PAC concentrated to 7 mg/mL supplemented with 0.1 mM AcCoA (Cayman Chemical) was crystallised by vapour diffusion in a 150 nL:150 nL (protein:reservoir) drops with 2.5 M ammonium sulfate and 10% v/v DL-malate-MES-Tris pH 9.0. Crystals of approximately 20 μm were transferred to a drop of 3.4 M sodium malonate pH 8.5, and flash cooled in liquid nitrogen. Crystals were also grown with protein at 5 mg/mL in the presence of 2 mM AcCoA and 2 mM puromycin (Thermo Fisher Scientific) in 9.9% (w/v) 1,6-hexanediol, 1.59 M ammonium sulfate, 7.4% (v/v) polyethylene glycol 400, 0.1 M HEPES pH 8.26, and 0.5 n-hexyl-β-d-glucopyranoside. All diffraction experiments were carried out at the MX2 beamline at Australian Synchrotron (Clayton, Australia).

Diffraction images of the AcCoA only dataset were processed in autoPROC which utilises XDS and CCP4 programs^36–40^. Molecular replacement was carried out with MR-Rosetta through the Phenix user interface^11,12^. Briefly, this involved a template search using the HHPred server which identified the PDB model 2QEC as the closest structural homologue^41^. The HHPred alignment along with the template pdb file were input into the pipeline which used Phaser to identify several potential molecular replacement solutions followed by Rosetta to carry out homology modelling. The model with the most favourable Phaser and Rosetta scores was refined using autoBUSTER utilising automatically generated non-crystallographic symmetry restraints, target secondary structure restraints from the template model, and TLS. Subsequent model building after refinement was carried out in Coot^42–44^. The AcCoA/puromycin dataset was processed using xia2^39^ and the DIALS^45^ pipeline followed by molecular replacement with Phaser^45^. The ligands modelled into this structure were CoA and *N*-acetylated puromycin, the chemical library for the latter was generated with eLBOW tool from the Phenix suite^11^.

### Differential scanning fluorimetry

A 2× detection mix containing SYPRO Orange Protein stain (Sigma-Aldrich) diluted into PAC Sample Buffer to a 7.5× concentration and 0.4 mM AcCoA or 0.4 mM puromycin was added as required. 10 µL of PAC at 0.1 mg/mL and 10 μL of the 2× detection mix were combined in a 96 well white PCR plate (Thermo Fisher Scientific). Fluorescence with excitation/emission filters at 490/570 nm was measured while ramping the temperature 1.0 °C every minute from 25 to 100 °C. Melting temperatures (T_m_) were determined using Meltdown^46^.

### PAC enzyme assay

Acetylation of puromycin^47^ was adapted to be measured in a 96-well plate (Axygen Scientific) by preparing a 1:1 mixture of diluted PAC with a master mix containing 0.4 mM AcCoA, 0.4 mM puromycin, 0.4 mM 5,5-dithio-bis-(2-nitrobenzoic acid) (Sigma-Aldrich) and 2 mg/mL bovine serum albumin (Sigma-Aldrich) diluted in PAC Sample Buffer from the purification step. Immediately after mixing, the absorbance at 412 nm was measured every minute in a SPECTROstar Nano (BMG Labtech) at ambient temperature. Optimum PAC concentrations were individually determined using a dilution series to identify a concentration that had a linear response within the first 20 min. The initial velocity of the data was normalised to protein concentration and expressed as percent activity relative to wild-type PAC.

### Mammalian cell culture and transfection

Freestyle 293-F (Thermo Fisher Scientific) and HEK293T cells were grown as adherent cultures in Dulbecco's Modified Eagle Medium (DMEM, Thermo Fisher Scientific) with 10% (v/v) foetal bovine serum (Serana) and 1X GlutaMAX Supplement (Thermo Fisher Scientific) in a humidified incubator at 37 °C and 5% CO_2_.

For both transient and stable transfection, 4 × 10^5^ cells in 2 mL were seeded into a 6-well tissue culture plate the day before transfection. Prior to transfection, the culture volume was reduced to 1 mL and transfection performed using 1 μg plasmid DNA and 4 μL of FuGENE Transfection Reagent (Promega) according to the recommended protocol.

For the generation of stably transfected cell pools, 293-F cells were washed 24 h after transfection with 1X PBS and detached using 500 μL Accutase (Sigma-Aldrich) for 5 min at 37 °C. After the addition of 1 mL DMEM plus 10% FBS, 700 μL of this suspension was diluted in 12 mL of DMEM plus 10% FBS. 2 mL of the suspension was then dispensed into each well of 6-well plate and incubated overnight. Drug selection was performed with puromycin (0–5 μg/mL) and the cells incubated for 14 days with one media change. To assess colony formation of surviving clones, a subset of transfectants were fixed and stained with glutaraldehyde/crystal violet solution (Sigma-Aldrich).

Parallel experiments were set up in duplicate so that wells that showed colony formation by staining at 14 days were used to expand polyclonal pools in the other replicate for downstream experiments. Cells were transferred into T75 flasks and cultured with the same concentration of puromycin. When 60–80% confluent, the selected pools were harvested and stored in liquid nitrogen vapour phase in a solution of DMEM plus 10% FBS with 7.5% DMSO.

### Cell proliferation

The growth rate analysis of stably transfected 293-F pools grown in 2 μg/mL puromycin in DMEM with 10% FCS was performed in 96-well plates using the AlamarBlue Assay (Thermo Fisher Scientific) according to the manufacturer’s protocol.

### ELISA

A 96-well plate (Nunc MaxiSorp flat-bottom, Thermo Fisher Scientific) was coated overnight with 2 μg/mL pertuzumab in 100 μL PBS at 4 °C. The plate was washed twice with PBS + 0.05% Tween-20 (PBS-T) and blocked for 1.5 h by the addition of 150 μL 2% skim milk dissolved in wash buffer. For the calibration curve, 100 μL of purified, FLAG-tagged recombinant ErbB2 ectodomain (residues 1–623) dissolved in PBS was applied for 2 h at concentrations of 215, 200, 175, 150, 125, 100, 75 and 65 ng/mL. As a negative control, 1 μg of purified, FLAG-tagged recombinant epidermal growth factor receptor (EGFR) ectodomain was used. The wells were then washed with PBS-T, and cell culture supernatants serially diluted in PBS were added and incubated for 2 h. The plate was washed three times with PBS-T buffer and 100 μL of detection antibody (anti-FLAG-HRP, Sigma-Aldrich), diluted 1:12,000 in the wash buffer, added for 2 h. The plate was washed four times with PBS-T followed by incubation with 100 μL of a 1:1 Luminol/Peroxide solution (BD OptEIA TMB substrate reagent set, BD Biosciences). Absorbance was measured in a CLARIOstar plate reader at 450 nm (BMG Labtech).

## Supplementary Information

Supplementary Information.
