# Supplementary material for: Structure-guided selection of puromycin N-acetyltransferase mutants with enhanced selection stringency for deriving mammalian cell lines expressing recombinant proteins
Source: Sci Rep. 2021 Mar 4;11:5247. doi: 10.1038/s41598-021-84551-9 (PMC7933286; doi:10.1038/s41598-021-84551-9)
Supplement: Supplementary file 1 — Supplementary Information. [file 41598_2021_84551_MOESM1_ESM.docx]

**Supplementary Information**

Structure-guided selection of puromycin *N*-acetyltransferase mutants with enhanced selection stringency for deriving mammalian cell lines expressing recombinant proteins.

Alessandro T. Caputo, Oliver M. Eder, Hana Bereznakova, Heleen Pothuis, Albert Ardevol-Grau, Janet Newman, Stewart Nuttall, Thomas S. Peat and Timothy E. Adams

Supplementary Methods:

*Test Expression of PAC mutants*

One day after transfection, HEK 293T cells were exchanged into 1 mL of Freestyle 293 Expression medium (Thermo Fisher Scientific) and the cells incubated for a further 2 days. The media was collected, and the remaining cells were lysed by addition of ice-cold TX-100 buffer (50 mM Tris-HCL pH7.4, 150 mM NaCl, 10% v/v glycerol, 1 mM EDTA, 1% v/v Triton X-100) for 30 minutes on ice. The levels of PAC and ErbB2 in the lysates and ErbB2 in the media were compared by immunoblotting. Samples were normalized for whole protein content measured with Pierce BCA Protein Assay Kit (Thermo Fisher Scientific).

*Immunoblotting*

Samples were separated on 4-12% Bis-Tris SDS-PAGE gels (Thermo Fisher Scientific) and proteins were transferred using the Blot system (Thermo Fisher Scientific). The membrane was blocked for 1 hour with 1% casein (w/v) in Tris-buffered saline (TBS) and incubated with the indicated primary antibody for 2 h at room temperature. The primary antibody concentrations were as follows: anti-FLAG-HRP (1:2000; Sigma-Aldrich); anti-beta actin (1:1000; Invitrogen); anti-Myc HRP-conjugated antibody (1:1000; Cell Signalling Tech., Mouse mAB 9B11). The secondary antibody of rabbit anti-mouse HRP-conjugated polyclonal antibody (1:10000; Sigma-Aldrich) was added for anti-beta actin detection. SuperSignal West Pico Plus chemiluminescent substrate (Thermo Scientific Scientific) was used and imaged in an Odyssey Fc imaging system (Li-Cor).

*Production of Pertuzumab*

Synthetic DNA templates encoding the heavy and the light chains of the humanized anti-ErbB2 IgG1 antibody, pertuzumab (Drugbank Accession Number DB06366), codon-optimized for translation in human cells, were synthesised and subcloned into the pCAGGS mammalian expression vector (Genscript). Freestyle 293-F cells (Thermo Fisher Scientific) grown in Freestyle 293 Expression medium were co-transfected using a polyethylenimine (PEI):DNA mixture containing 250 μg of both heavy and light chain constructs. Cells were harvested after nine days, filtered, and the conditioned supernatant was passed through a 1 mL HiTrap MabSelect PrismA column (GE Healthcare). The bound antibody was eluted with a low pH buffer (0.1 M sodium citrate, pH 3.0, 150 mM NaCl) and immediately applied to a HiPrep 26/10 Desalting column (GE Healthcare) equilibrated with PBS.

*Reverse transcriptase-polymerase chain reaction (RT-PCR)*

In a 12-well plate (Nunclon™ Delta Surface, Thermo Scientific) stable pools of 2.2 x 10^5^ Freestyle 293-F cells stable pools transfected with pM18s-PAC-ErbB2 constructs were seeded in triplicate with 2 μg/mL puromycin added. The cells were grown for 3 days before the supernatant was discarded, the cells were washed with 1 mL 1X PBS and whole RNA was isolated as described in the TRIzol (Gibco BRL) reagent datasheet. An additional step to remove DNA contamination was undertaken with the DNA-free Kit (Ambion) according to the manufacturer's instructions. Primers were designed to amplify cDNA corresponding to *pac*, the *pac* + *erbB2* cassette, and endogenous *gapdh* as loading control (Supplementary Table S5) in one reaction using the OneTaq One-Step RT-PCR Kit (NEB). These primers are all inserted into one master mix. The final concentrations of primers were: *pac* forward 350 nM, *pac* reverse 170 nM, *pac* + *erbB2* reverse 170 nM, *gapdh* forward 130 nM, *gapdh* reverse 130 nM. Each reaction contained 150 ng of total RNA and RT performed at 48°C for 30 minutes. For the first 10 cycles, a touchdown approach was programmed with a temperature ramp decreasing 0.6°C per cycle followed by 20 cycles at the final annealing temperature.

Supplementary Figures


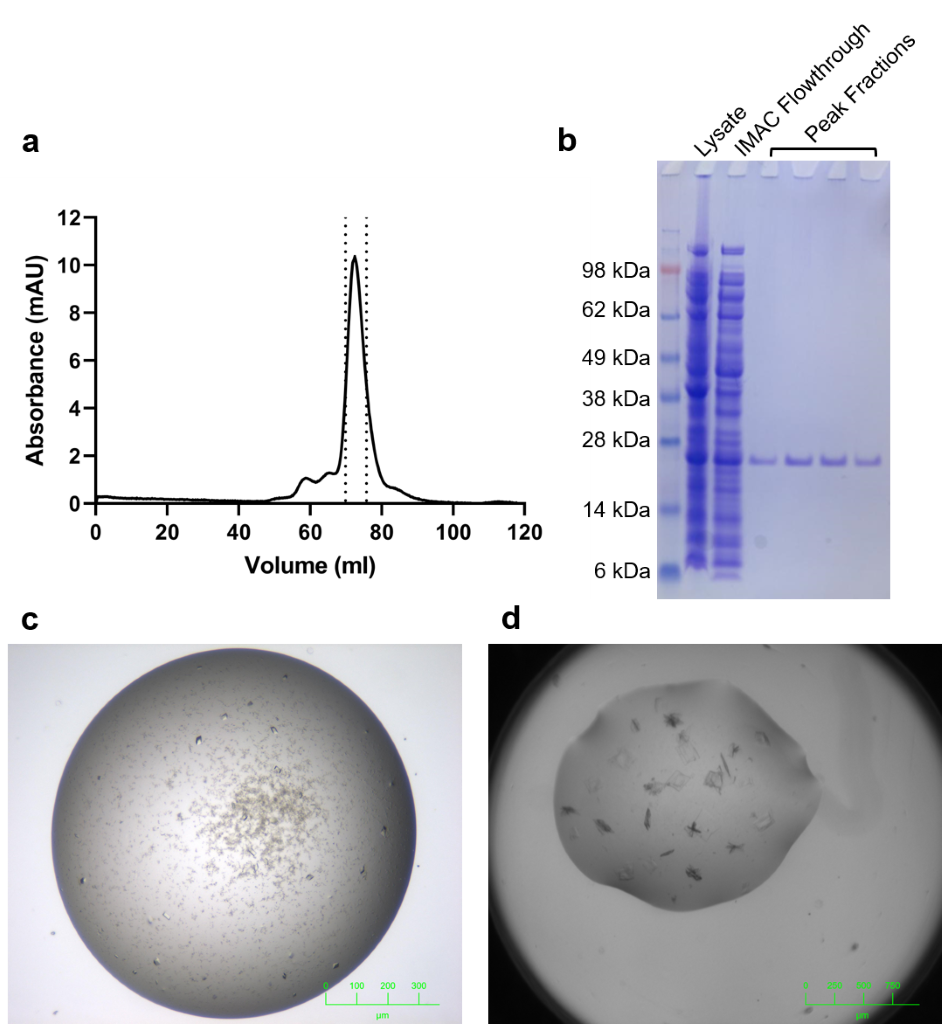


Supplementary Figure S1: Expression, purification and crystallisation of PAC. (**a**) Size-exclusion chromatogram from a Superdex 75 16/600 column of the wild-type PAC at the final step of purification. (**b**) SDS-PAGE analysis of the cell lysate before and after the IMAC binding step and the size-exclusion chromatography fractions indicated by dotted vertical lines in panel (**a**). Crystallisation experiments that were used in this study (**c**) for the acetyl-CoA bound structure and (**d**) the CoA/acetyl-puromycin structure.


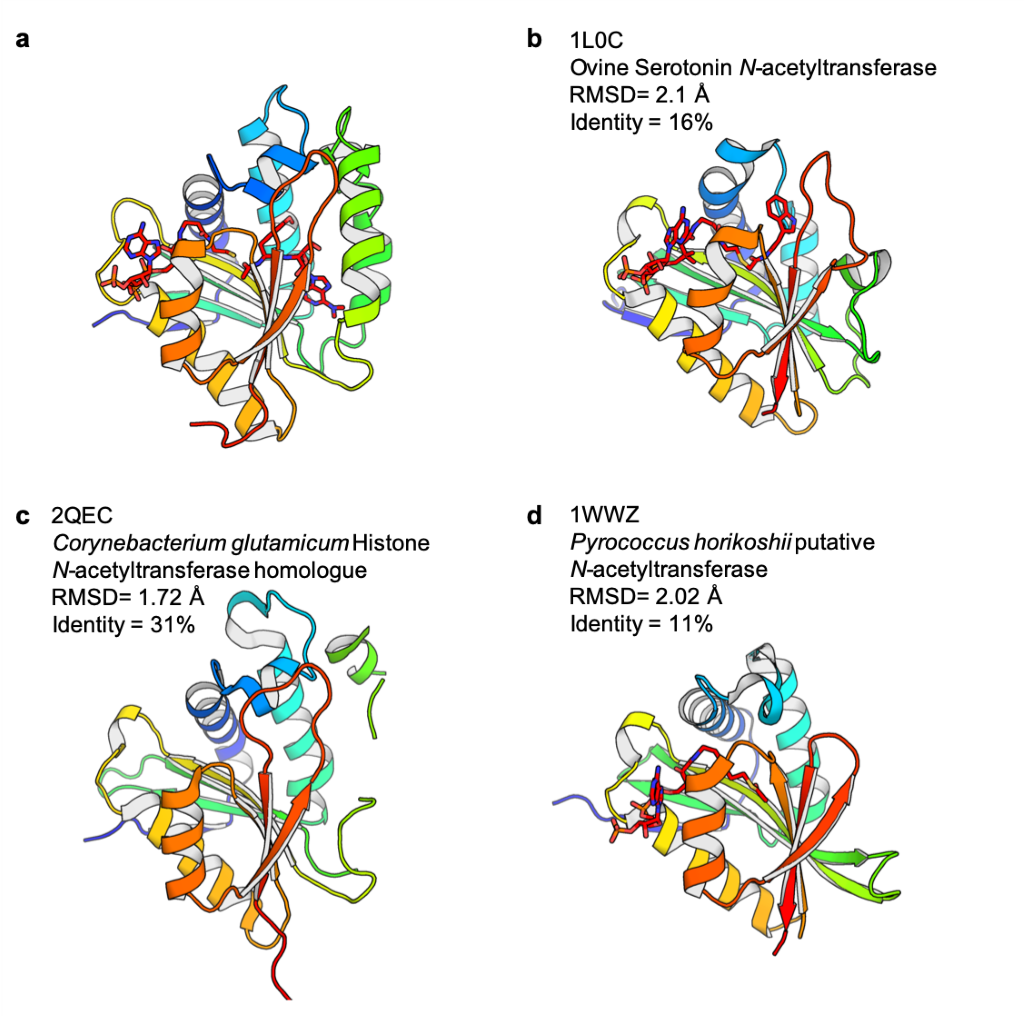


Supplementary Figure S2: Structural similarity of PAC to other GNAT enzymes in rainbow colouring from blue (N-terminus) to red (C-terminus). Structures were overlayed and presented in the following grid: (**a**) PAC and (**b-d**) Structurally and catalytically related structured identified using PDBeFold or HHPred^1–3^.


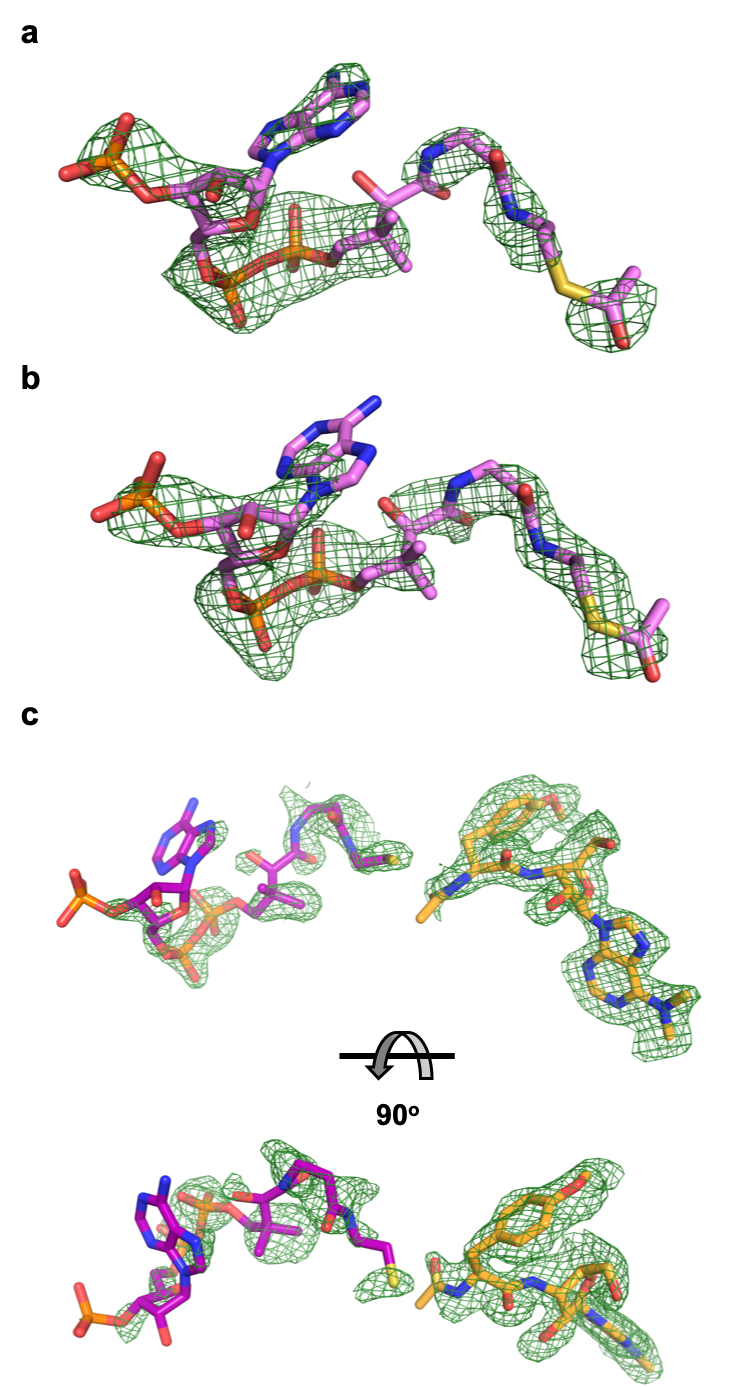


Supplementary Figure S3: Omit maps of observed ligands in the active site of PAC. (a,b) Two representative conformations of AcCoA for the six copies in the asymmetric unit. The correlation coefficients to the final 2mF_o_-DF_c_ maps are 0.924 and 0.925 respectively. (c) CoA and acetylated puromycin. Difference maps are contoured at 3σ.


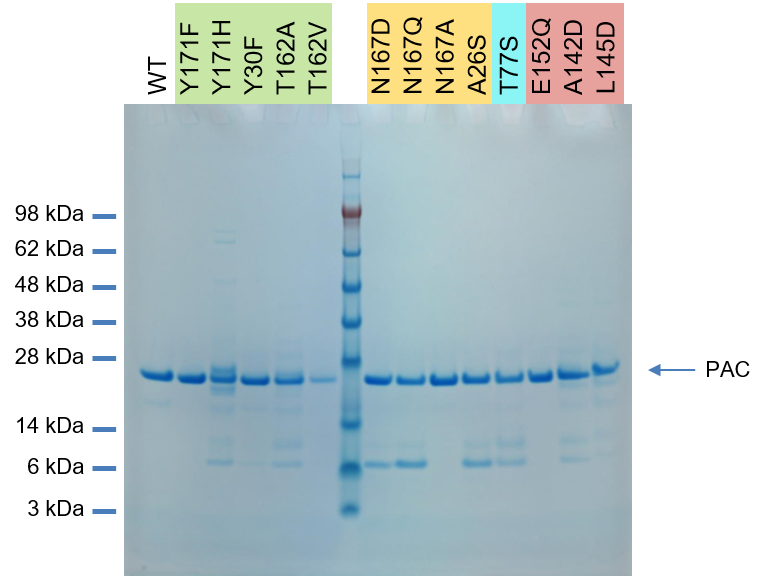


Supplementary Figure S4: Coomassie Brilliant Blue stained SDS-PAGE of purified PAC proteins used for activity measurements. Colour coding is consistent with the classes of mutations described in Figure 2.


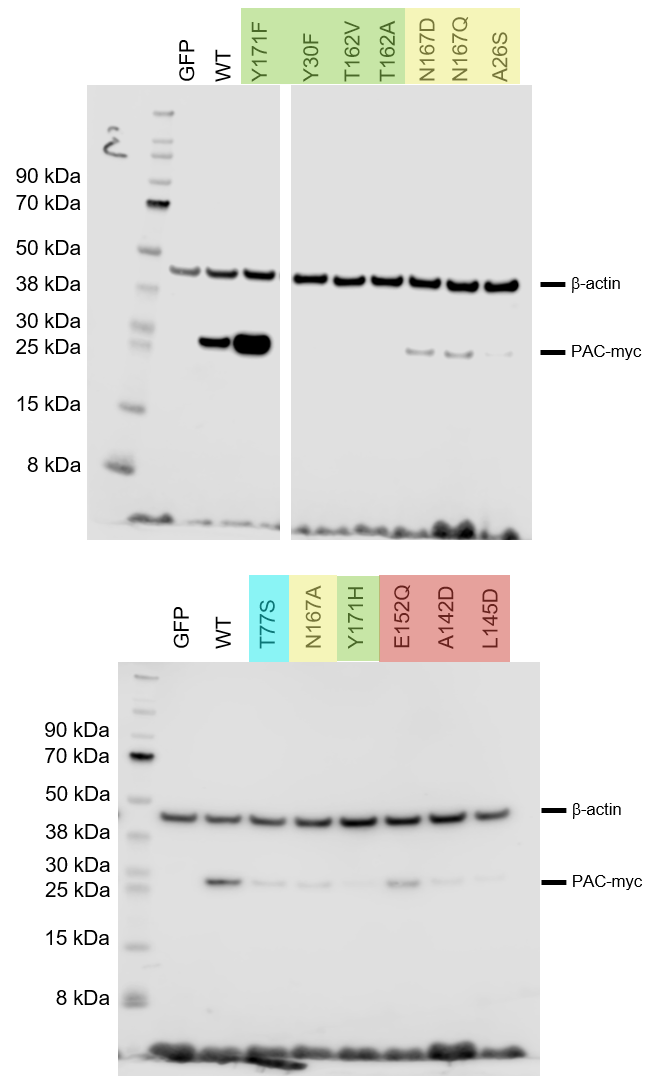


Supplementary Figure S5: Immunoblotting of transient transfections of PAC mutants in HEK293-T cells. Blots were probed with a mixture of anti-β-actin and anti-myc antibodies as described in the Supplementary Methods. Colour coding is consistent with the classes of mutations described in Figure 2.

Supplementary Tables:

Supplementary Table S1: Data collection and refinement statistics of PAC crystal structures.

|  | AcCoA co-crystal (PDB ID:7K09) | CoA/Ac-puromycin co-crystal (PDB ID: 7K0A) |
| --- | --- | --- |
| Data Collection |  |  |
| Space Group | P2_1_2_1_2 | I2 |
| Cell Dimensions |  |  |
| *a, b, c* (Å) | 148.6, 73.2, 106.0 | 36.6, 141.5, 99.0 |
| α, β, γ (^o^) | 90, 90, 90 | 90, 101.0, 90 |
| Resolution (Å) | 106.24-2.36 (2.59-2.36) | 70.75-2.00 (2.05-2.00) |
| Anisotropic high-resolution ellipsoid axes | 2.57, 3.58, 2.30 | NA |
| R_meas_ | 0.517 (3.226) | 0.400 (1.705) |
| R_pim_ | 0.125 (0.795) | 0.136 (0.608) |
| I/σI | 7.7 (1.5) | 7.7 (2.3) |
| CC_1/2_ | 0.994 (0.504) | 0.989 (0.857) |
| Completeness (%) | 93.1 (67.0) | 99.9 (99.4) |
| Multiplicity | 16.9 (15.8) | 17.2 (15.2) |
|  |  |  |
| Refinement |  |  |
| Resolution (Å) | 106.24-2.31 | 42.49-2.00 |
| No. of unique reflections | 27849 | 31752 |
| R_work_/R_free_ | 0.2363/0.2523 | 0.2071/0.2405 |
| No. of atoms |  |  |
| Protein | 8829 | 2996 |
| Ligands | 306 | 226 |
| Water | 24 | 273 |
| B-factors (Å^2^) |  |  |
| Protein | 45.5 | 16.7 |
| Ligand | 36.1 | 34.1 |
| Water | 16.2 | 24.8 |
| RMSD bond lengths (Å) | 0.01 | 0.01 |
| RMSD bond angles (^o^) | 1.06 | 1.62 |
| Ramachandran favoured/outliers (%) | 97.7/0.0 | 97.4/0.3 |
| Clashscore | 2.82 | 3.65 |
| MolProbity Score | 1.48 | 1.30 |

*Highest resolution shell is shown in parentheses.

**Anisotropic resolution limits with ellipsoid axis values

Supplementary Table S2: Relative enzyme activity of PAC mutants. “n” is the number of measurements carried out on separate days which have been averaged from technical triplicates. P values derived using a one-way ANOVA relative to wild-type values.

|  | Mutated residue | Mean %activity of WT^*^ | Standard deviation | n | Adjusted P Value |
| --- | --- | --- | --- | --- | --- |
|  | WT | 100 | 22.6 | 17 |  |
| Catalytic site | Y171F | 3.61 | 1.25 | 4 | <0.0001 |
|  | Y171H | 0.28 | 0.15 | 5 | <0.0001 |
|  | Y30F | 51.1 | 6.40 | 3 | <0.0001 |
|  | T162V | 15.7 | 14.2 | 7 | <0.0001 |
|  | T162A | 1.04 | 0.36 | 6 | <0.0001 |
| Acetyl-CoA Binding | N167D | 0.90 | 0.37 | 4 | <0.0001 |
|  | N167Q | 8.01 | 5.10 | 7 | 0.001 |
|  | N167A | 147.3 | 4.78 | 3 | <0.0001 |
|  | A26S | 69.9 | 26.5 | 6 | <0.0001 |
| Puromycin binding | T77S | 10.01 | 1.91 | 3 | <0.0001 |
| Alteration of electrostatic potential | E152Q | 107.3 | 6.1 | 4 | 0.9991 |
|  | A142D | 4.05 | 0.92 | 4 | <0.0001 |
|  | L145D | 1.79 | 0.60 | 5 | <0.0001 |
| Experimental control | Empty plasmid control identical purification of BL21 (DE3) *E. coli* | 0.035 | 0.001 | 2 | <0.0001 |

* $Relative Activity= \frac{\Delta A_{412}/min/{mg}_{mut}}{\Delta A_{412}/min/{mg}_{WT}} X \frac{100}{1}$

Supplementary Table S3: The thermal stability of the isolated PAC mutants assessed by differential scanning fluorimetry. PAC mutants were tested in three different conditions, buffer, 0.2 mM AcCoA in buffer or 0.2 mM puromycin in buffer. Delta values relative to wild-type of identical buffer/additive condition. Each of the proteins were measured in triplicate for each condition ± standard deviation. “nd” indicates where no detectable transition was measurable.

|  | **Mutated residue** |  | **T_m_ (°C) Buffer only** | **ΔT_m_ (°C) Buffer only** | **T_m_ (°C) +Acetyl-CoA** | **ΔT_m_ (°C) +Acetyl-CoA** | **T_m_ (°C) +Puromycin** | **ΔT_m_ (°C) +Puromycin** |
| --- | --- | --- | --- | --- | --- | --- | --- | --- |
|  | WT |  | 33.41 ± 2.77 |  | 45.83 ± 1.57 |  | 45.57 ± 0.62 |  |
| Catalytic site | Y171F |  | 46.19 ± 0.04 | 12.78 | 53.81 ± 0.02 | 7.97 | 46.02 ± 0.06 | 0.45 |
|  | Y171H |  | 53.87 ± 0.65 | 20.46 | 53.75 ± 0.33 | 7.91 | 55.49 ± 0.51 | 9.92 |
|  | Y30F |  | nd | nd | 40.13 ± 0.04 | -5.71 | 38.69 ± 0.10 | -6.88 |
|  | T162V |  | nd | nd | 38.73 ± 0.07 | -7.11 | 43.27 ± 0.05 | -2.30 |
|  | T162A |  | nd | nd | nd | nd | nd | nd |
| Acetyl-CoA Binding | N167D |  | 30.18 ± 2.70 | -3.23 | 28.19 ± 0.12 | -17.65 | 47.28 ± 0.14 | 1.71 |
|  | N167Q |  | 34.95 ± 0.05 | 1.54 | 42.31 ± 0.15 | -3.53 | 47.00 ± 0.03 | 1.43 |
|  | N167A |  | 27.99 ± 3.80 | -5.42 | 42.69 ± 0.11 | -3.15 | 45.85 ± 0.13 | 0.28 |
|  | A26S |  | 27.65 ± 0.13 | -5.76 | 43.63 ± 0.09 | -2.21 | 44.06 ± 0.04 | -1.51 |
| Puromycin binding | T77S |  | nd | nd | nd | nd | nd | nd |
| Alteration of electrostatic potential | E152Q |  | 35.52 ± 0.02 | 2.11 | 45.69 ± 0.07 | -0.15 | 44.35 ± 0.02 | -1.22 |
|  | A142D |  | nd | nd | nd | nd | nd | nd |
|  | L145D |  | nd | nd | nd | nd | nd | nd |

Supplementary Table S4: Colony formation assay of PAC mutants in Freestyle 293-F cells. Transfectants selected with indicated concentrations of puromycin and grown for 14 days before fixing and staining.

| Mutated  residue | 0 µg/mL | 1 µg/mL | 2 µg/mL | 3 µg/mL | 4 µg/mL | 5 µg/mL |
| --- | --- | --- | --- | --- | --- | --- |
| GFP control | 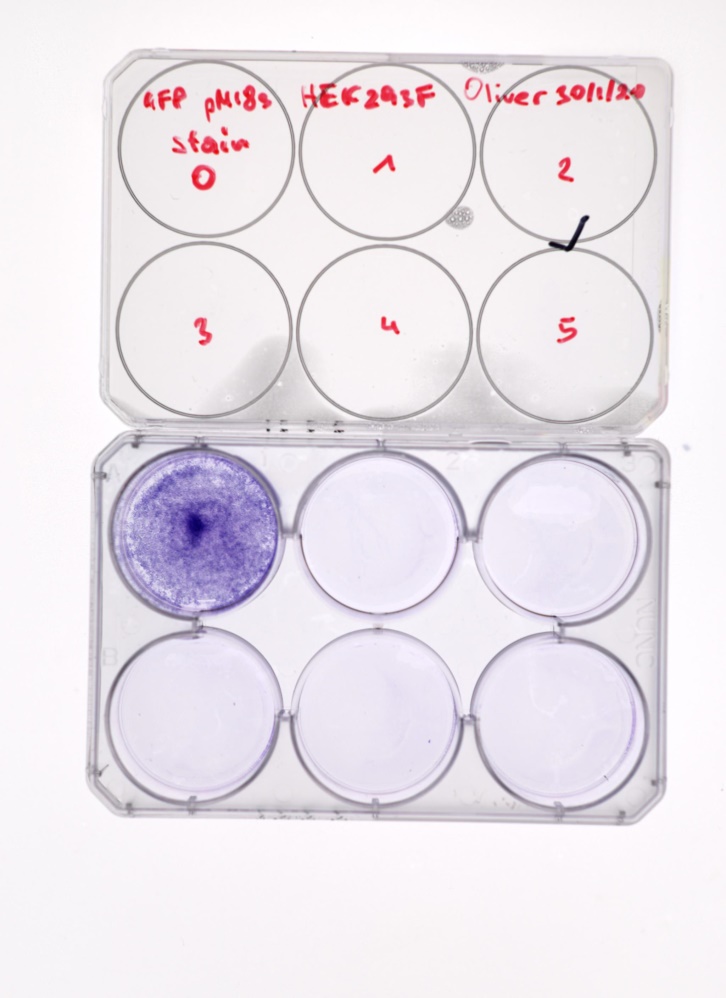 | 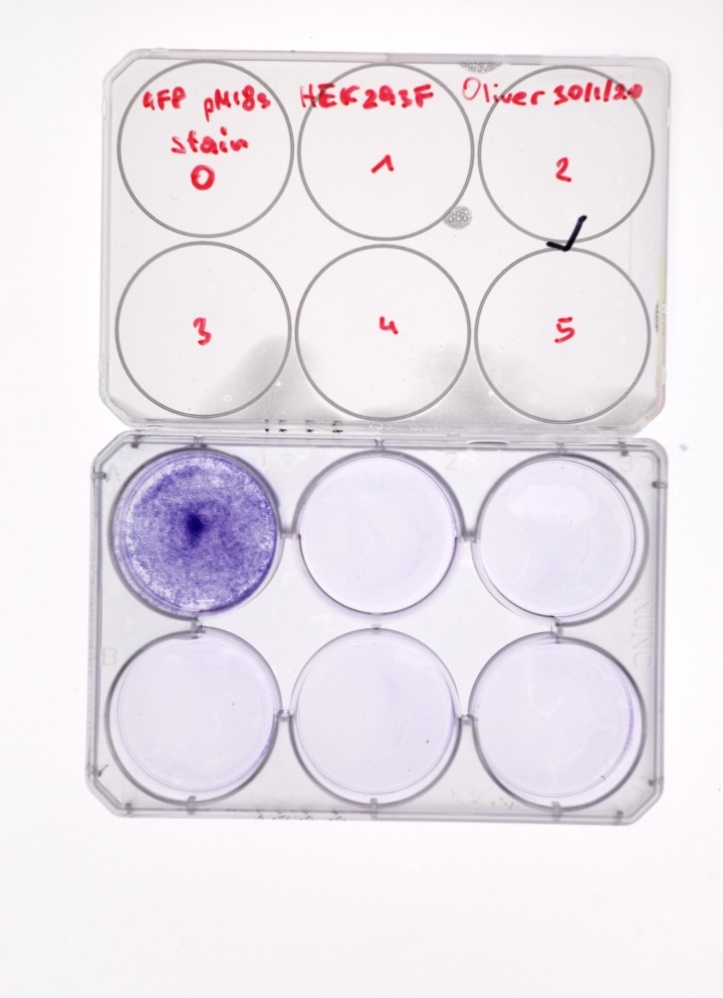 | 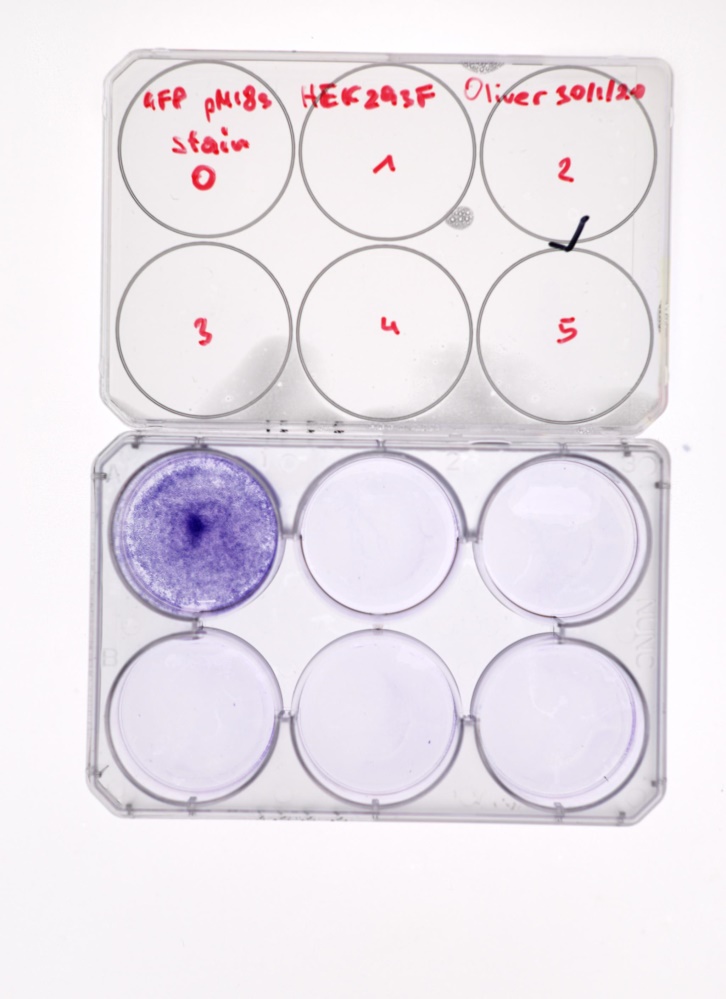 | 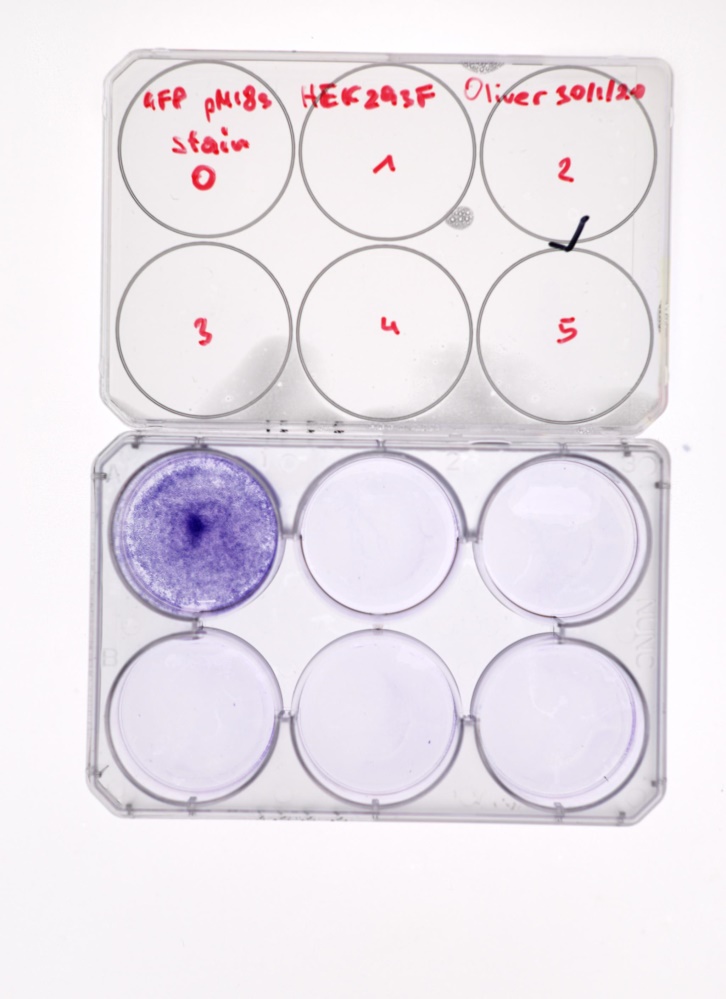 | 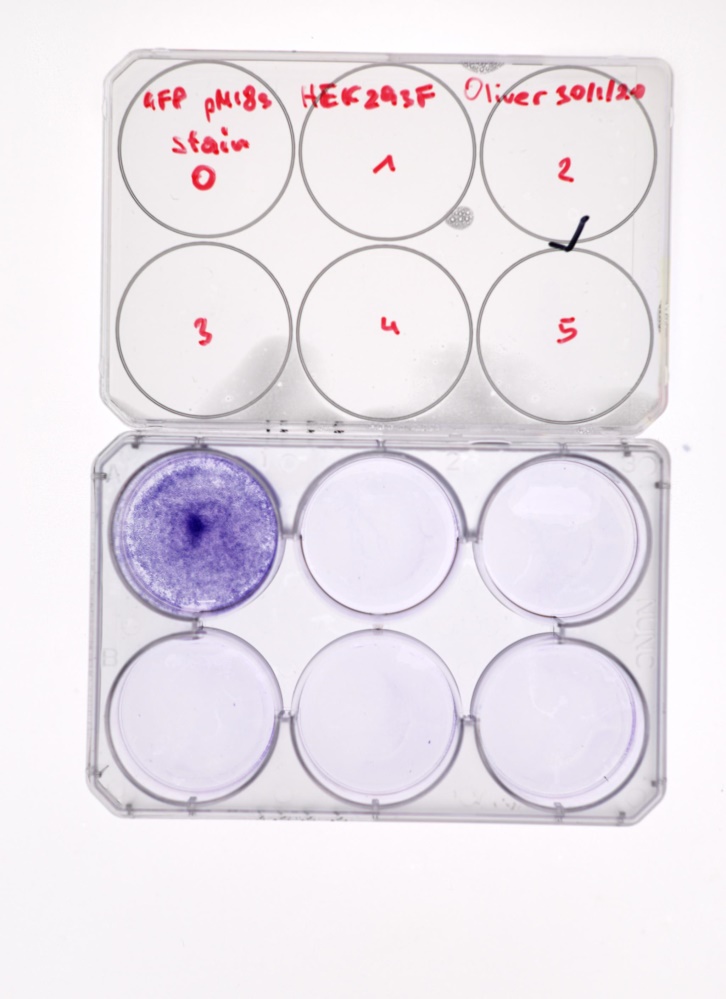 | 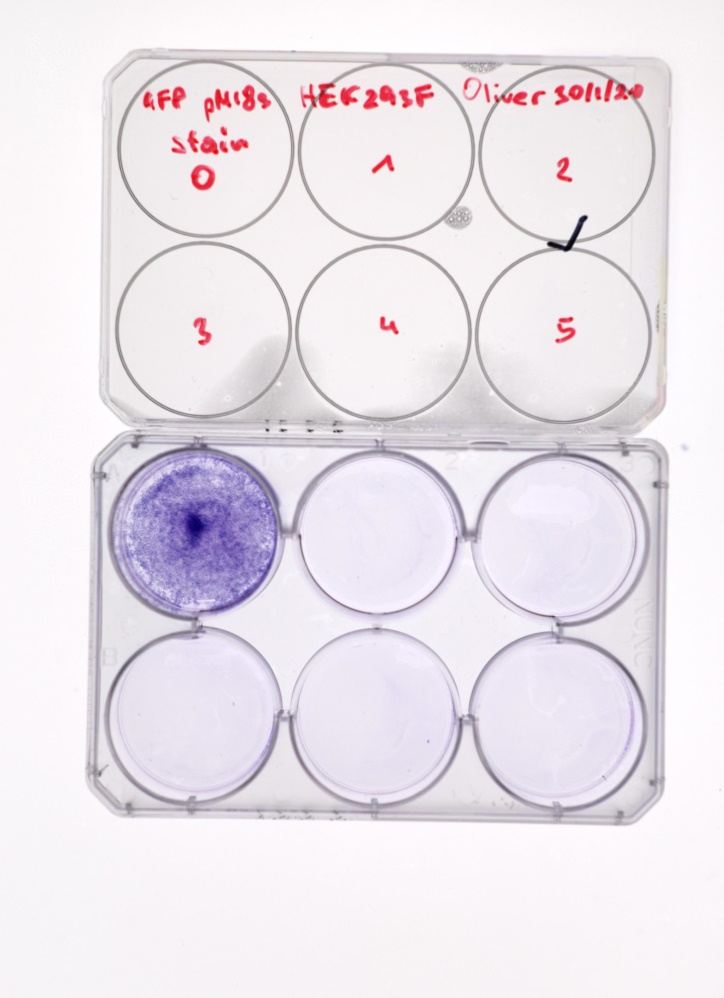 |
| WT | 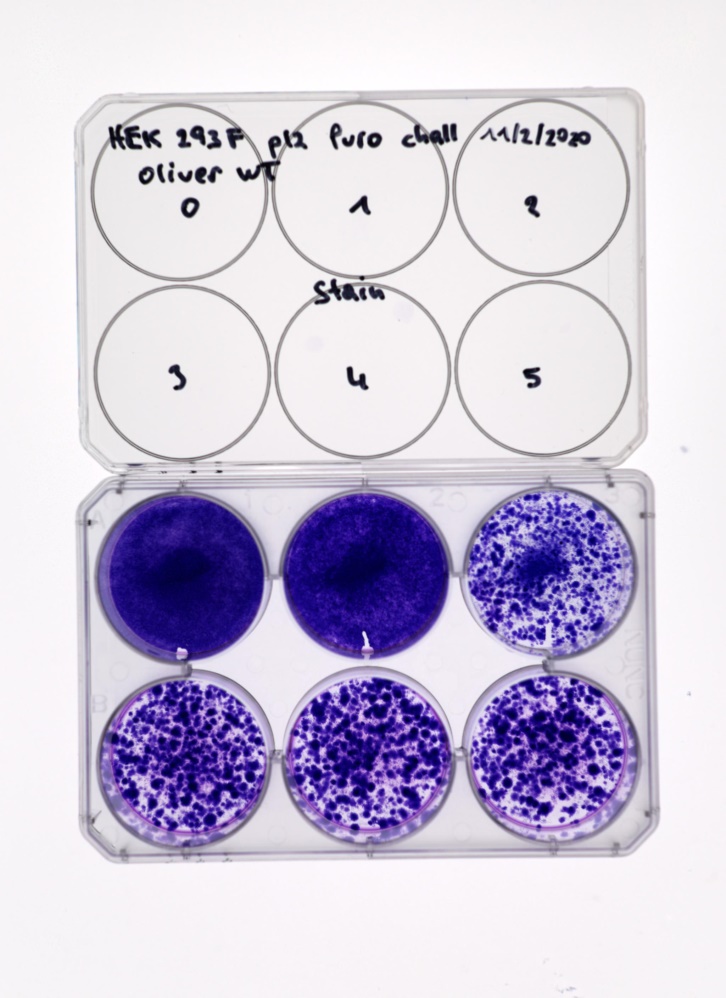 | 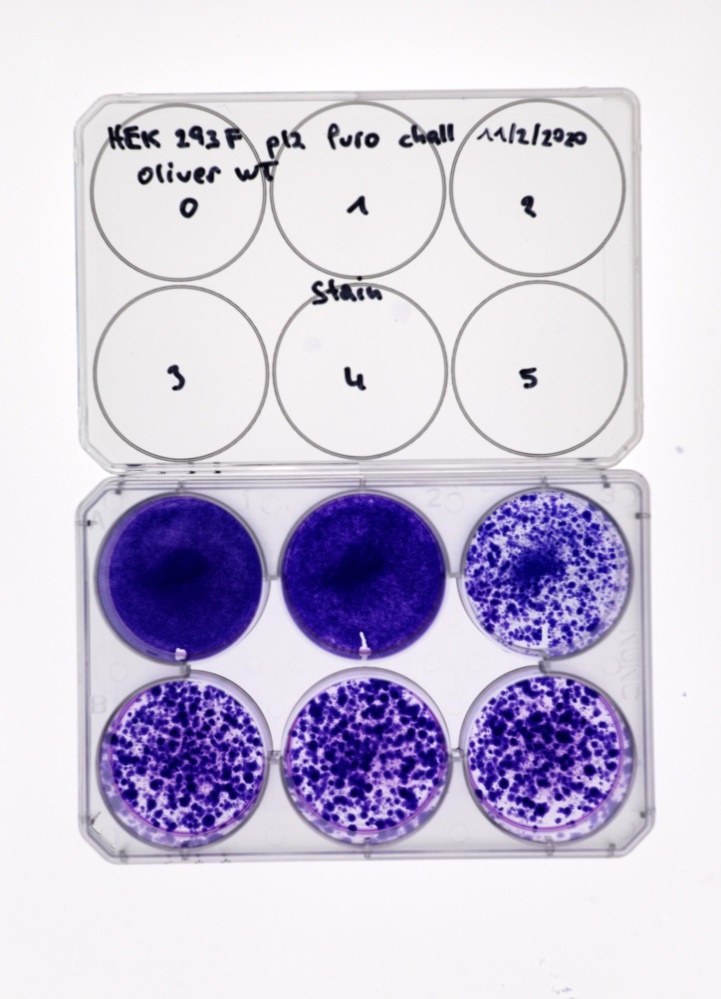 | 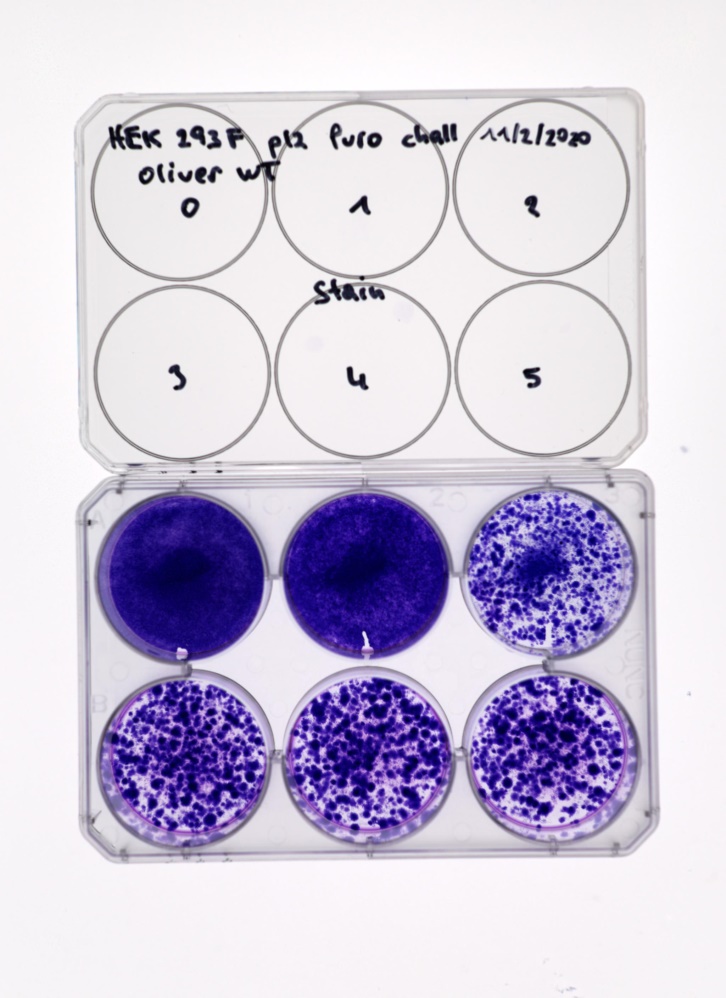 | 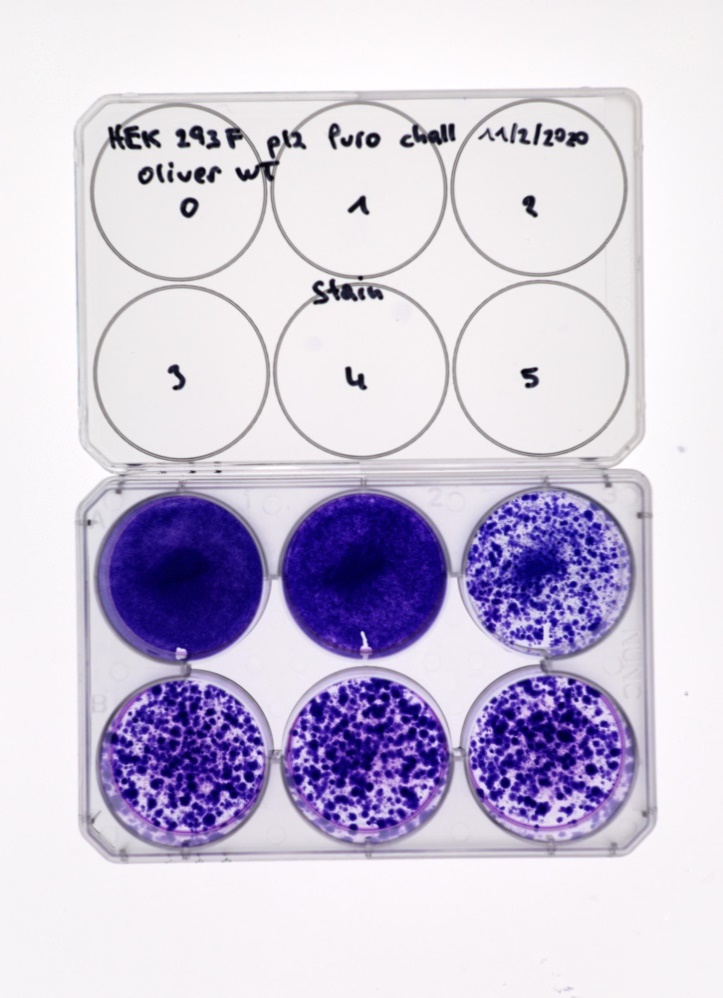 | 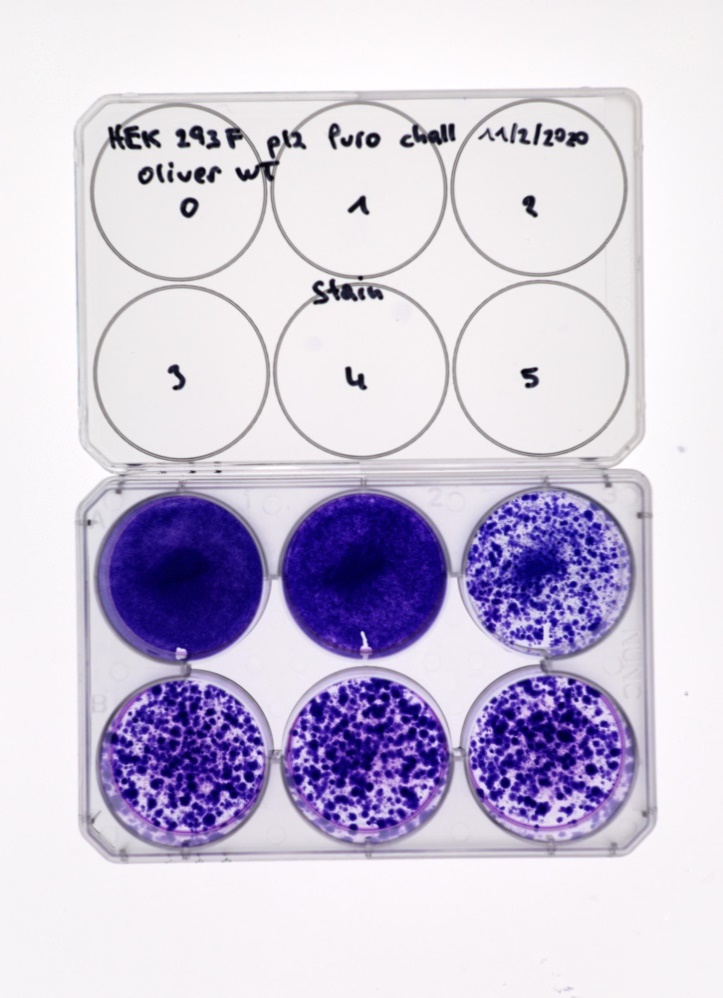 | 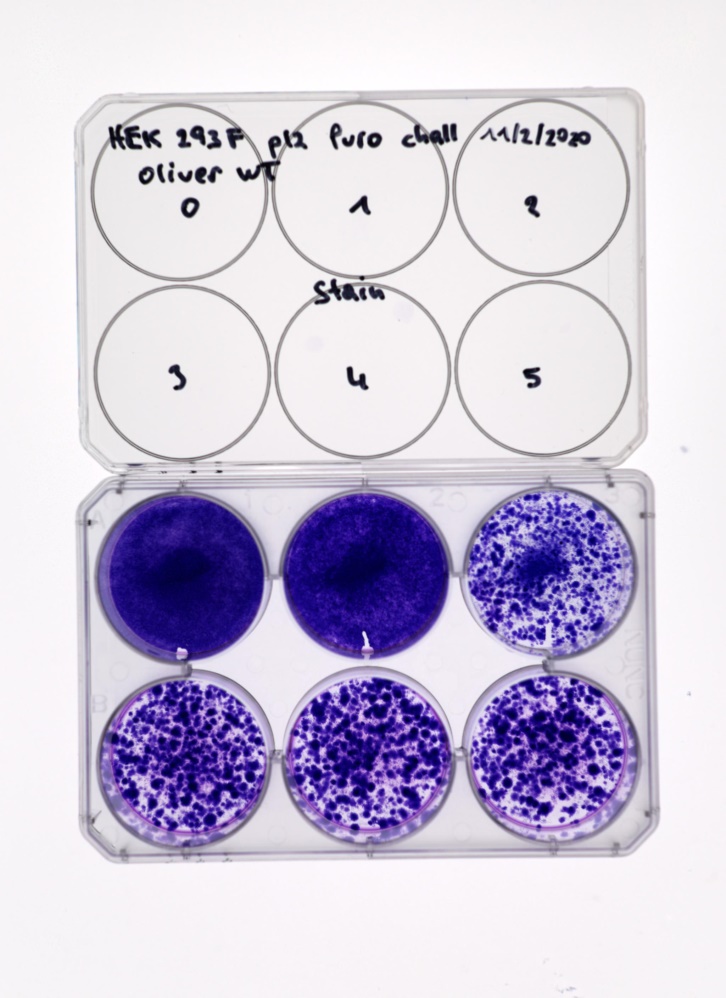 |
| Y171F | 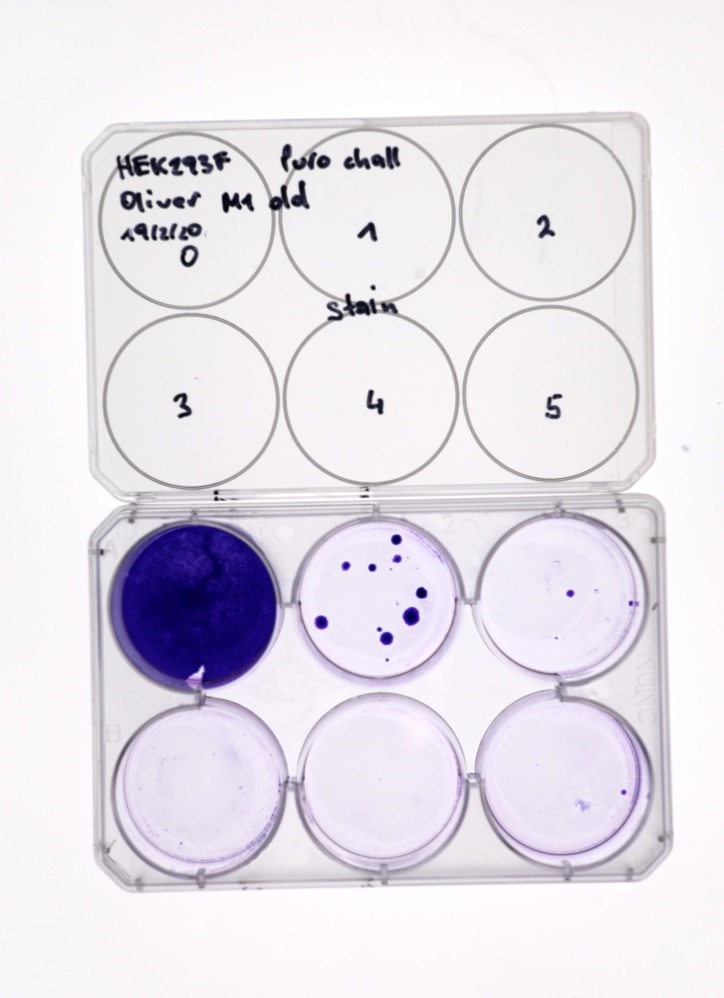 | 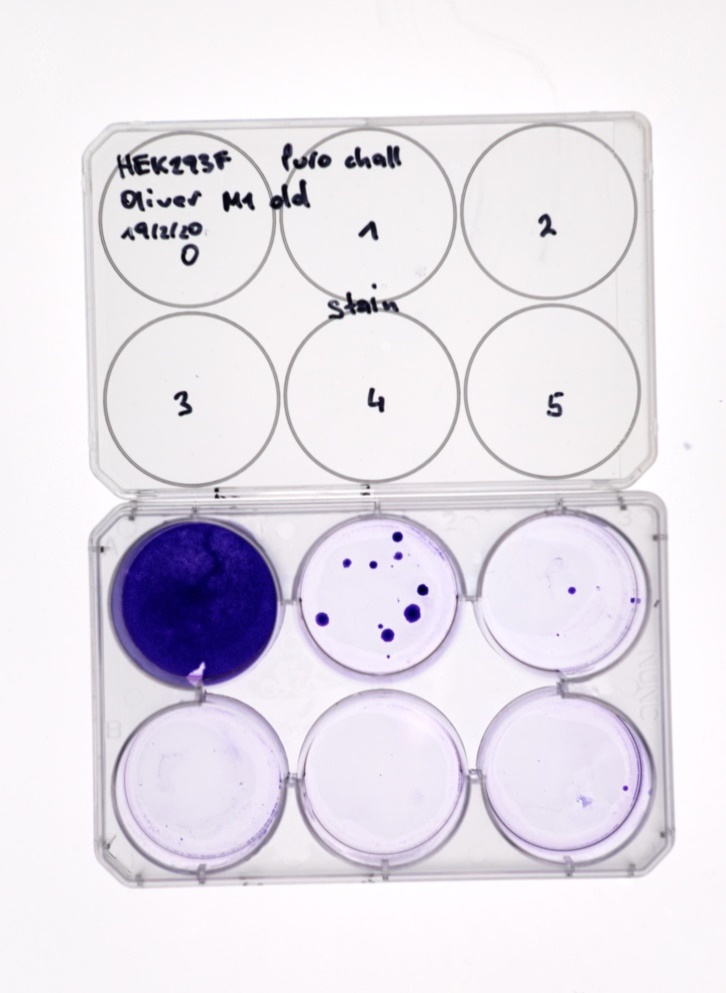 | 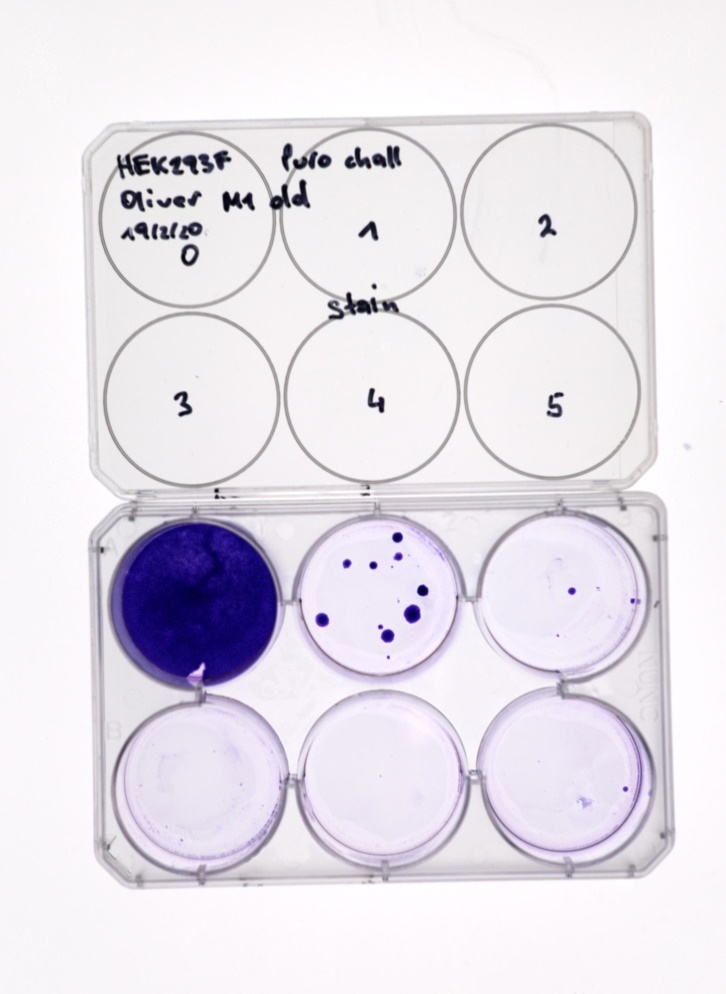 | 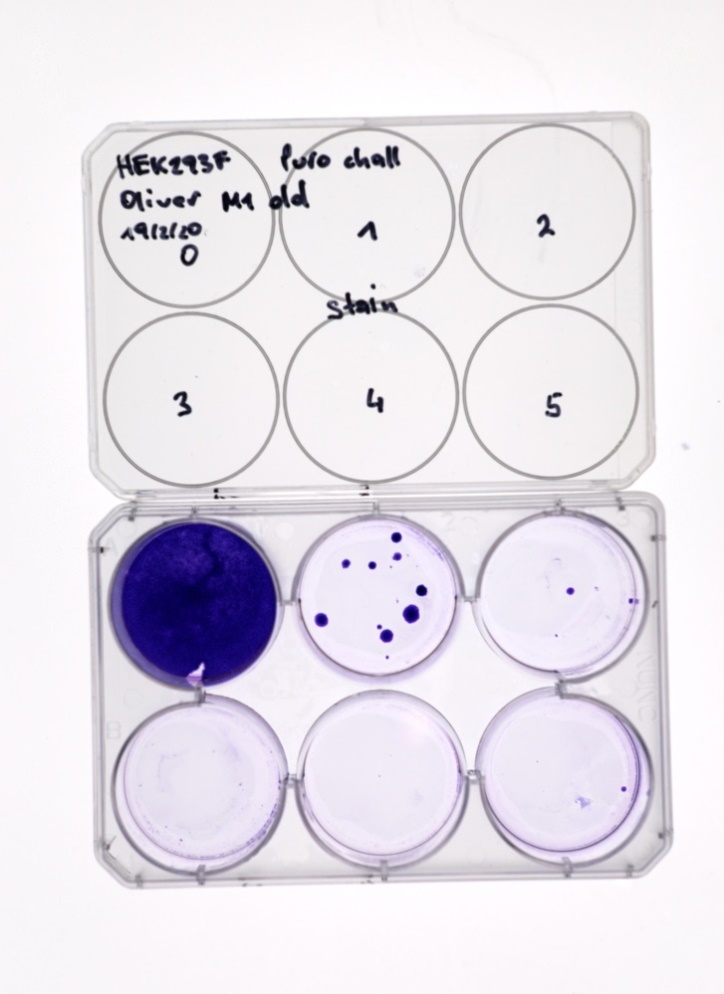 | 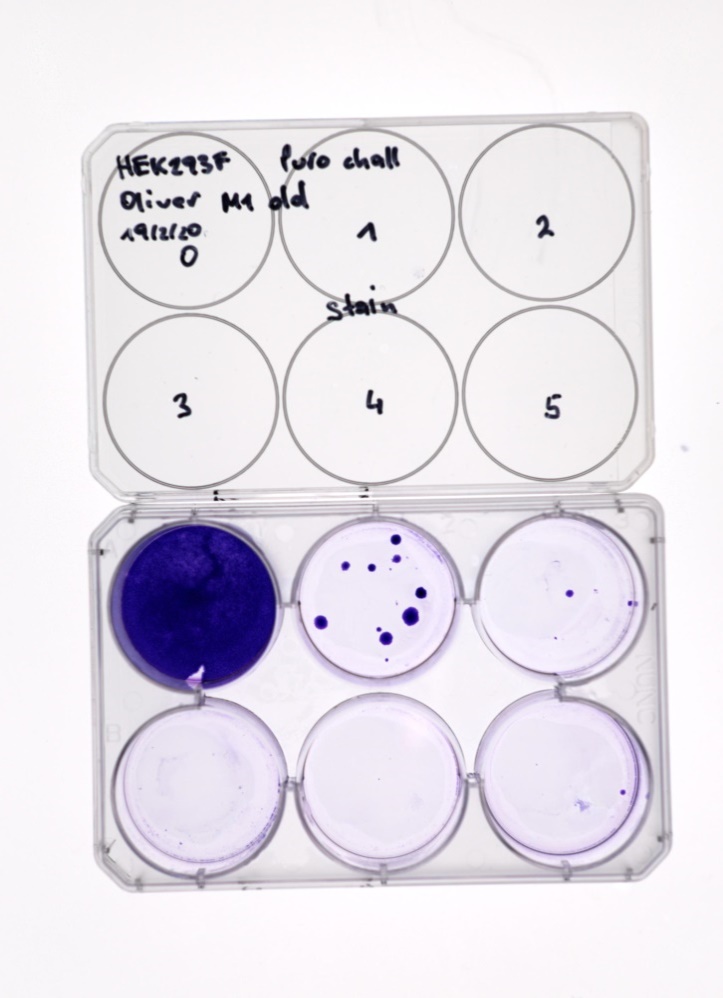 | 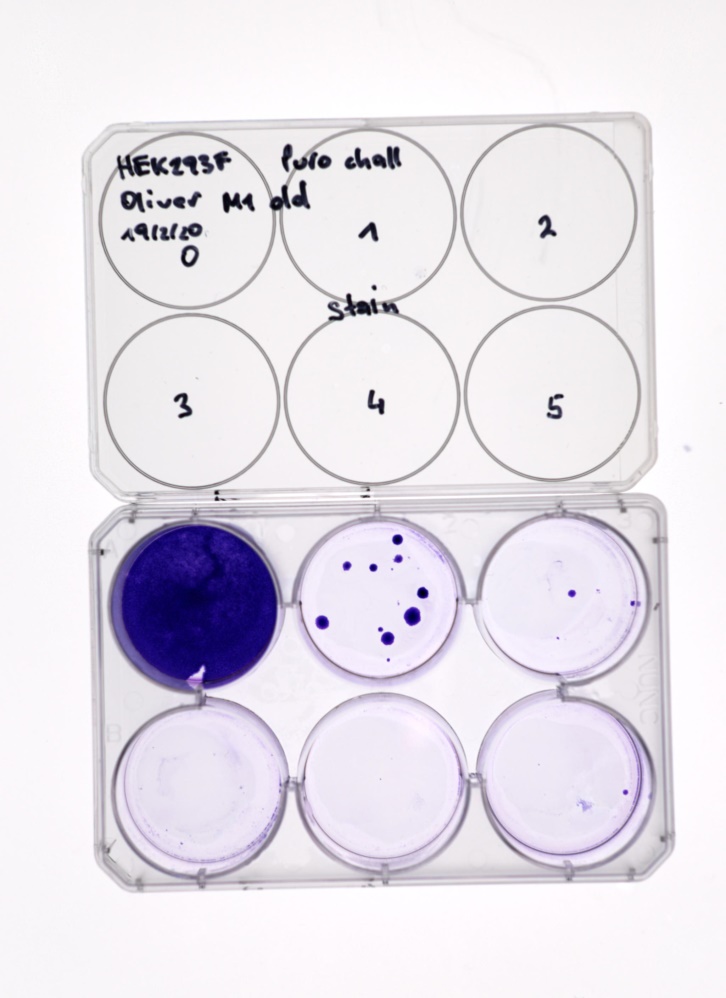 |
| Y171H | 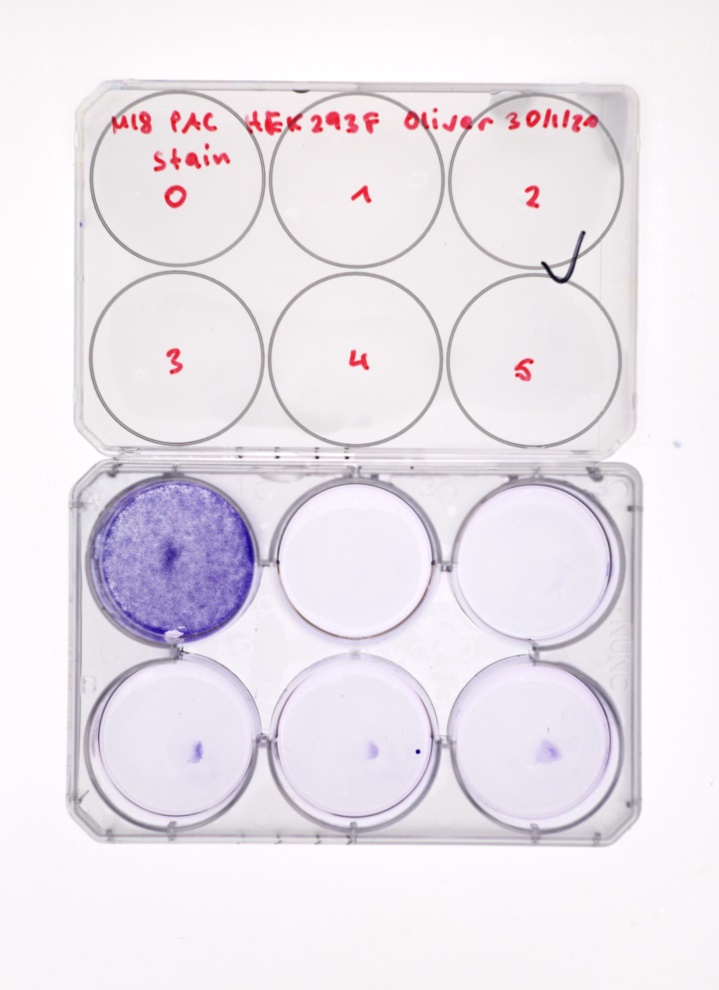 | 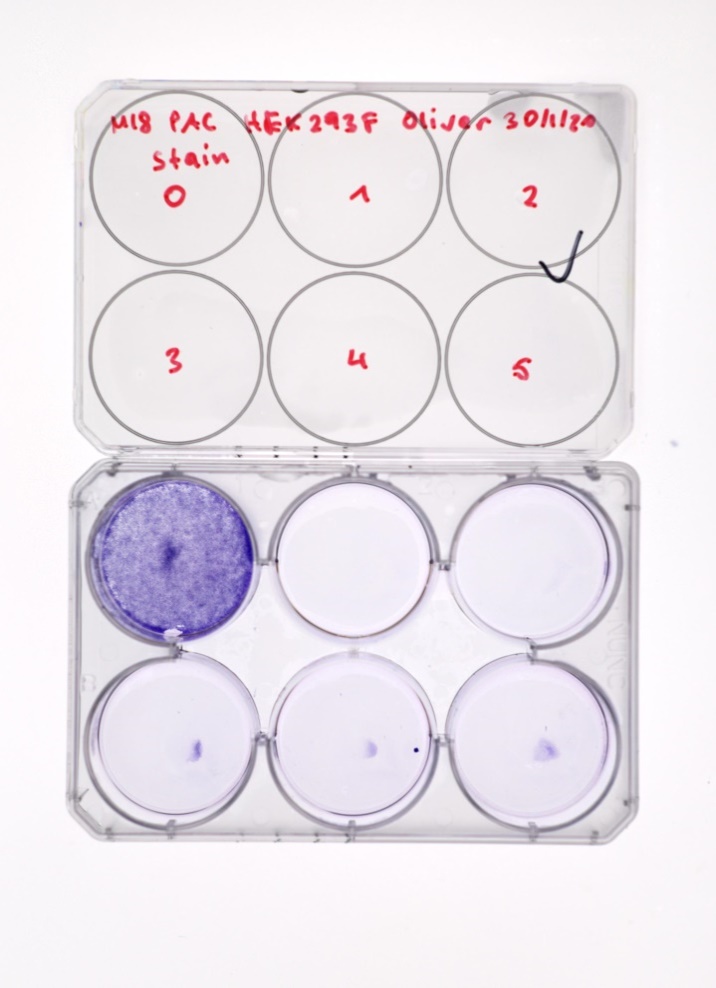 | 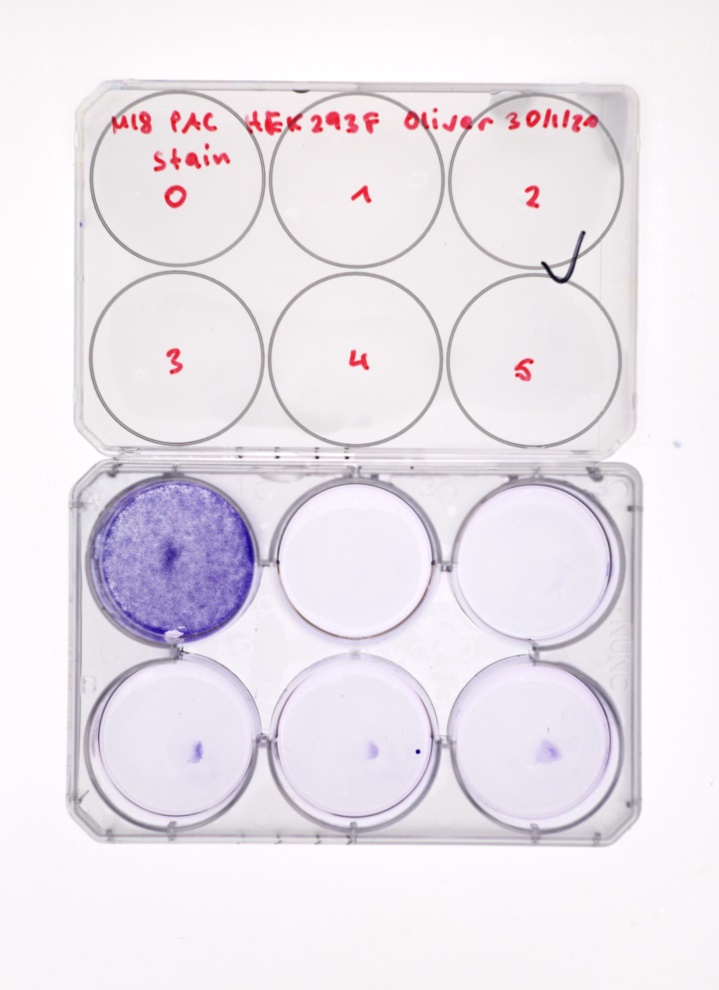 | 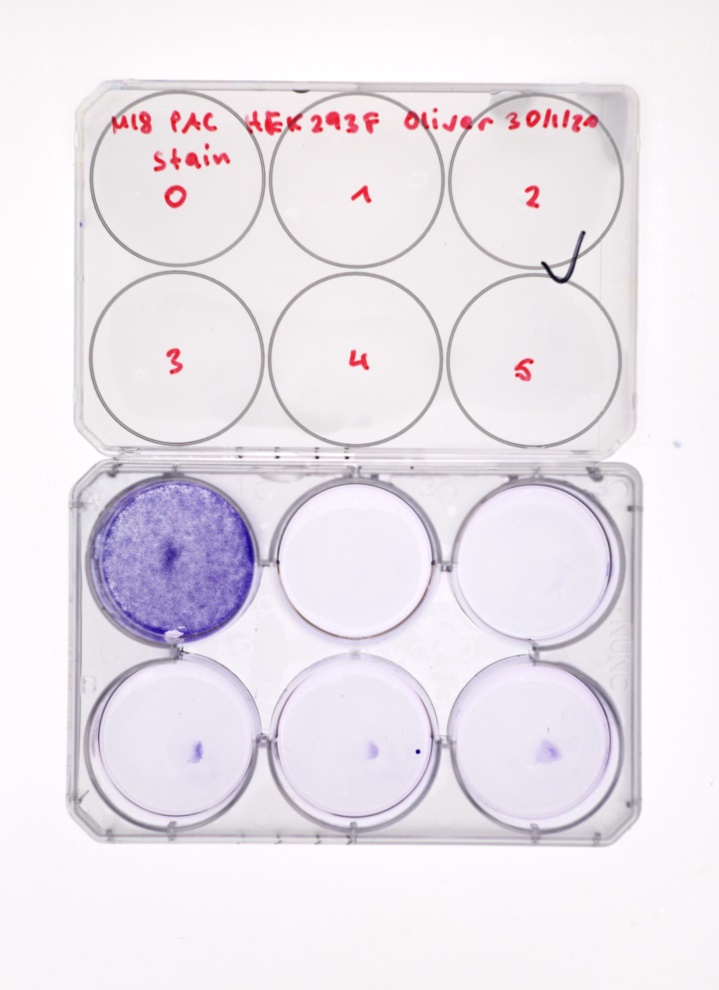 | 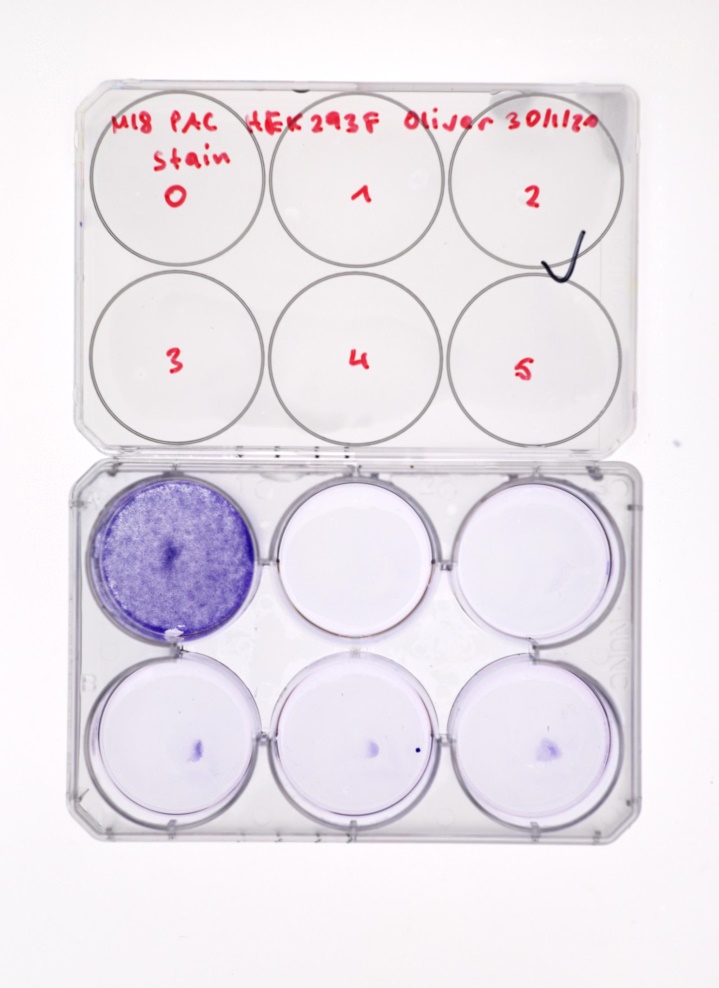 | 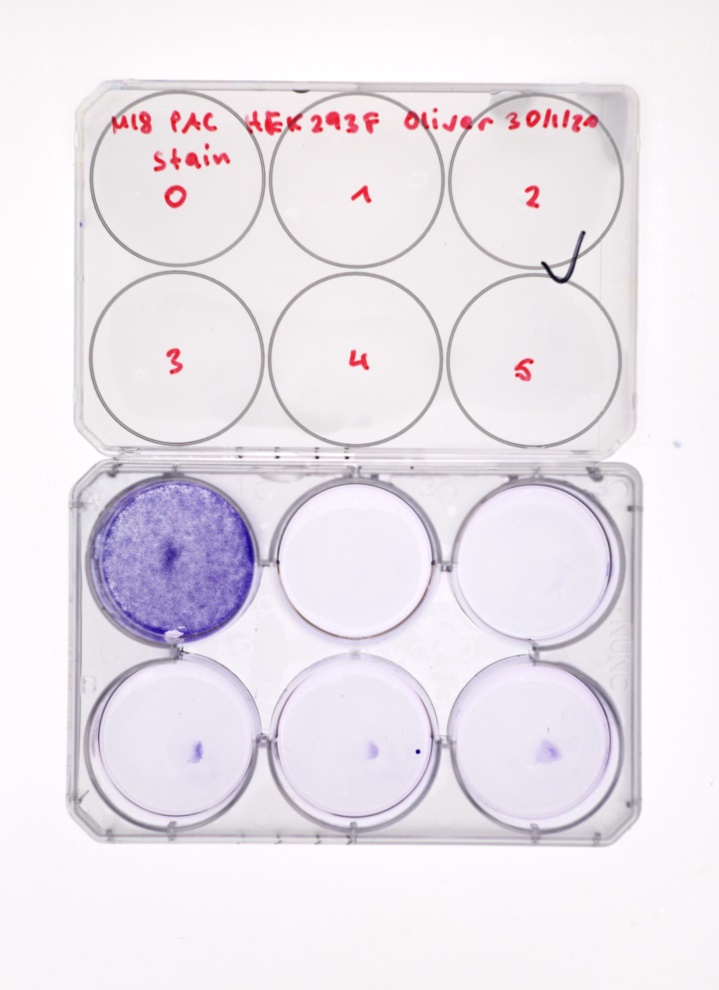 |
| Y30F | 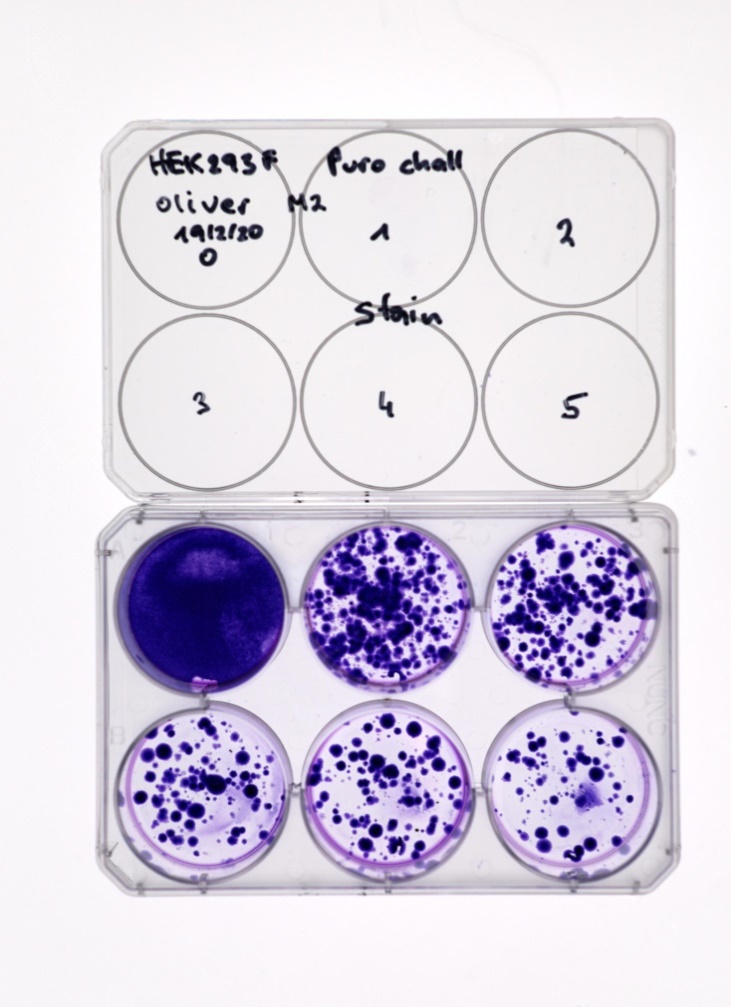 | 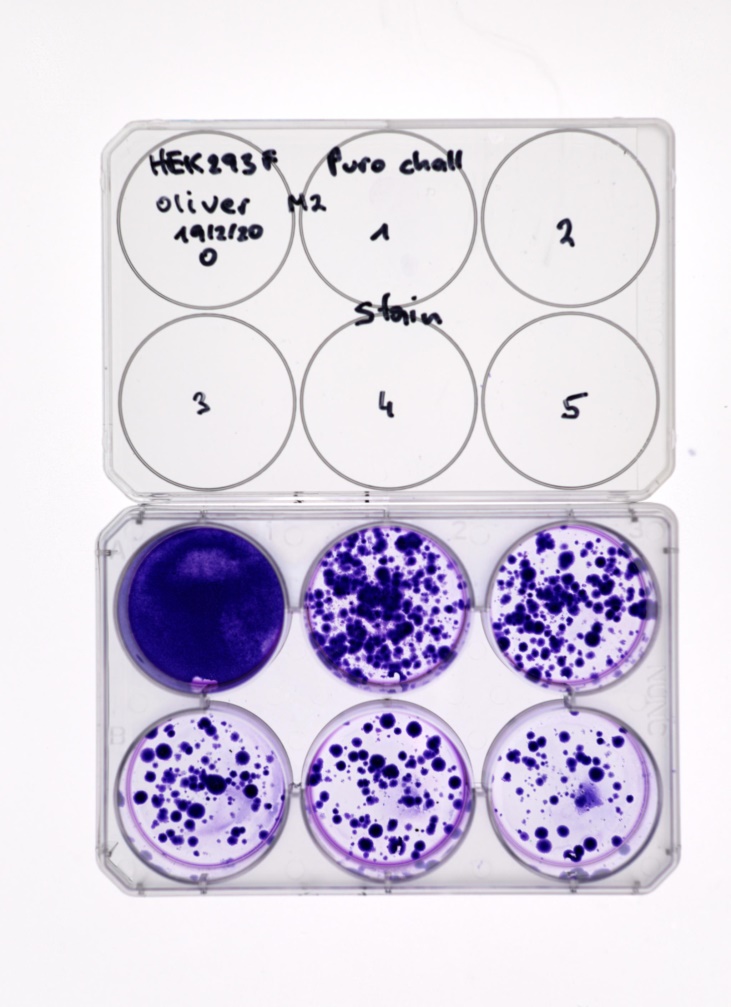 | 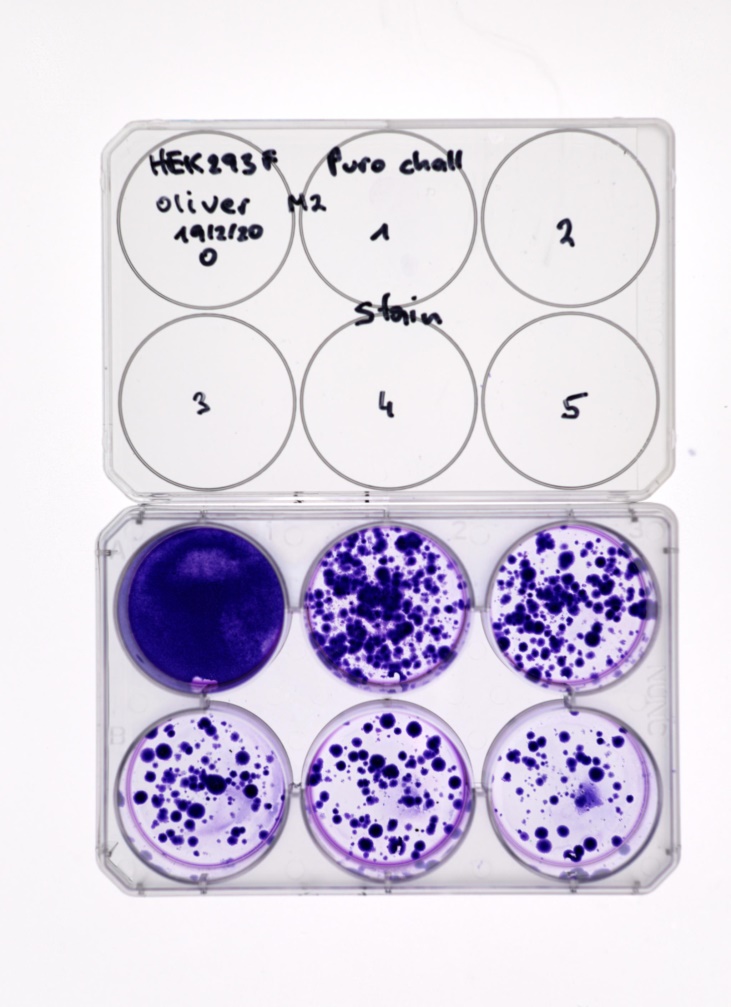 | 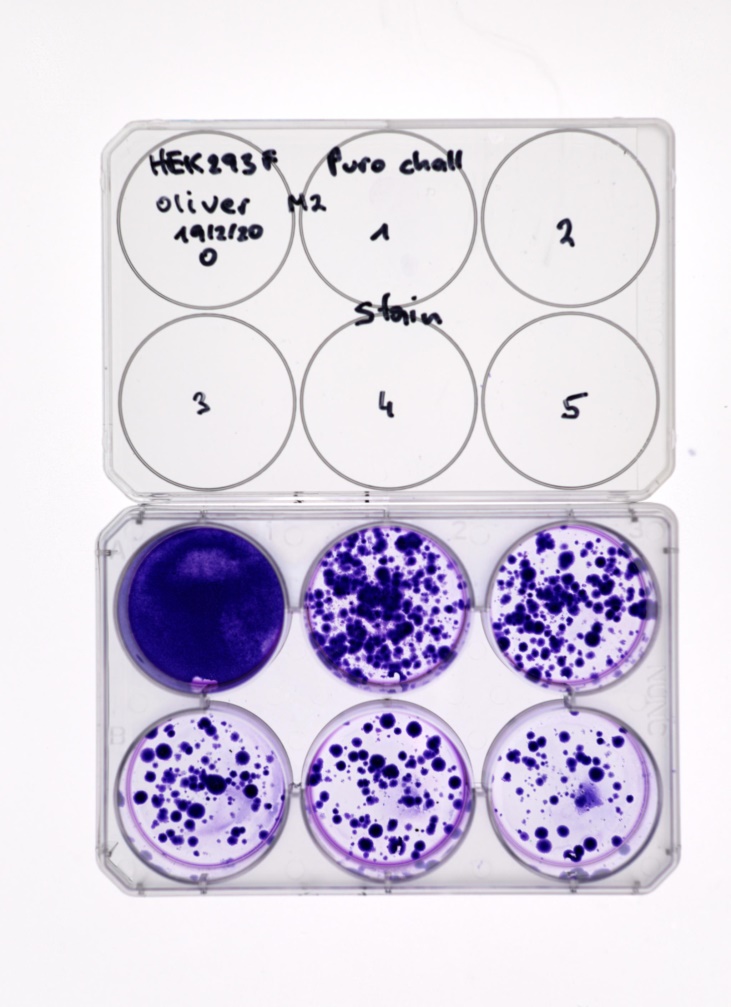 | 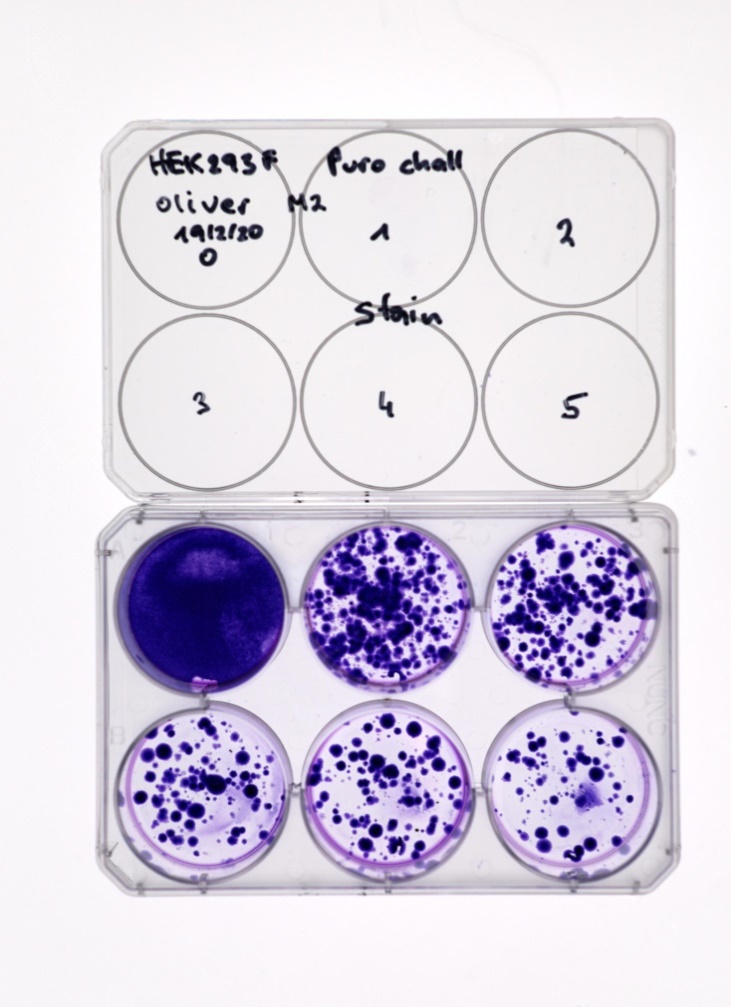 | 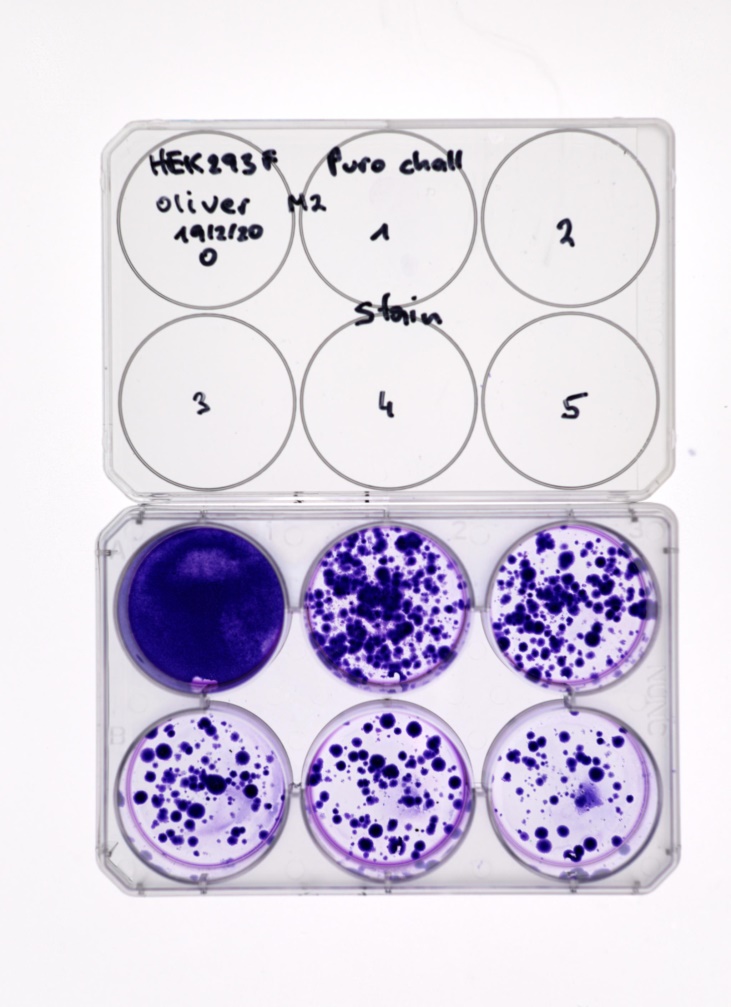 |
| T162V | 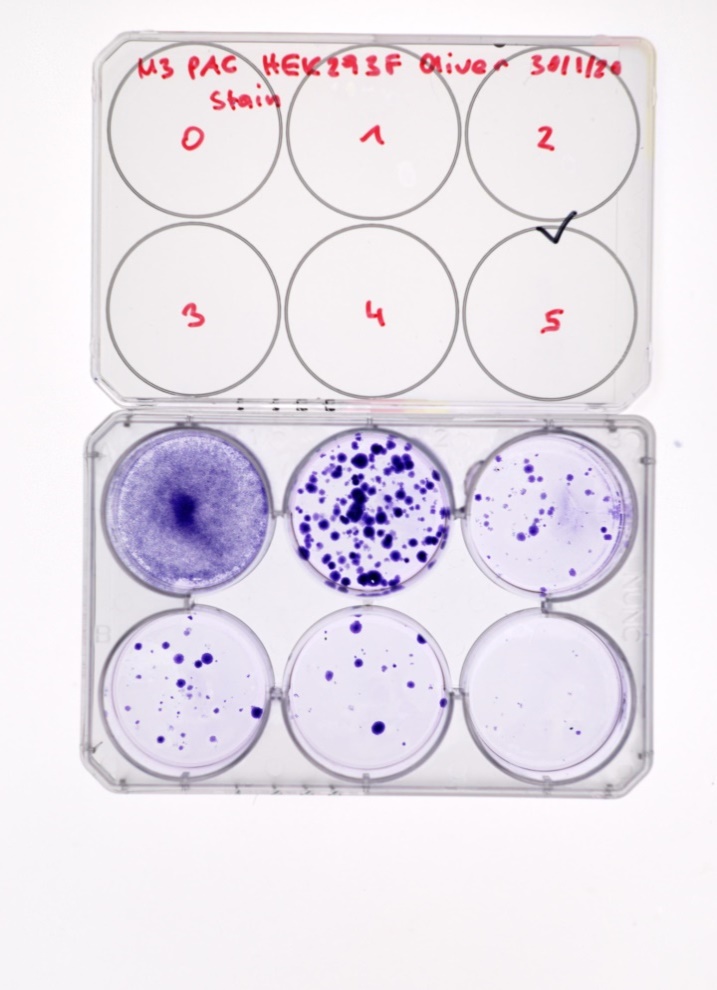 | 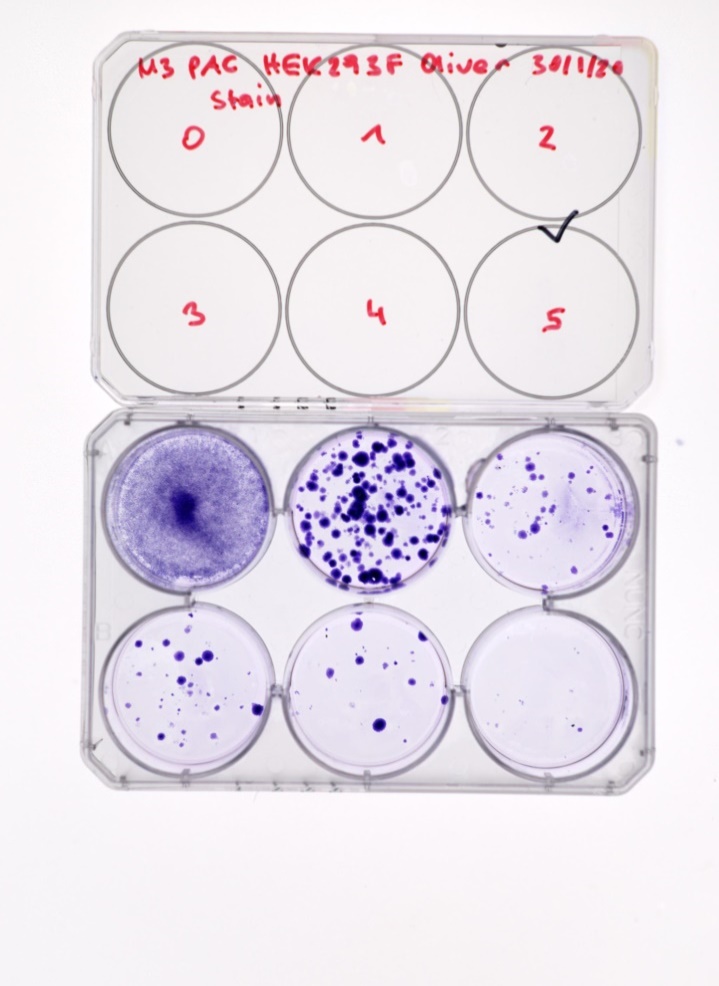 | 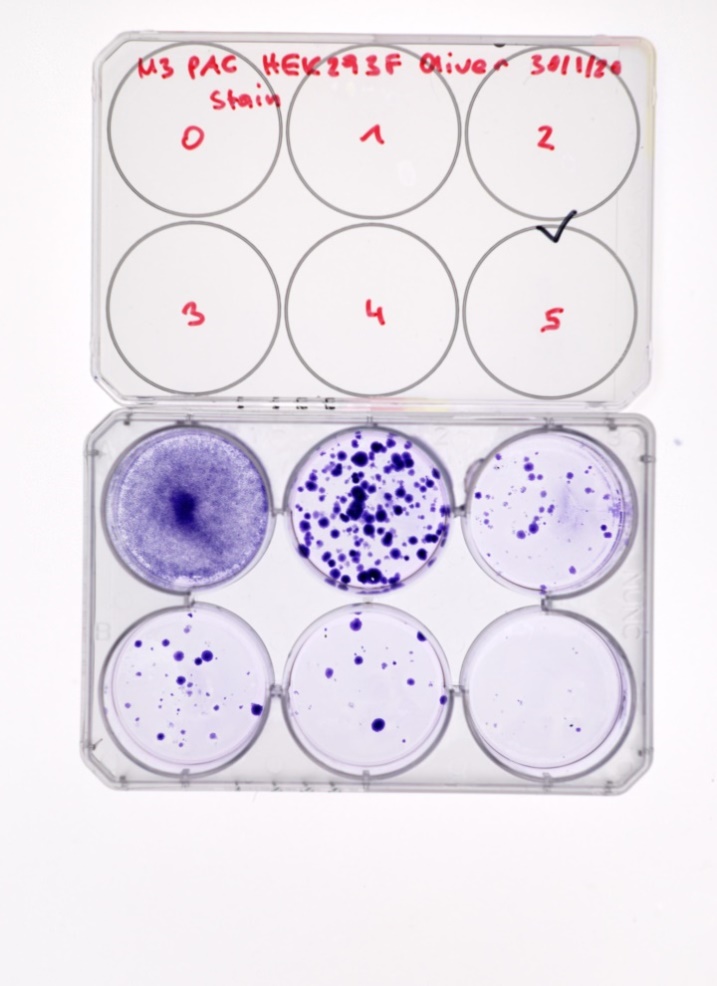 | 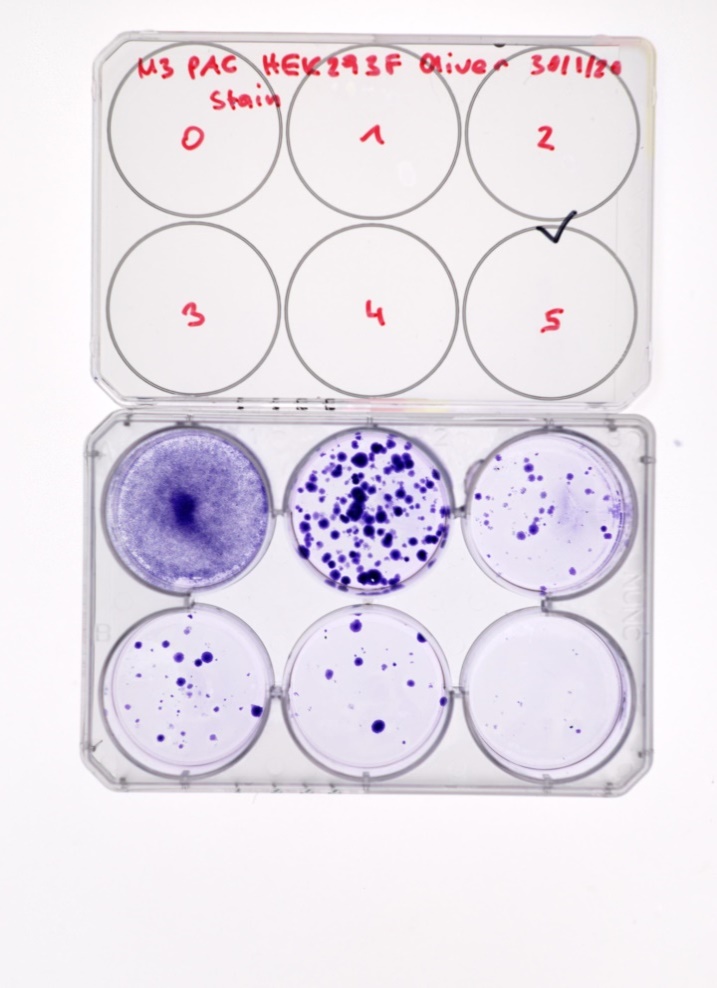 | 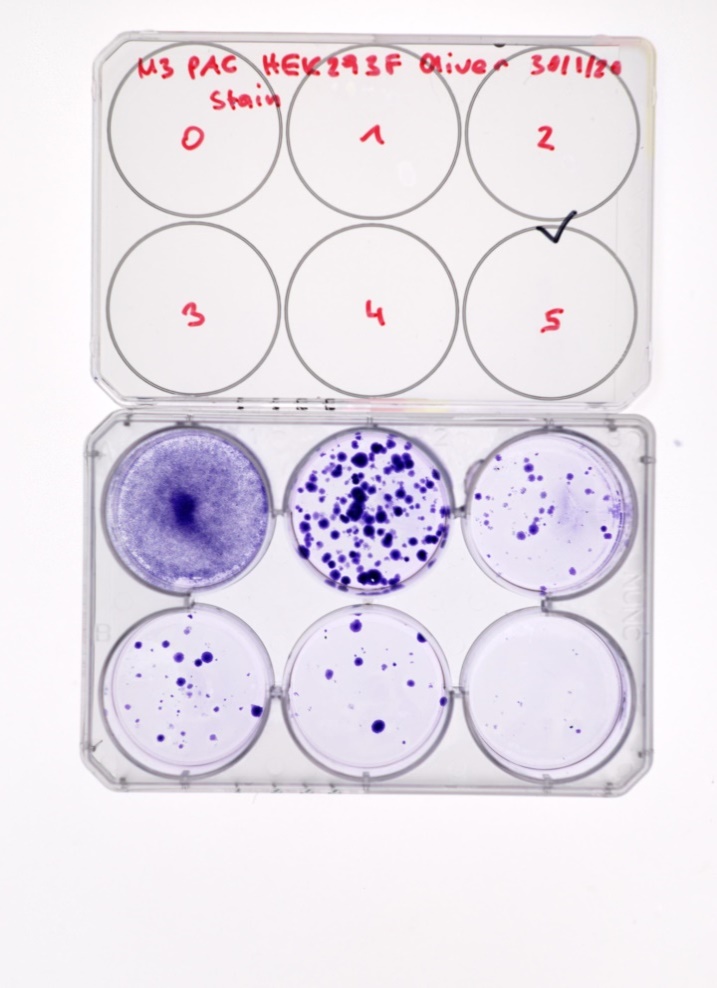 | 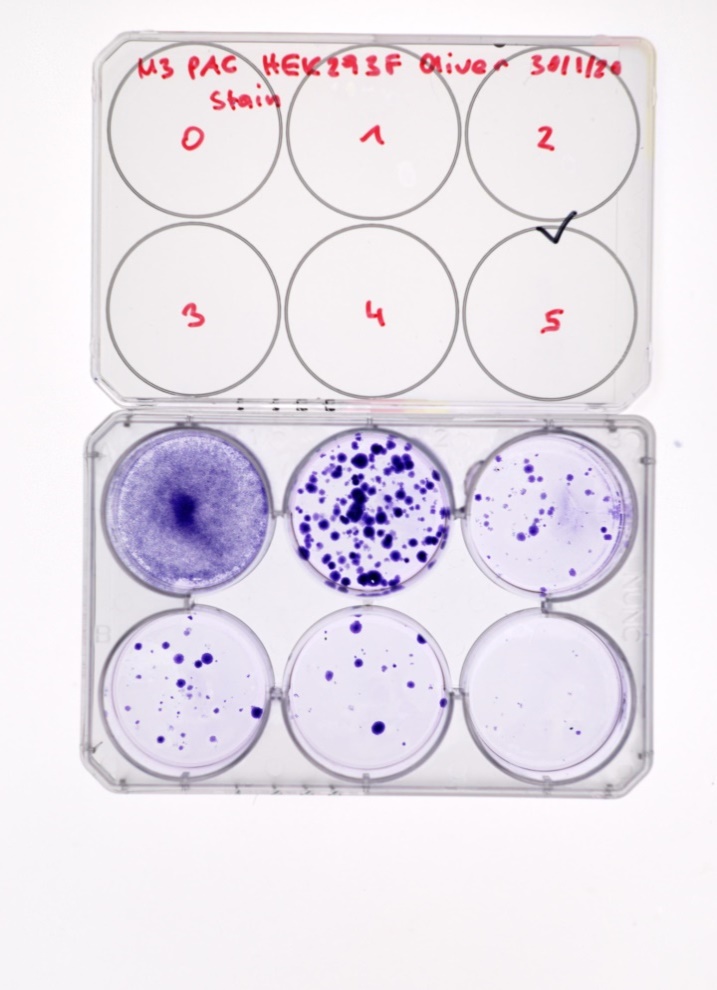 |
| T162A | 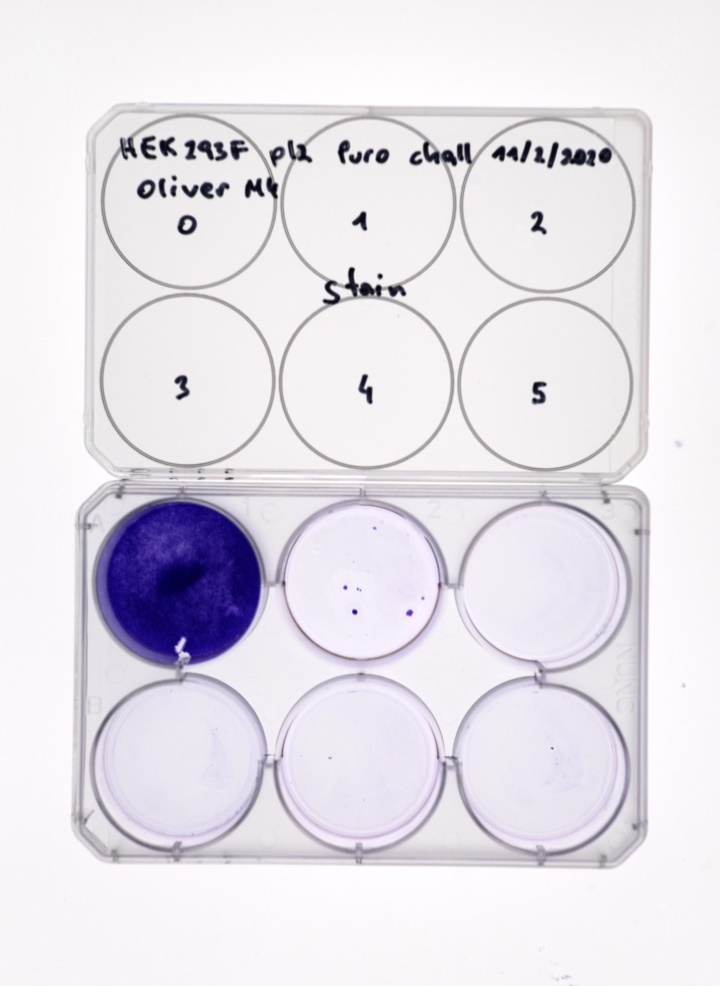 | 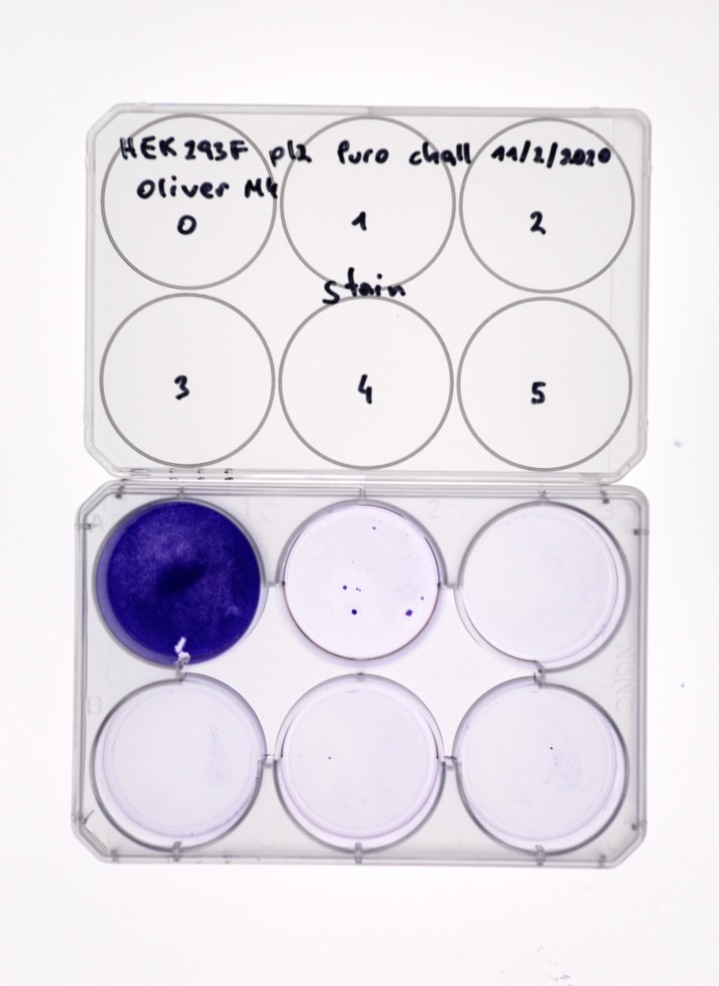 | 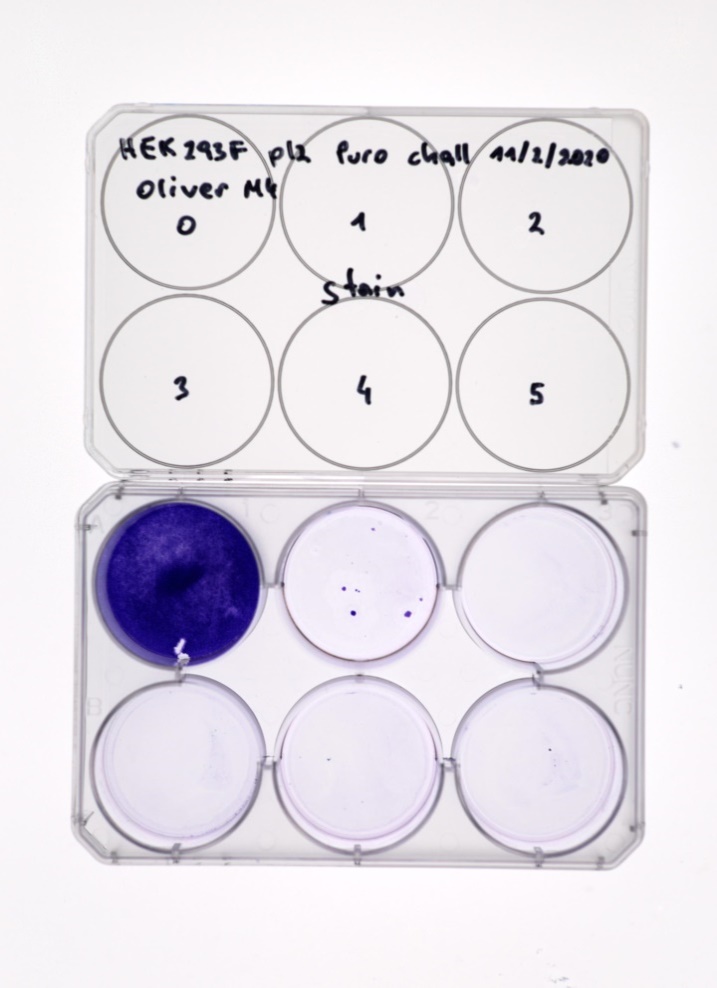 | 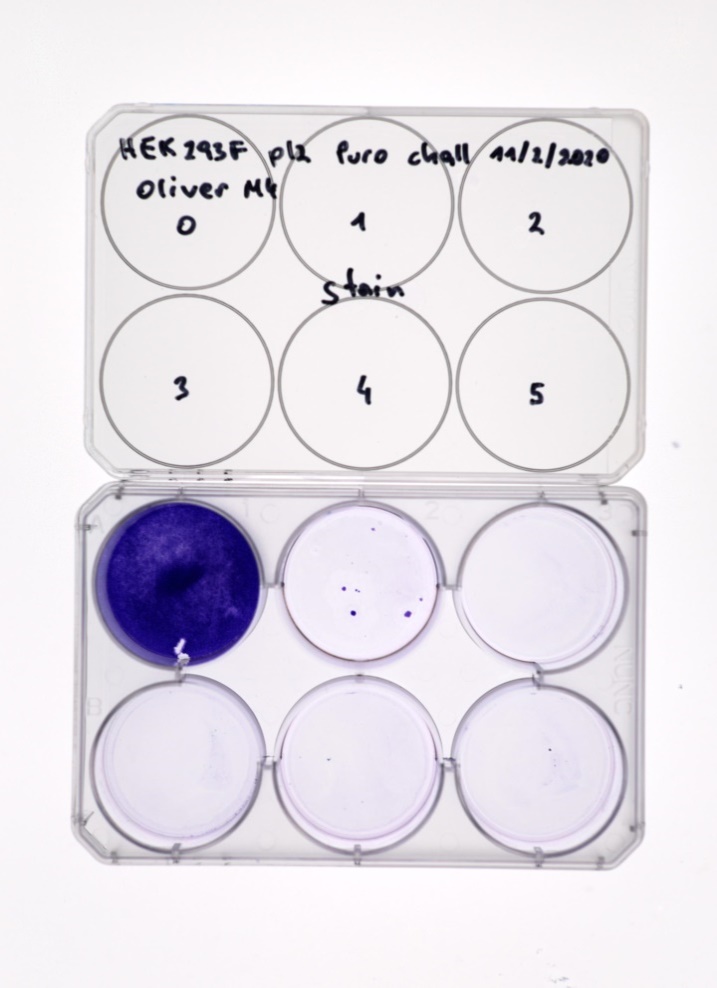 | 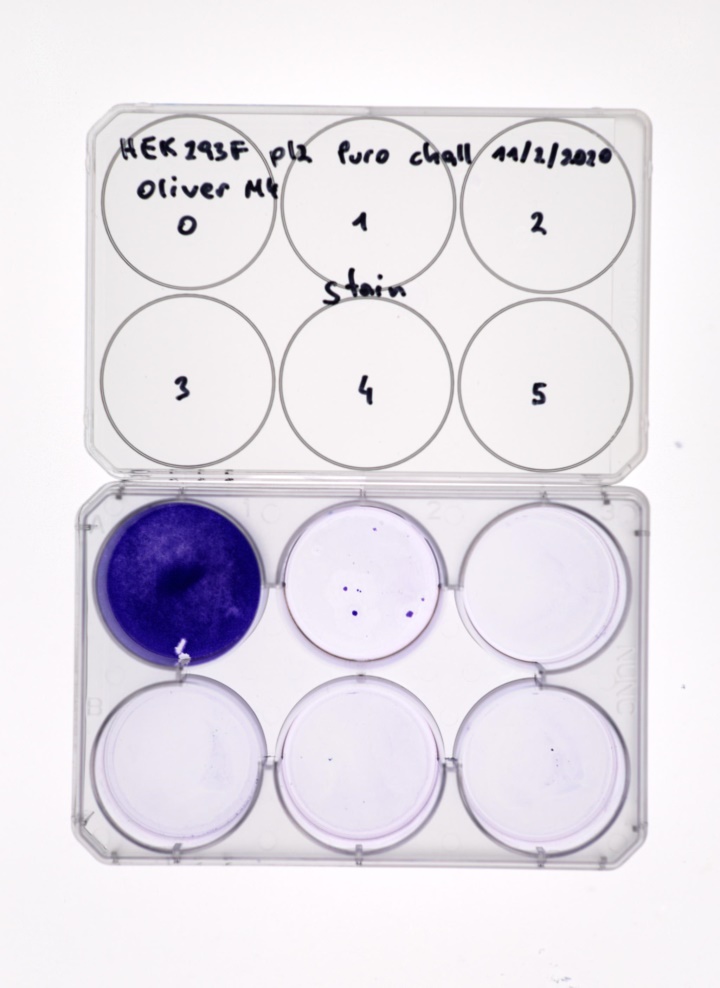 | 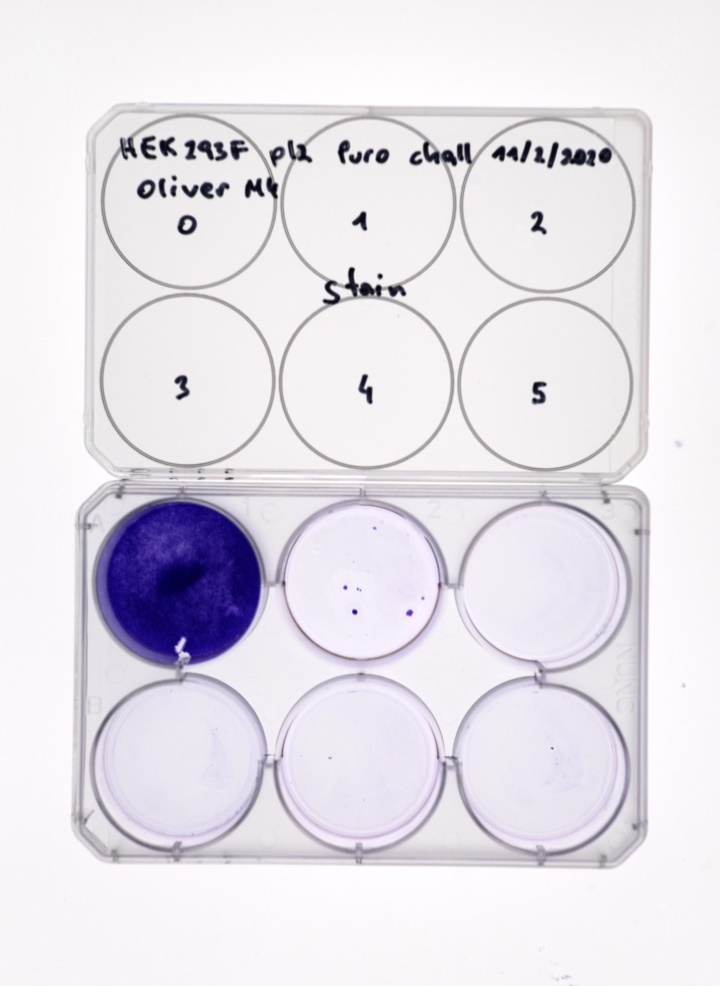 |
| N167D | 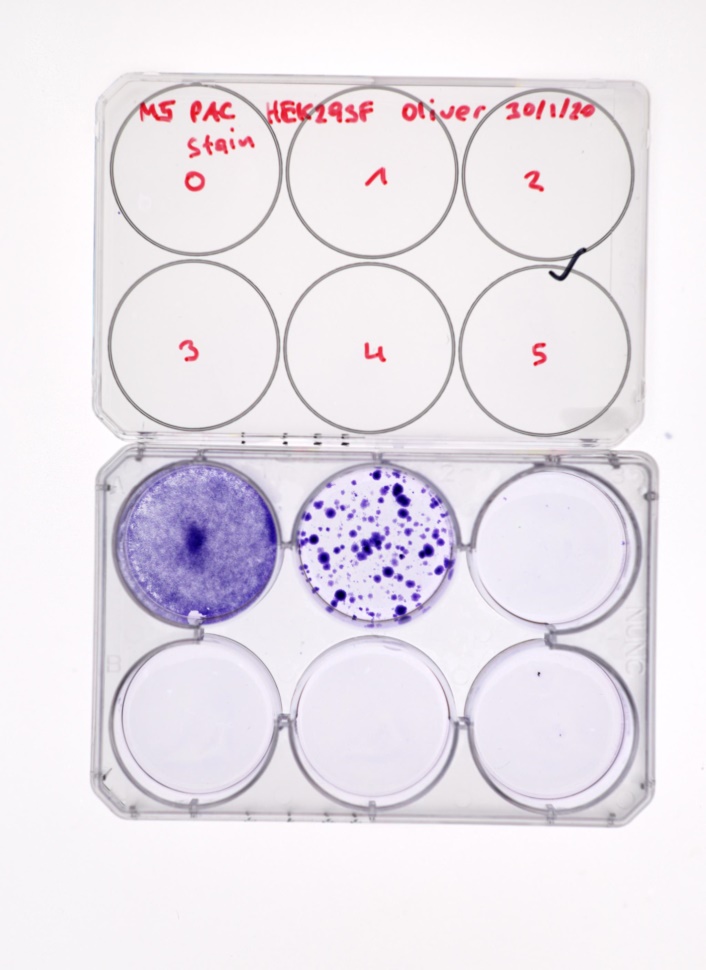 | 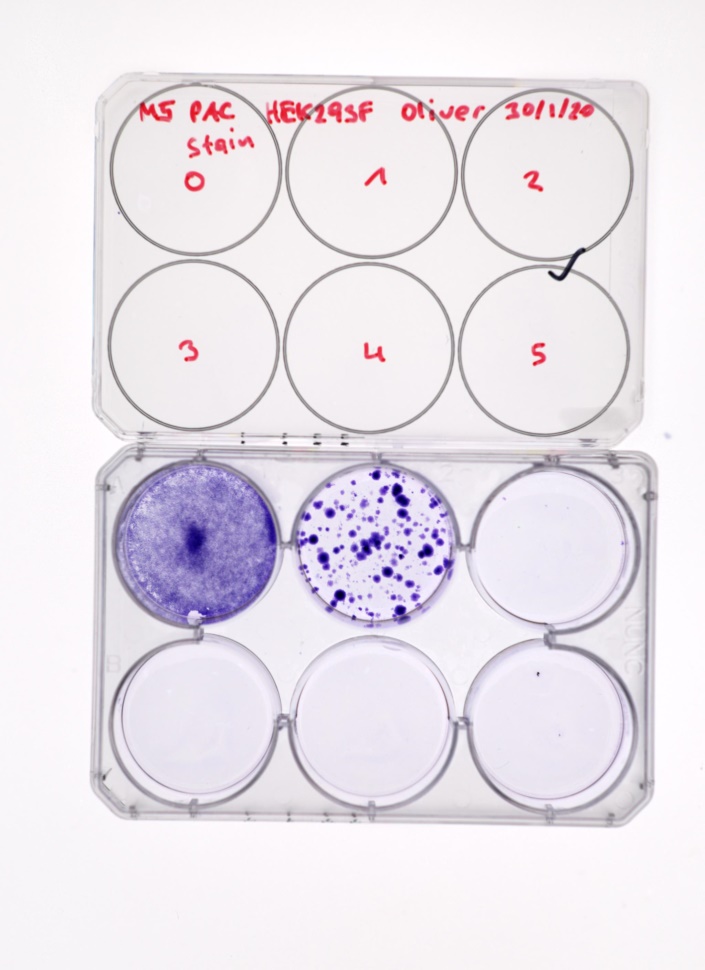 | 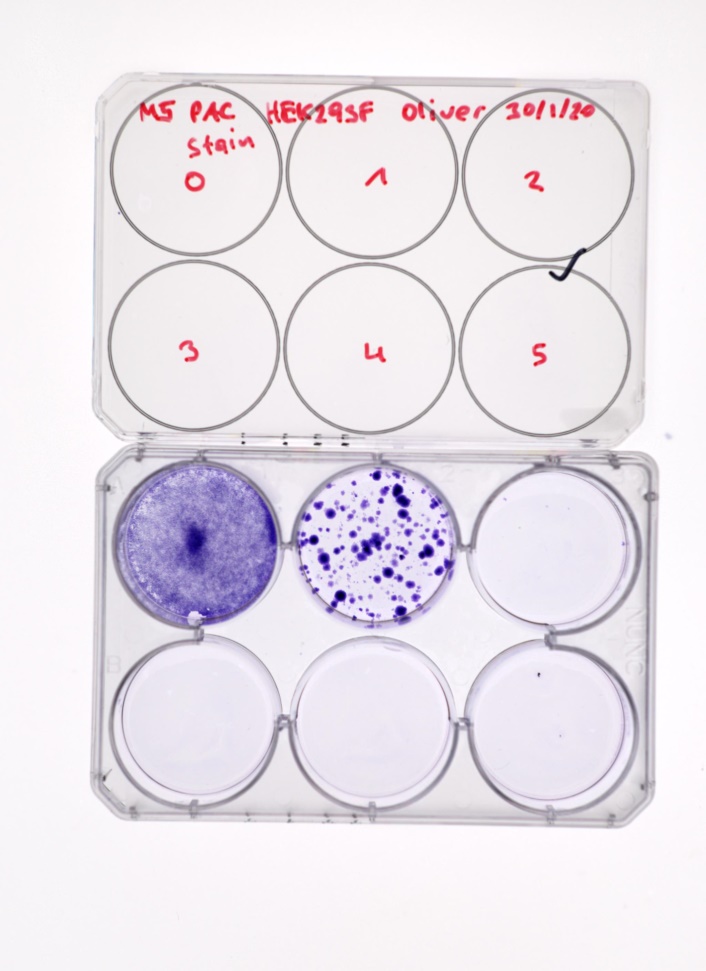 | 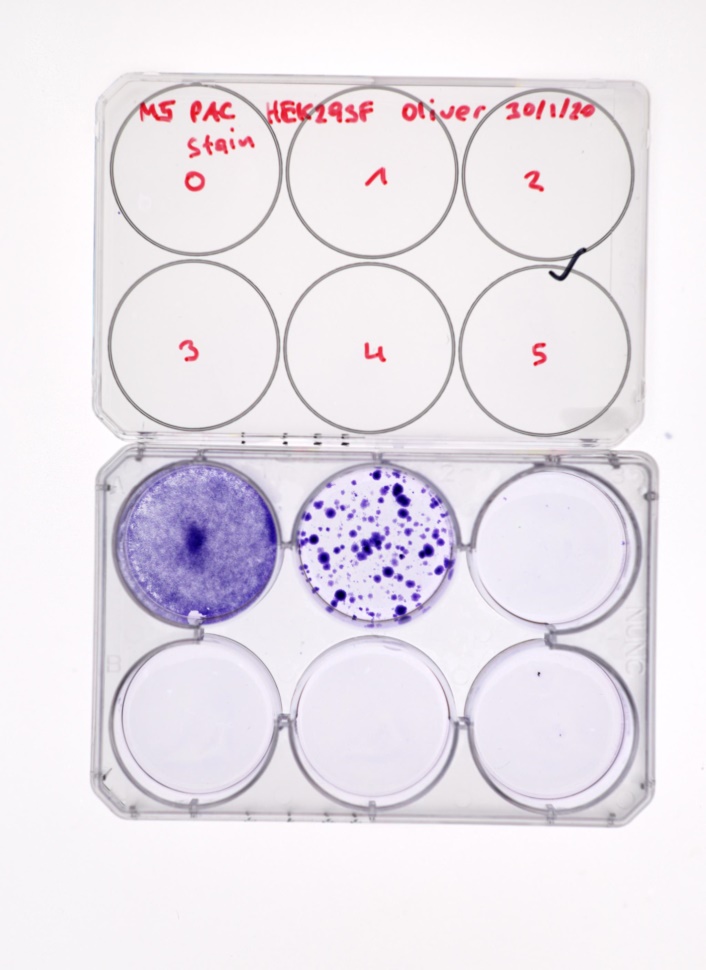 | 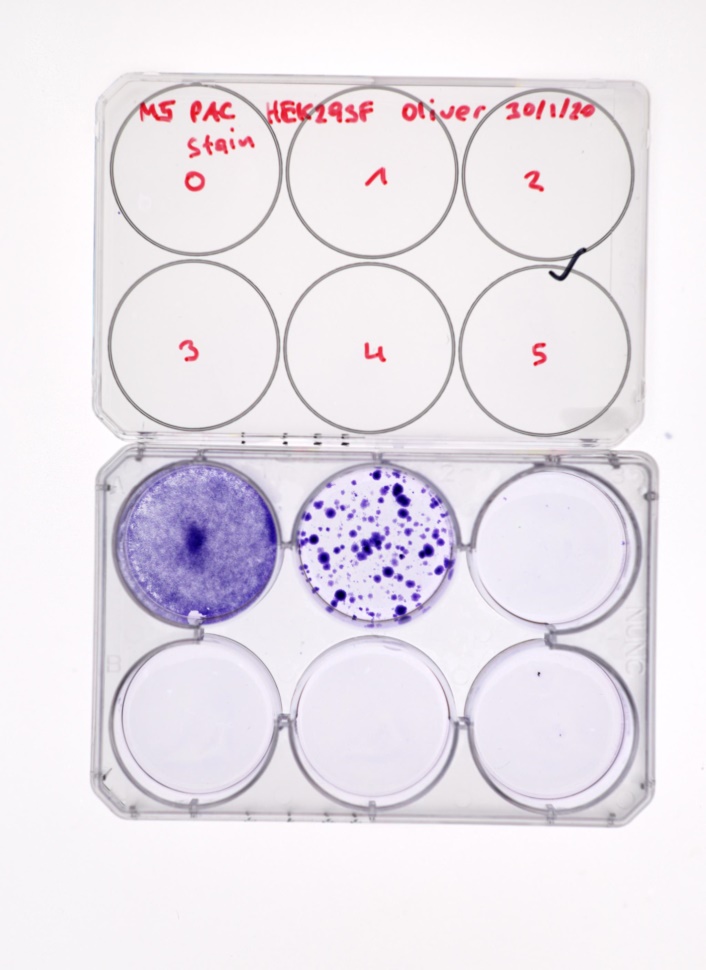 | 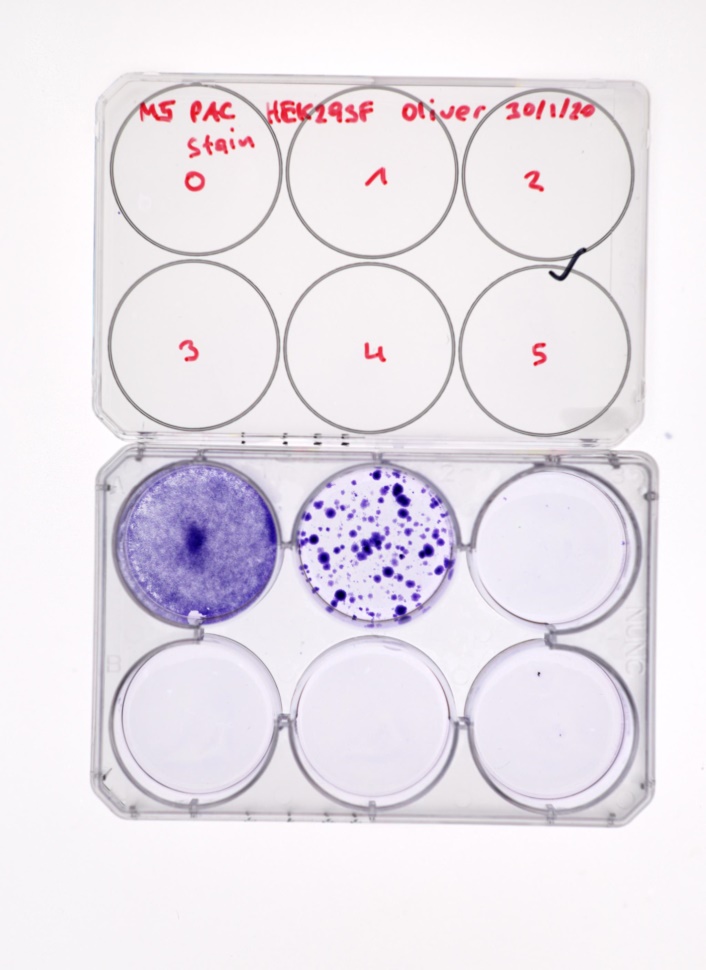 |
| N167Q | 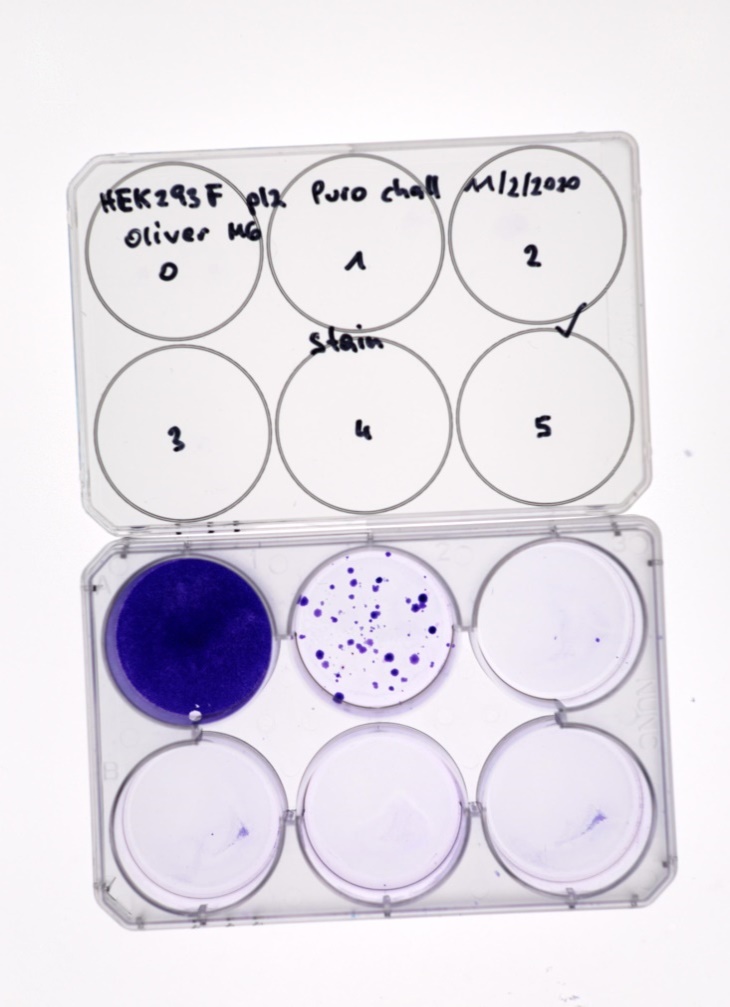 | 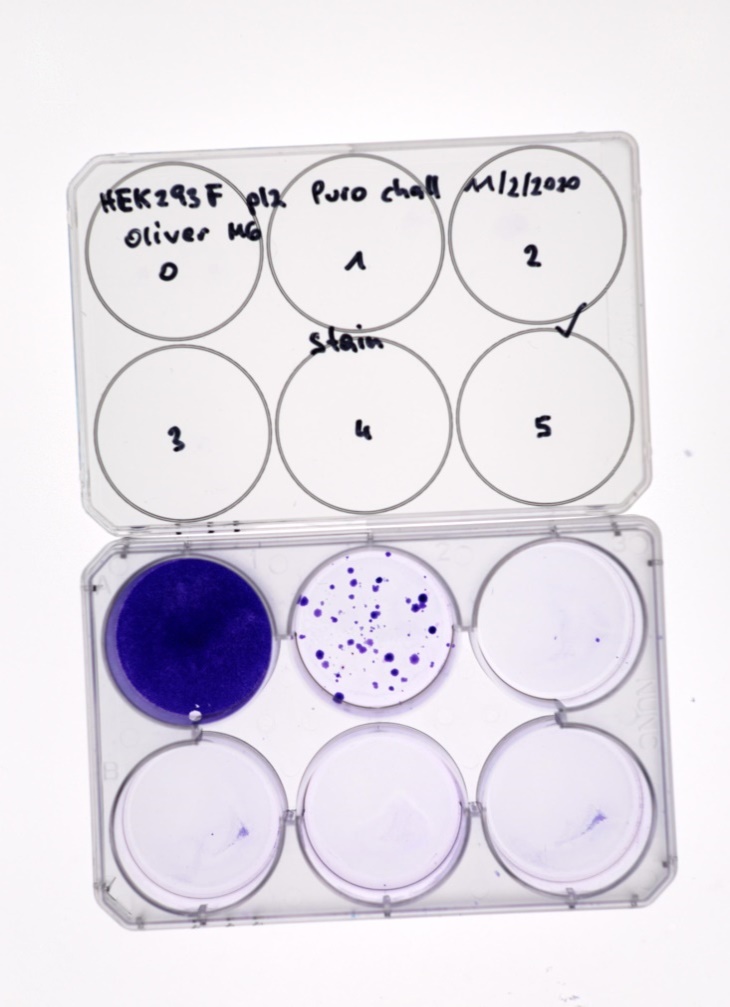 | 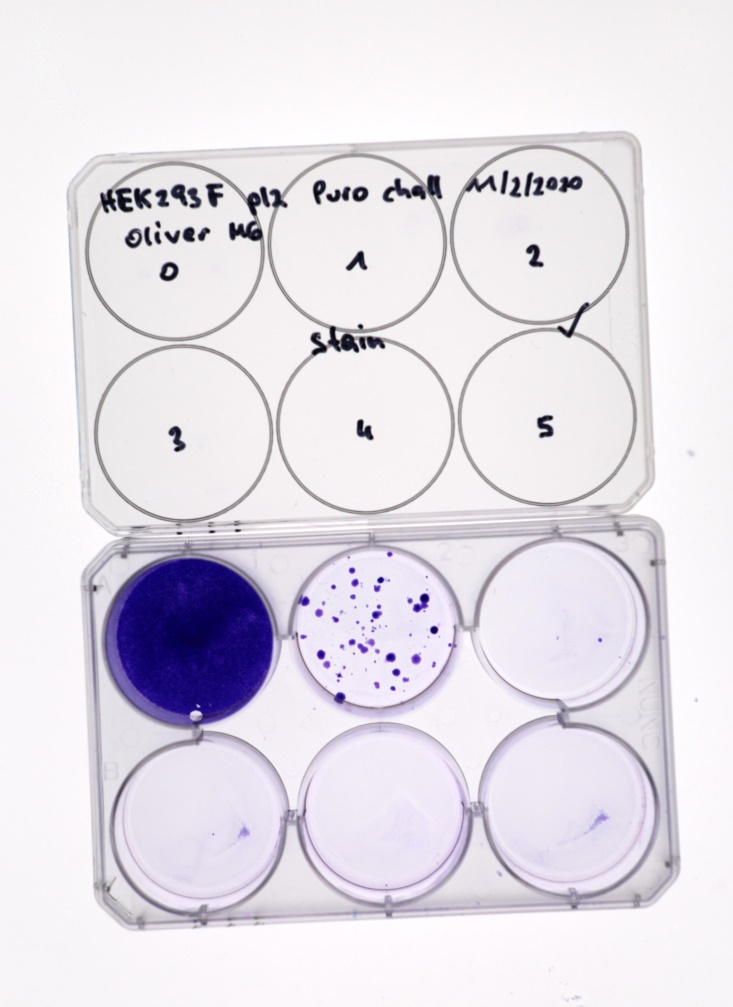 | 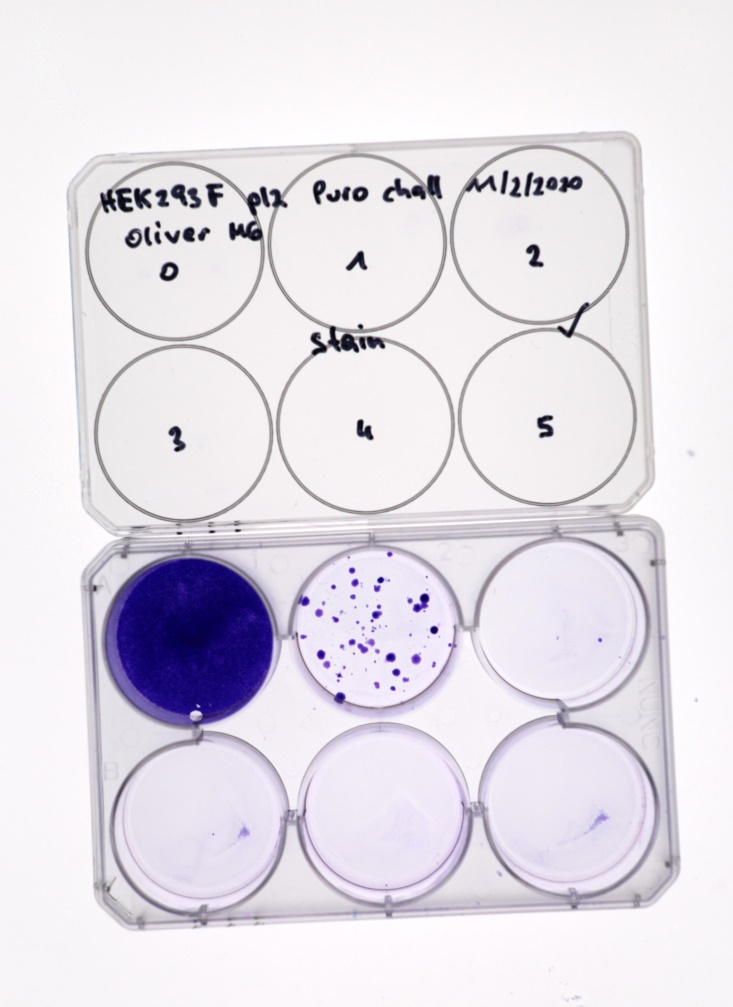 | 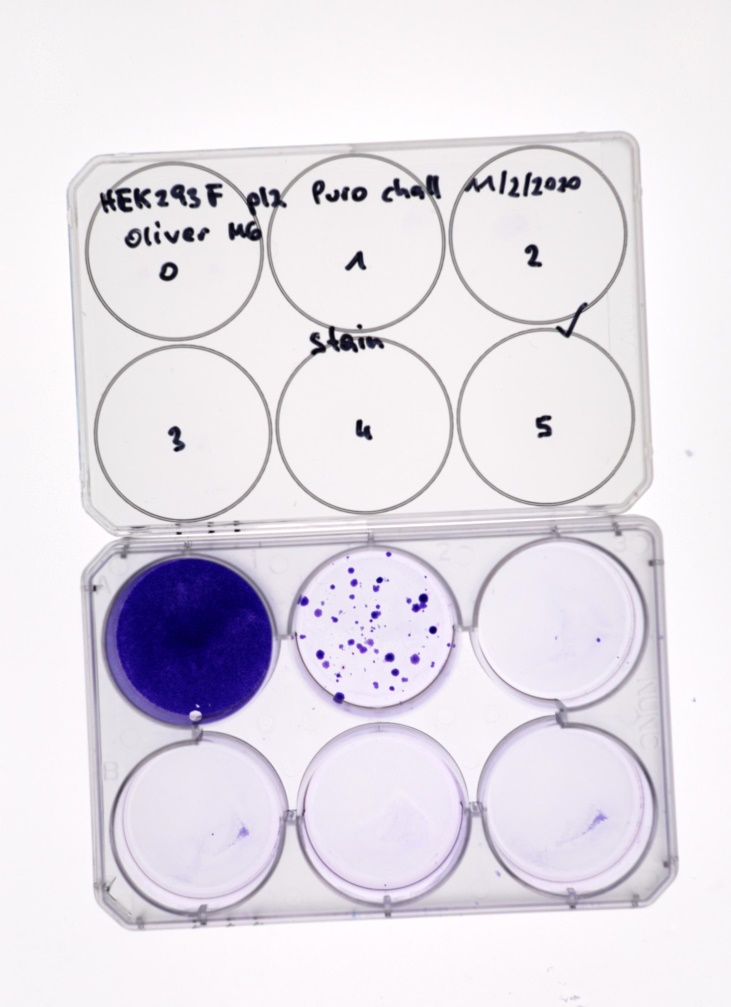 | 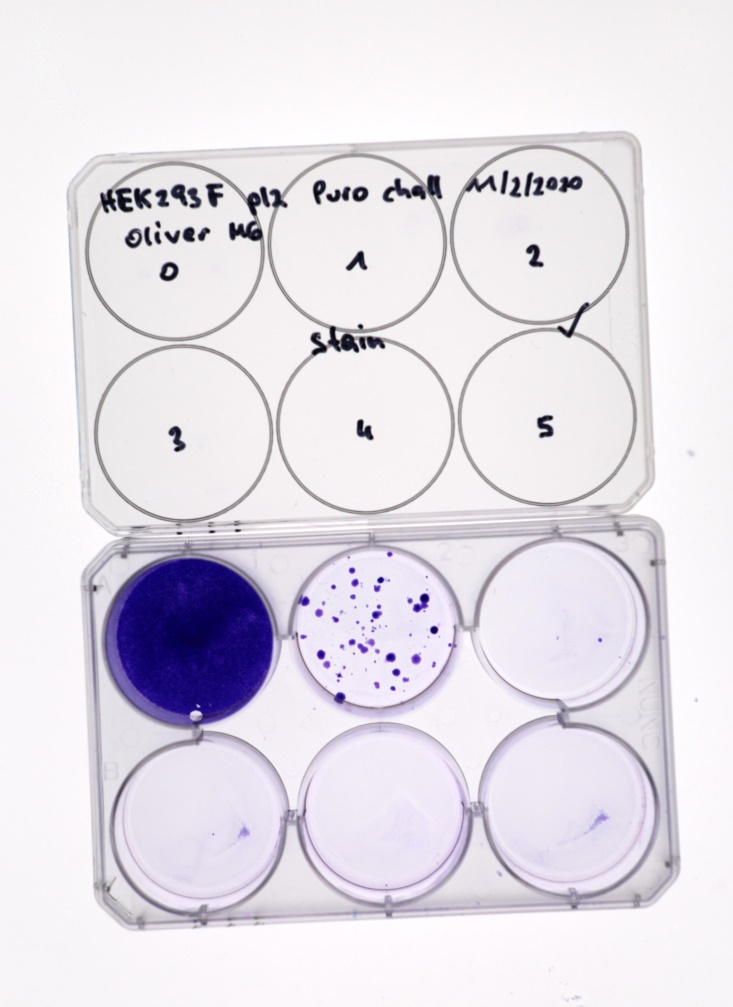 |
| N167A | 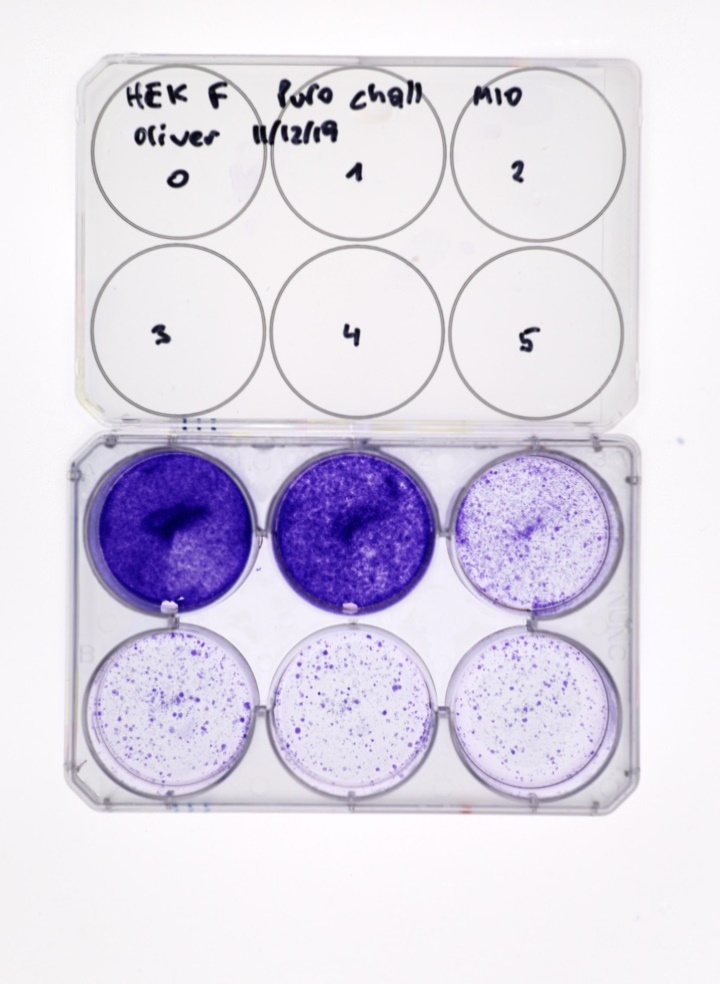 | 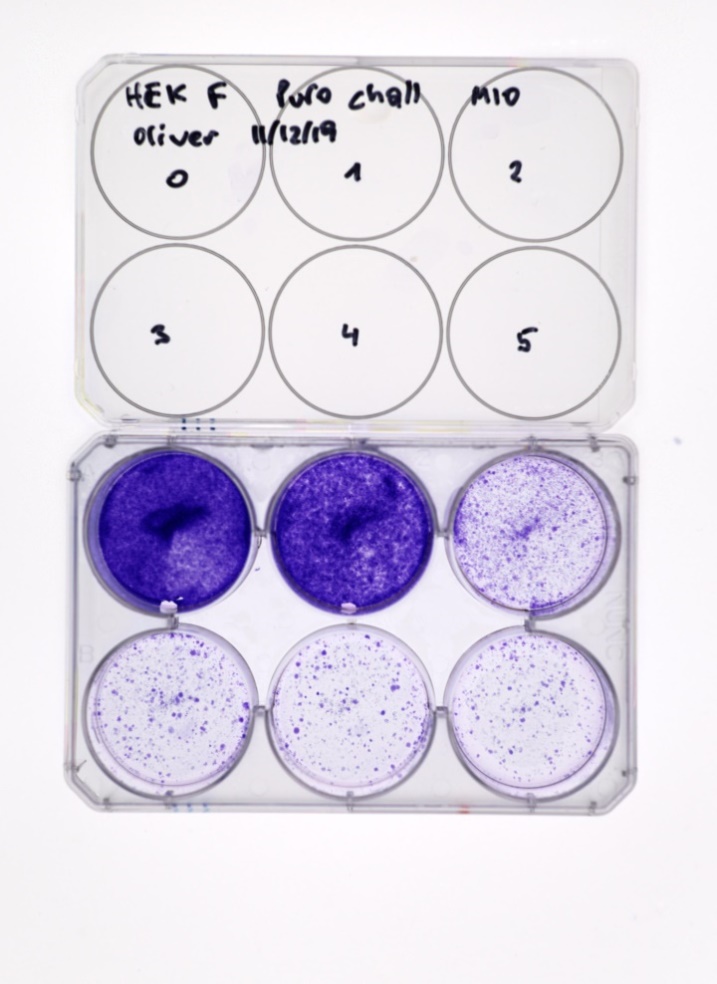 | 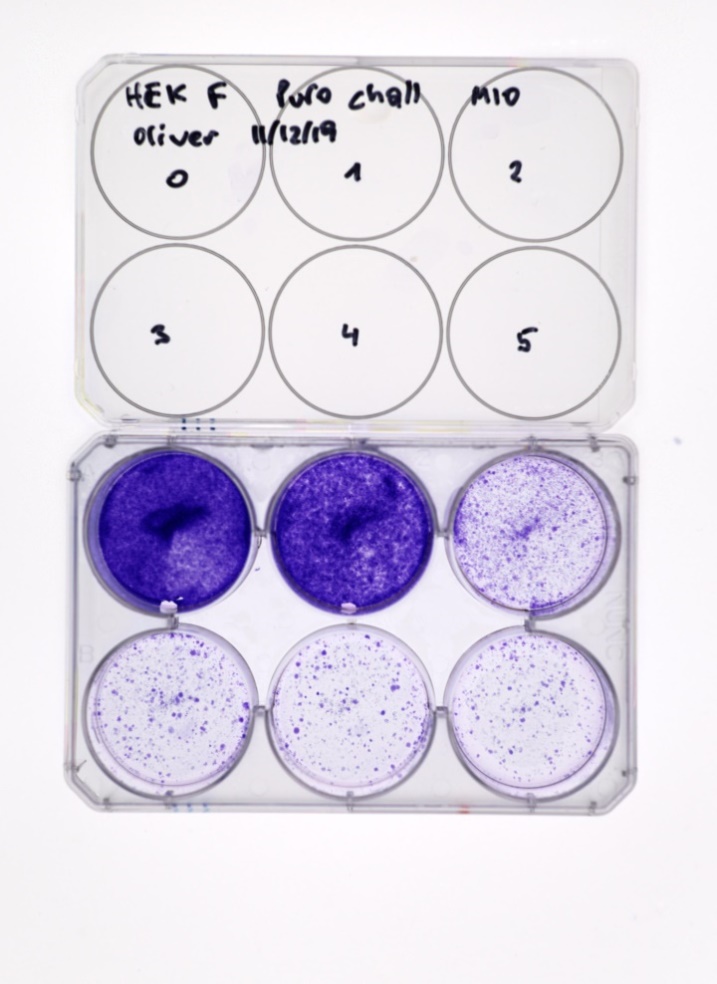 | 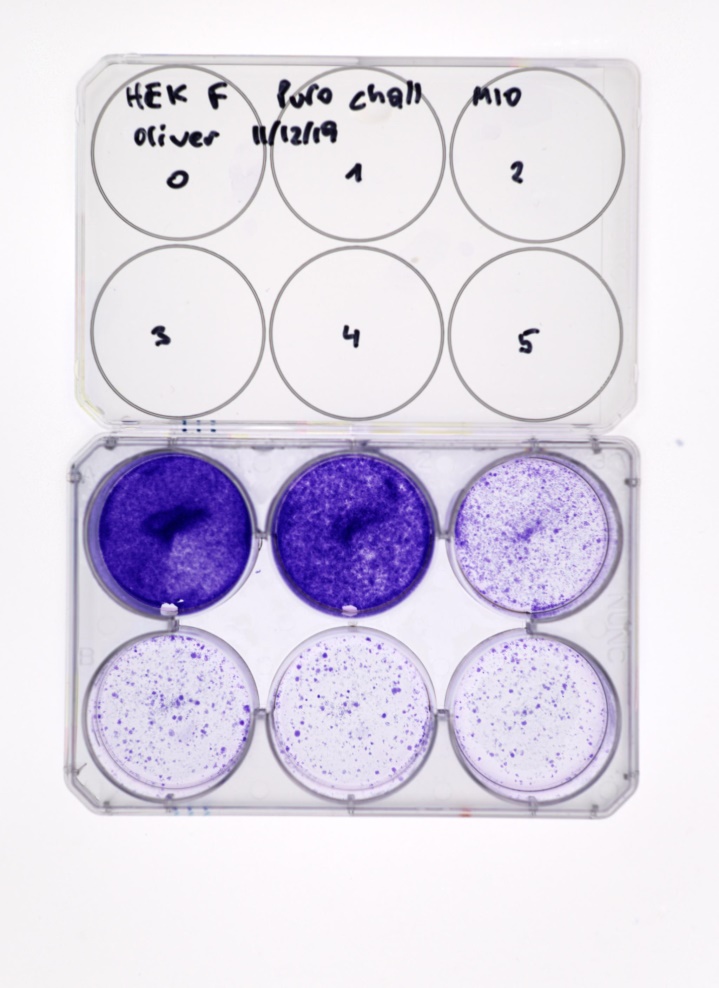 | 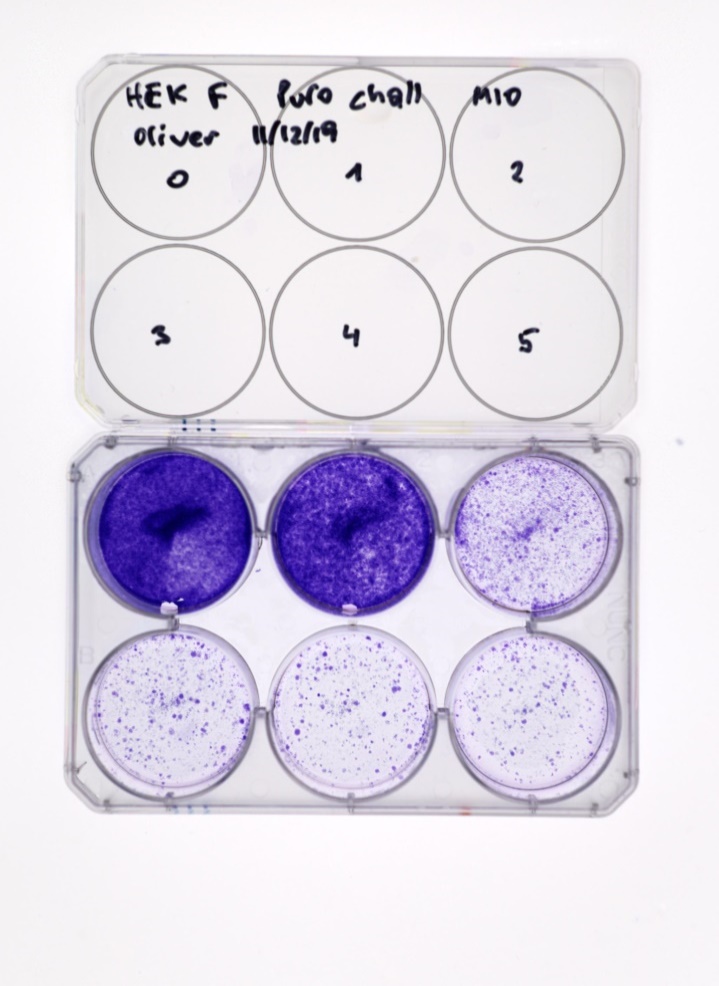 | 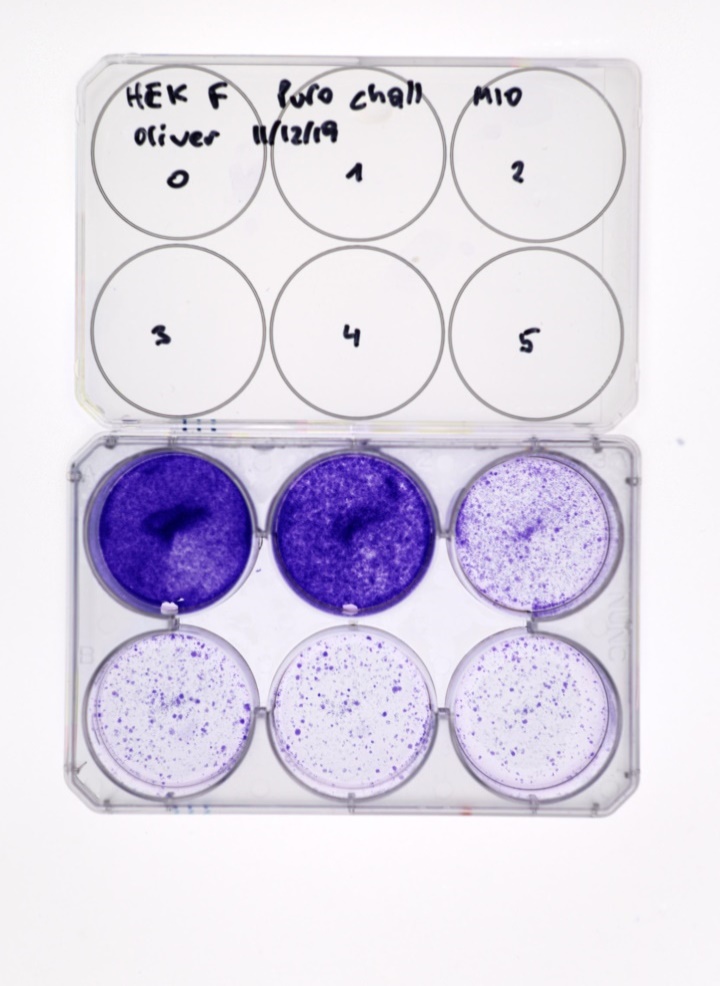 |
| A26S | 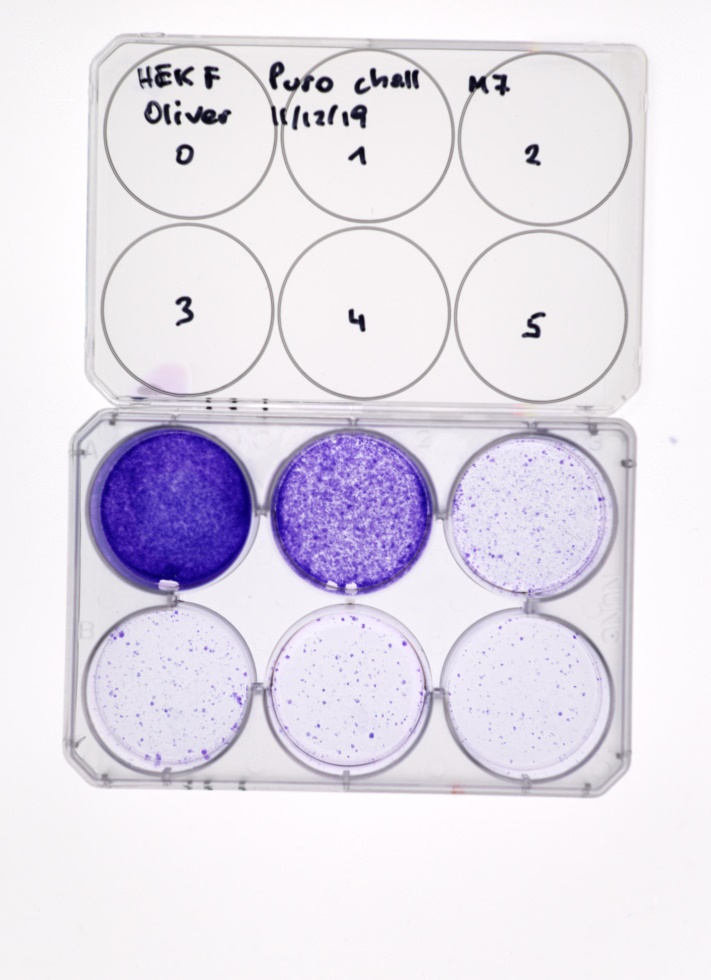 | 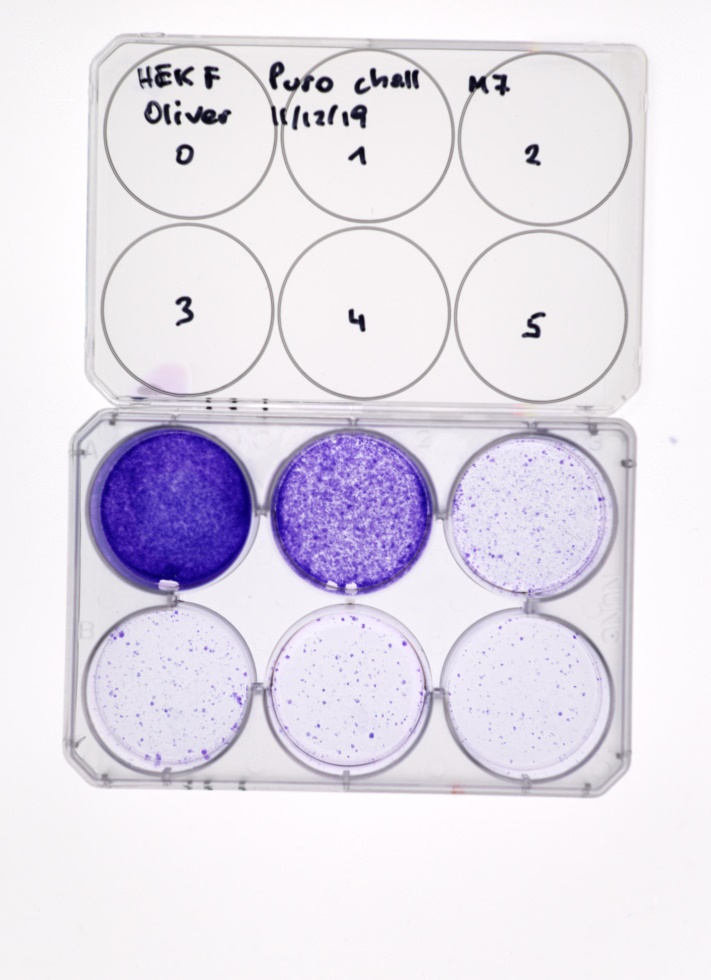 | 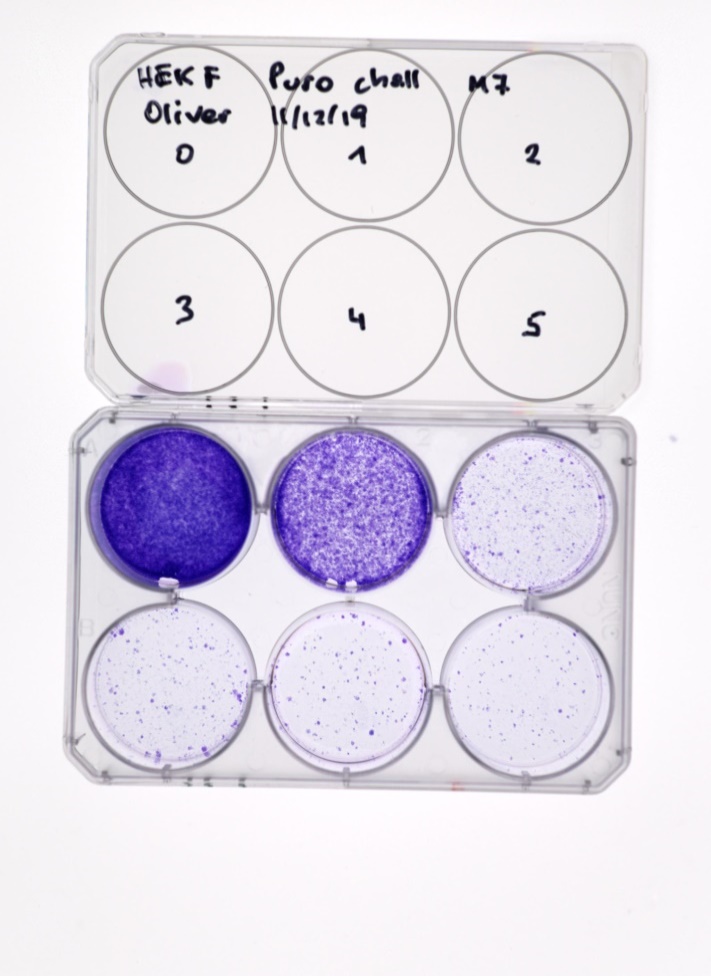 | 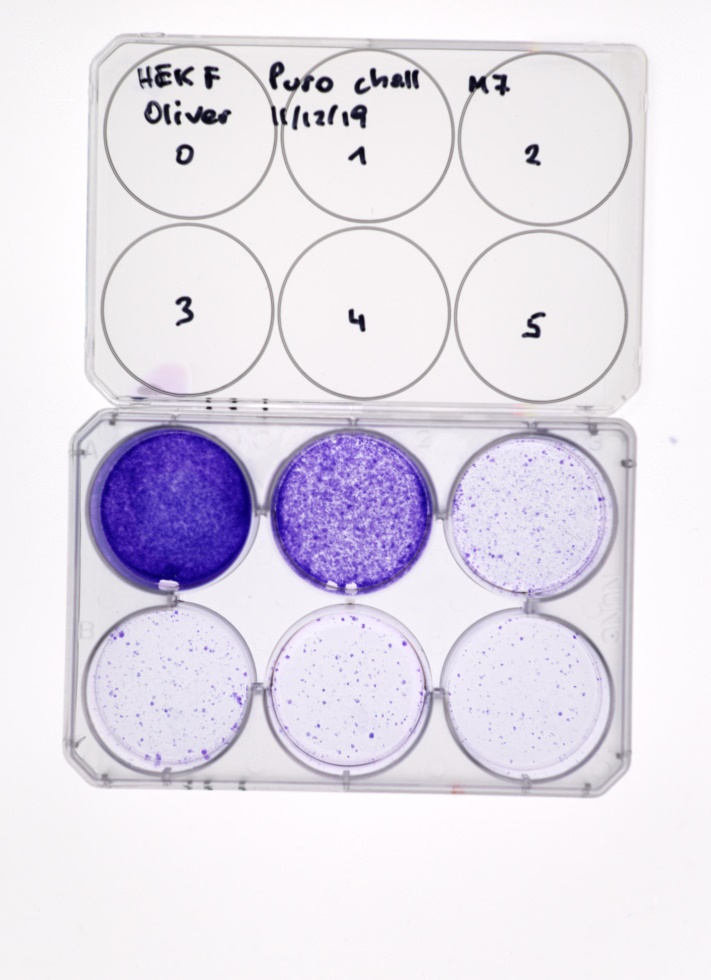 | 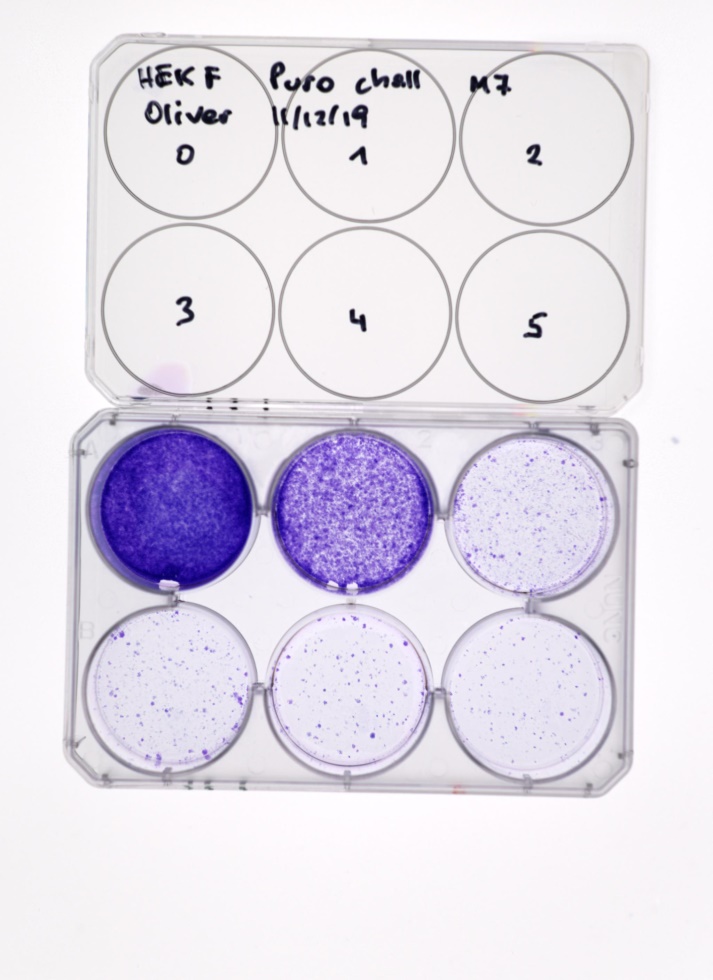 | 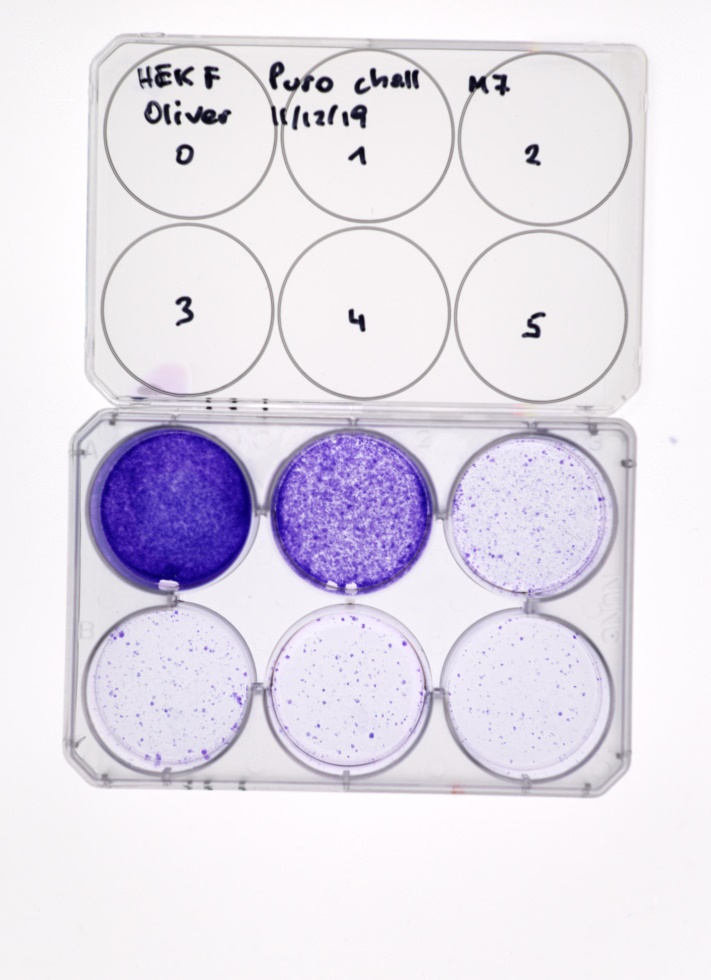 |
| T77S | 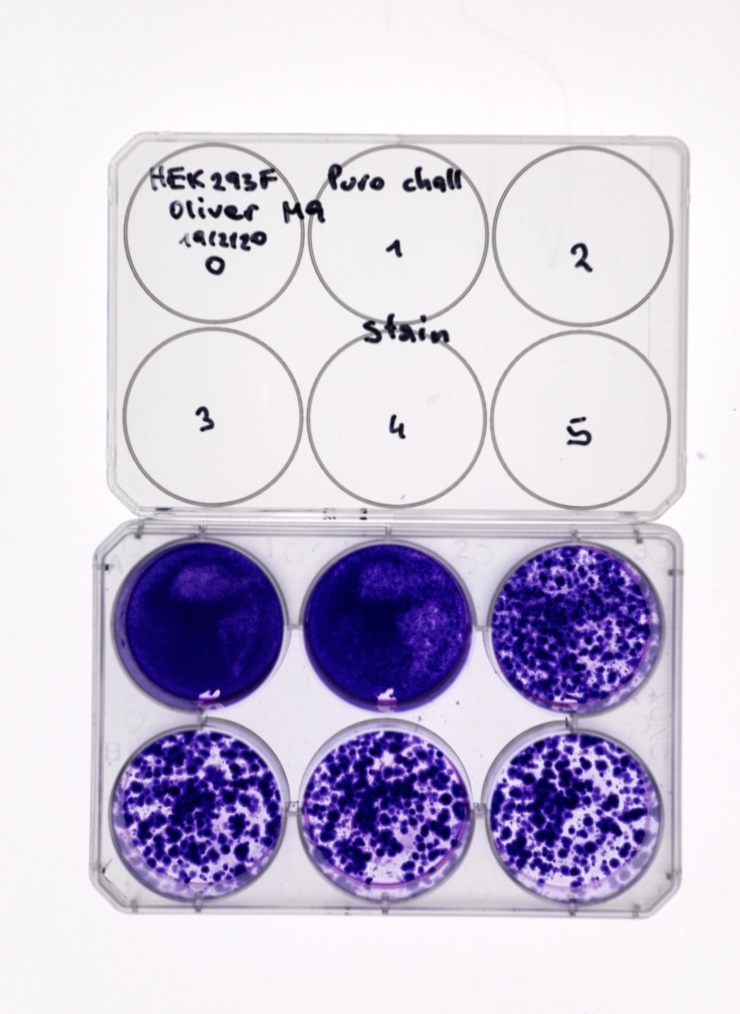 | 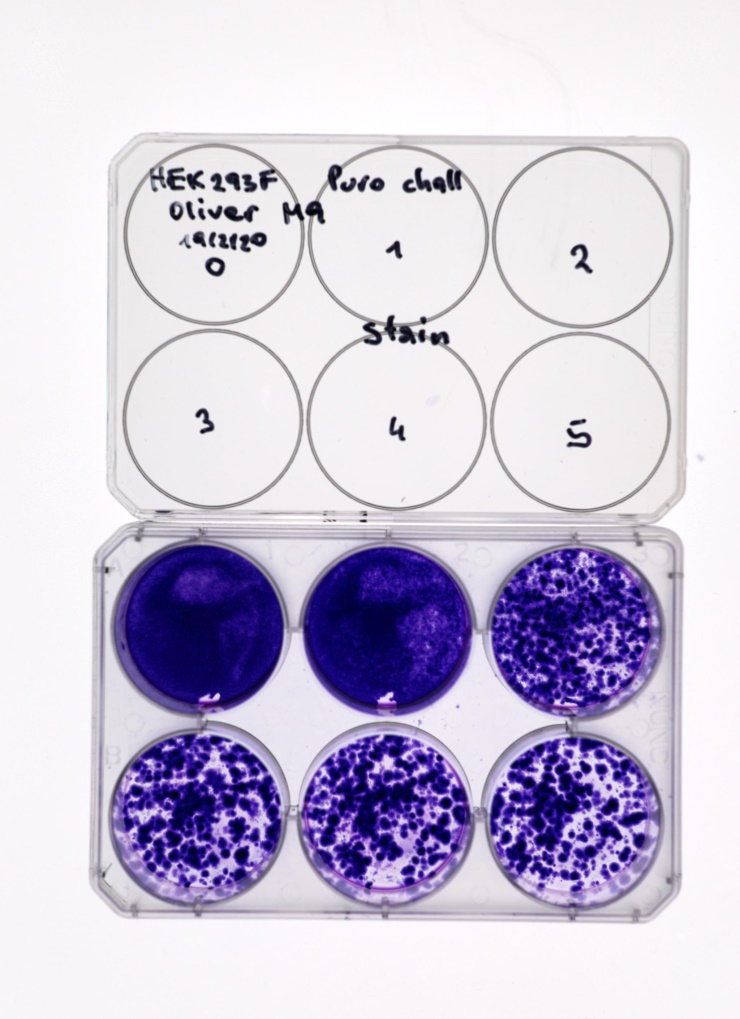 | 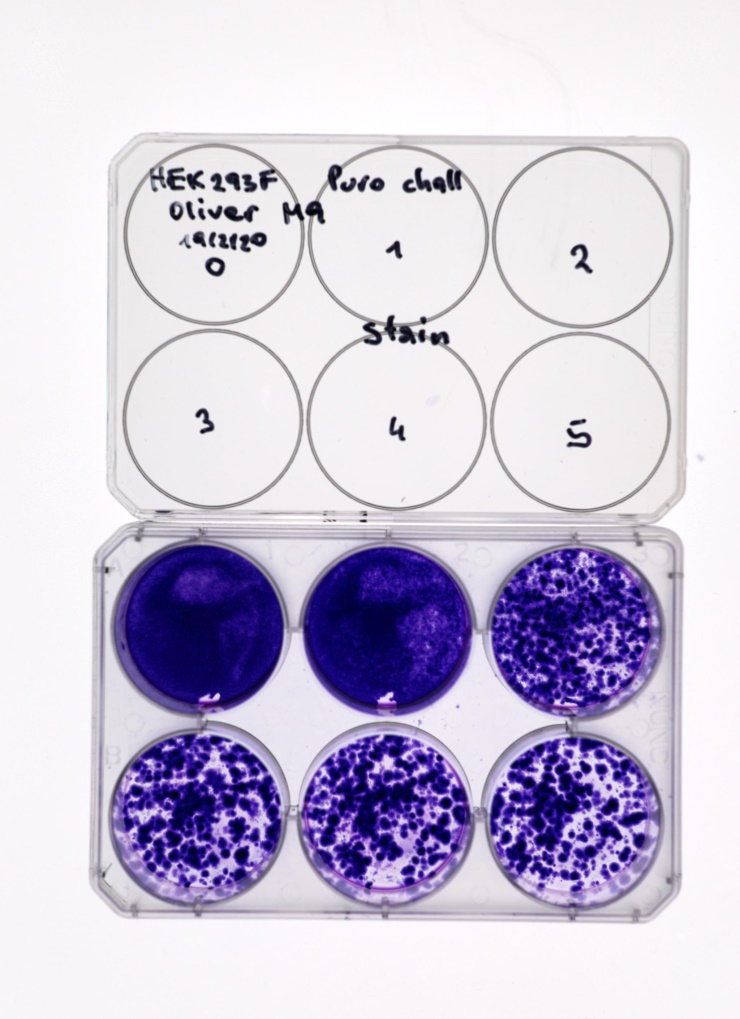 | 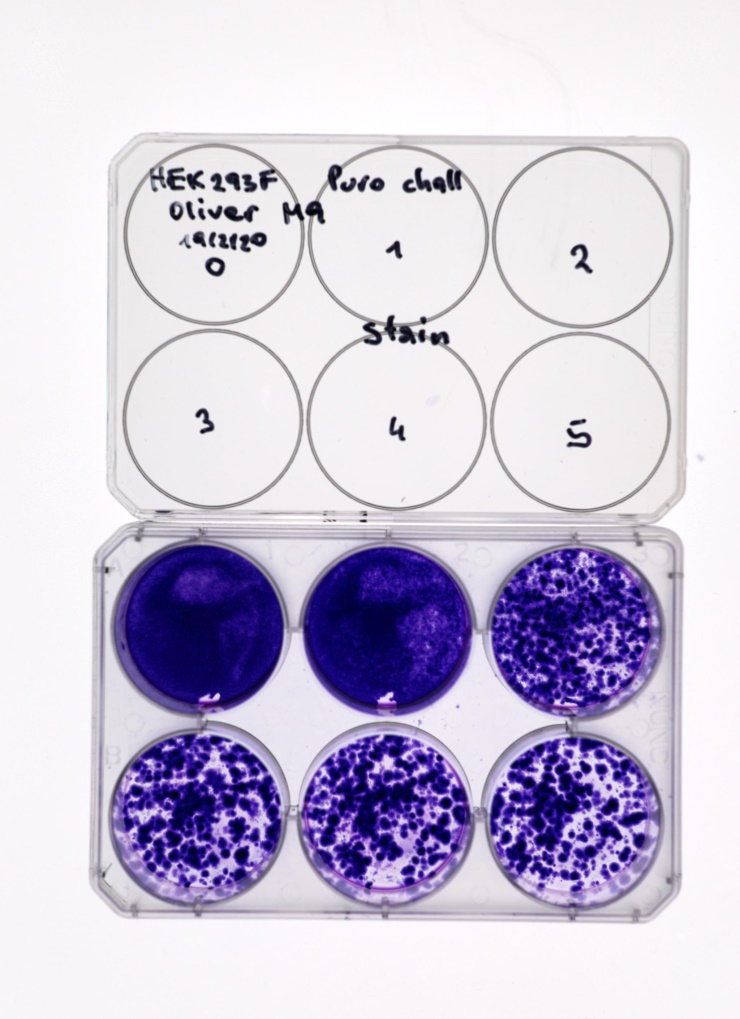 | 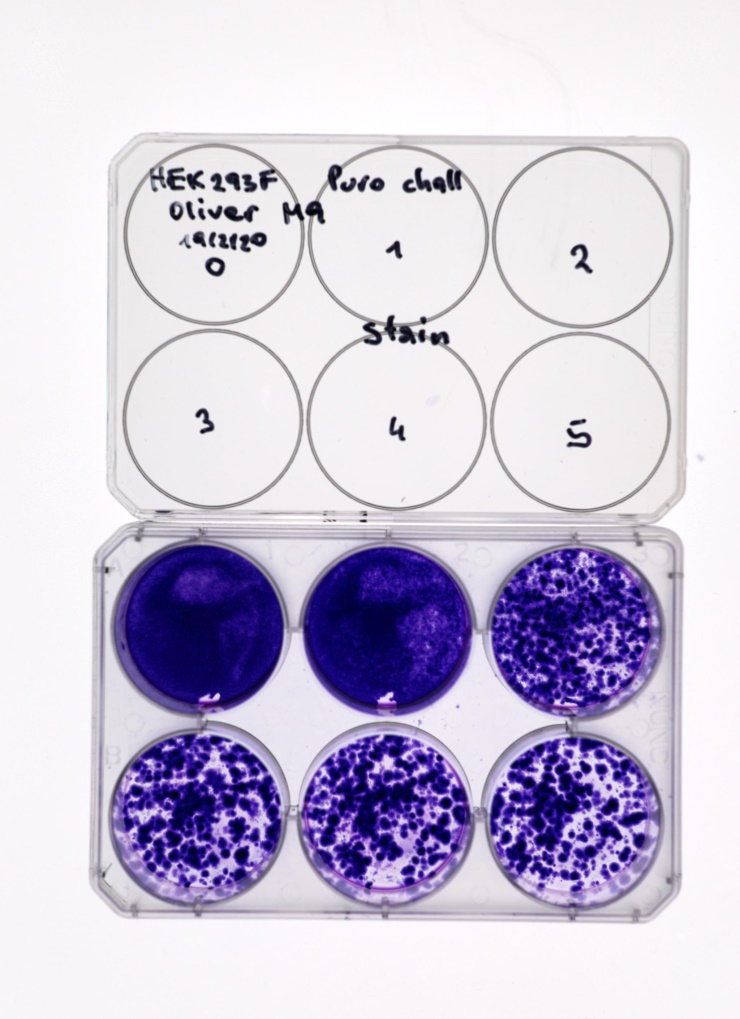 | 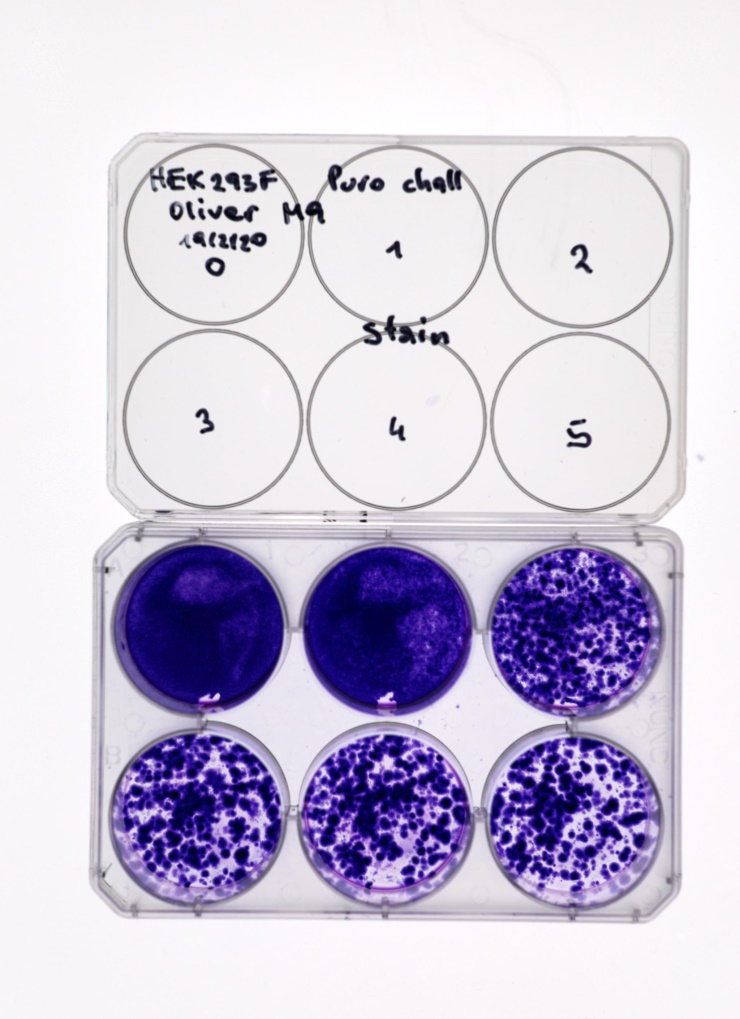 |
| E152Q | 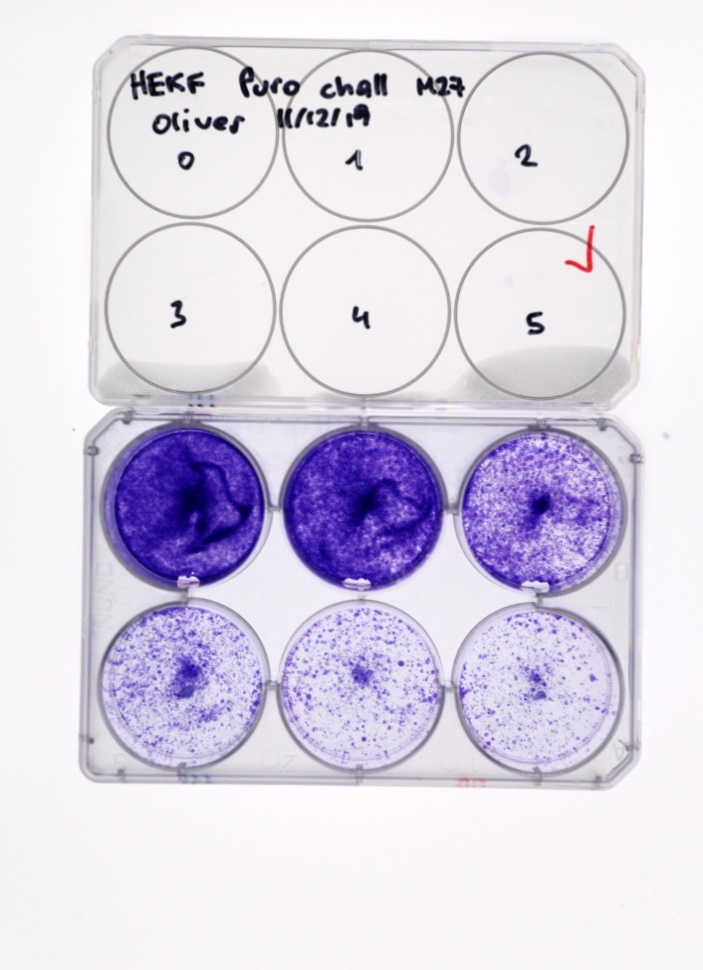 | 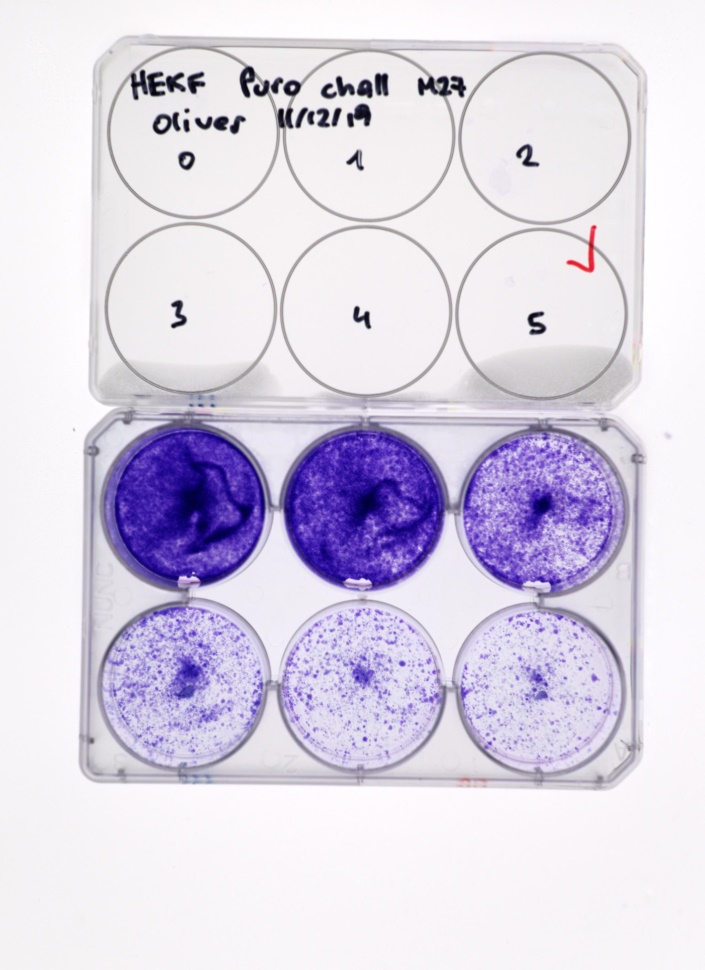 | 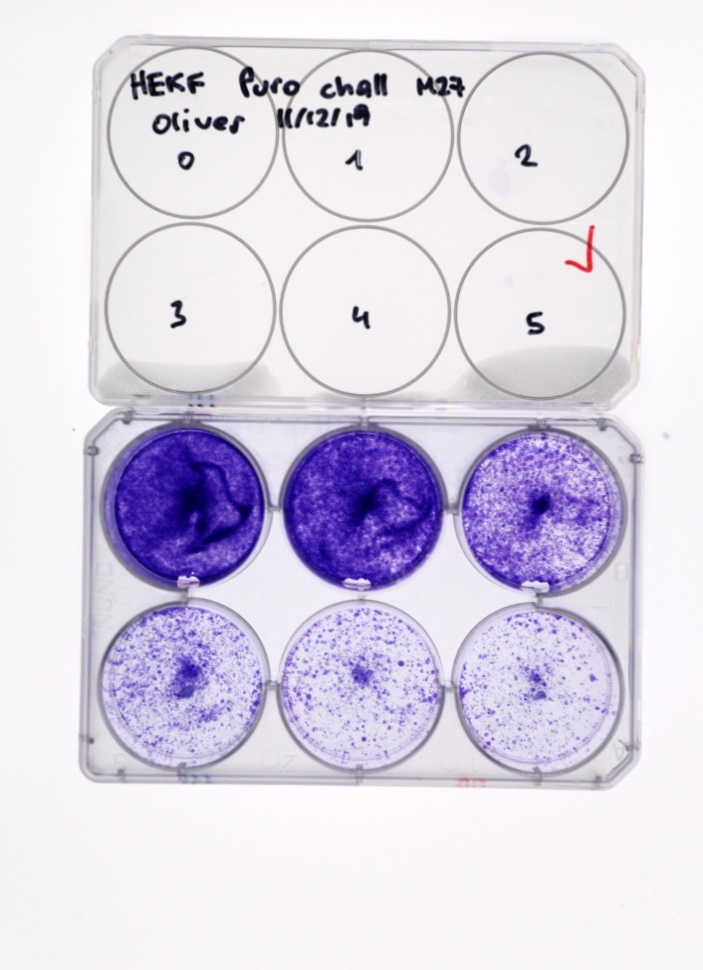 | 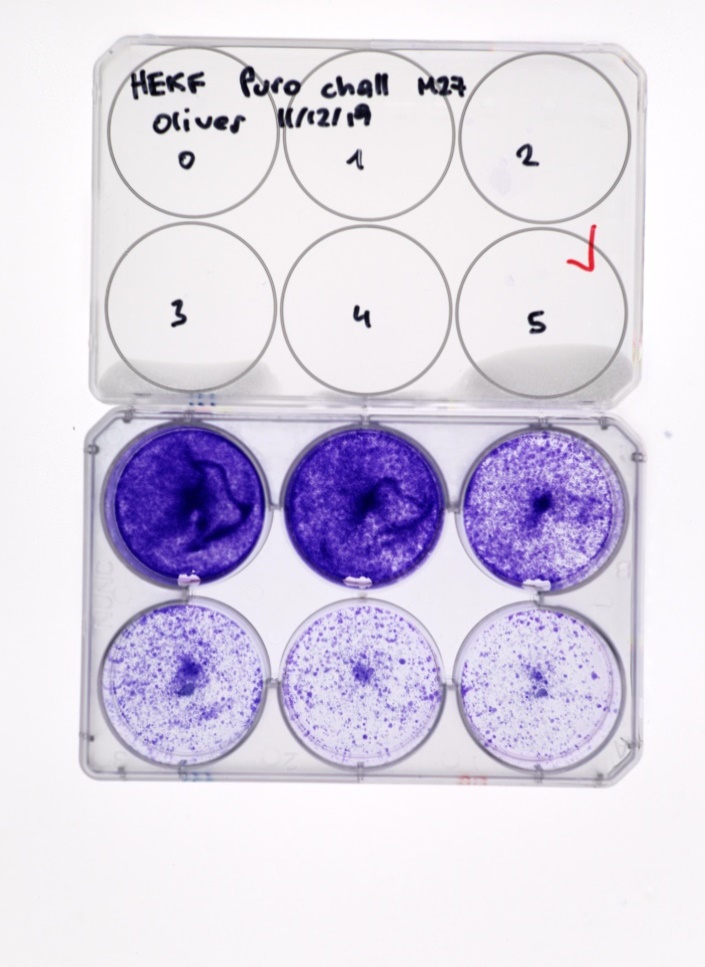 | 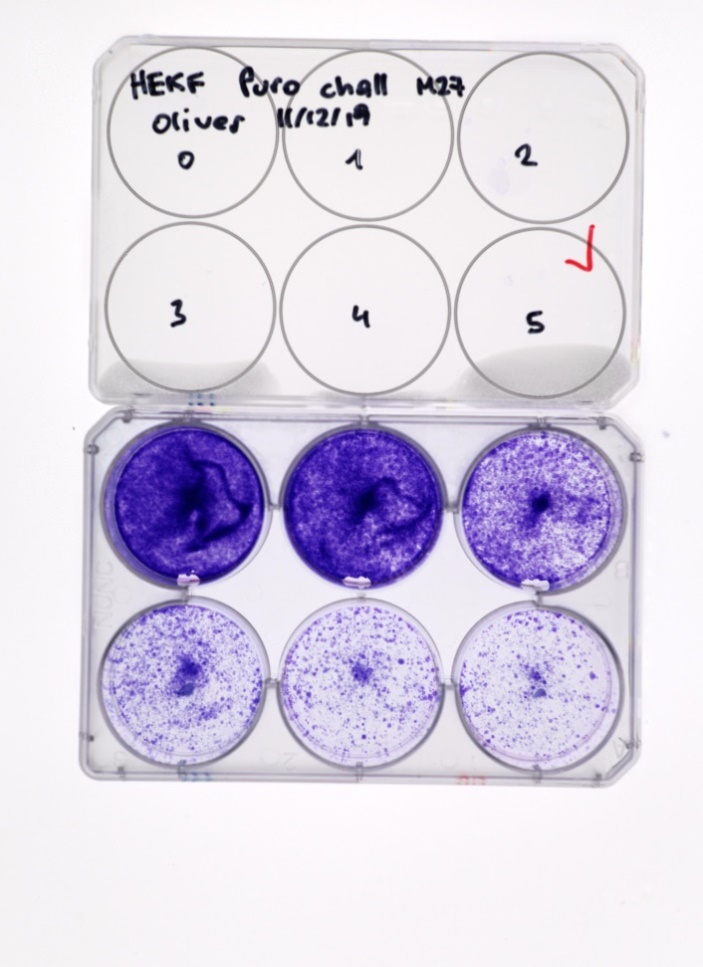 | 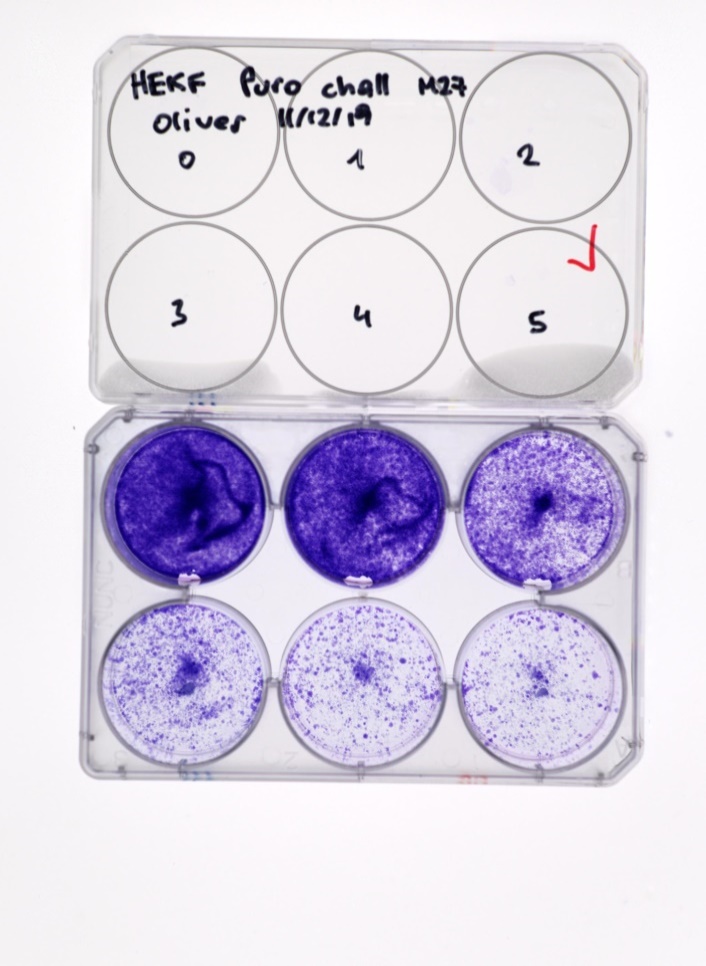 |
| A142D | 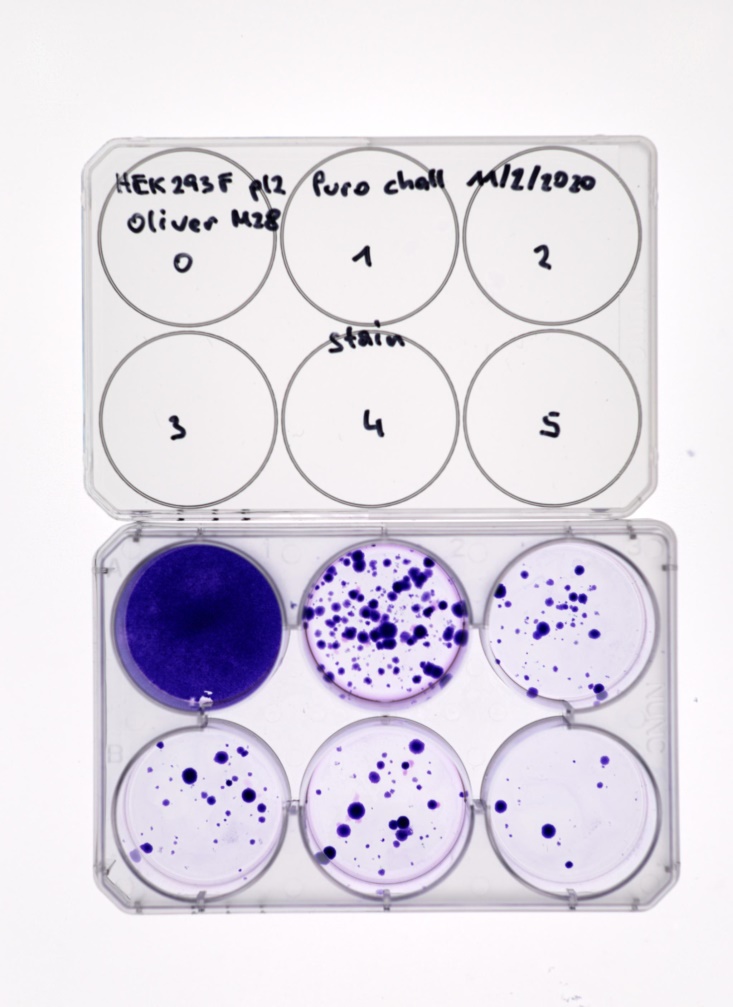 | 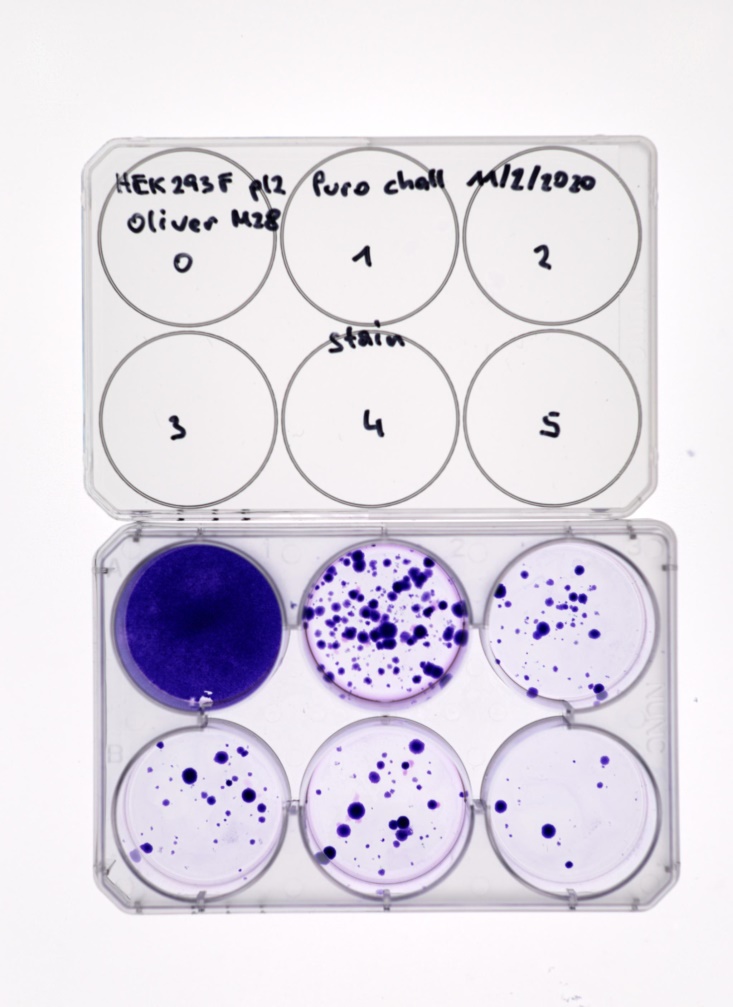 | 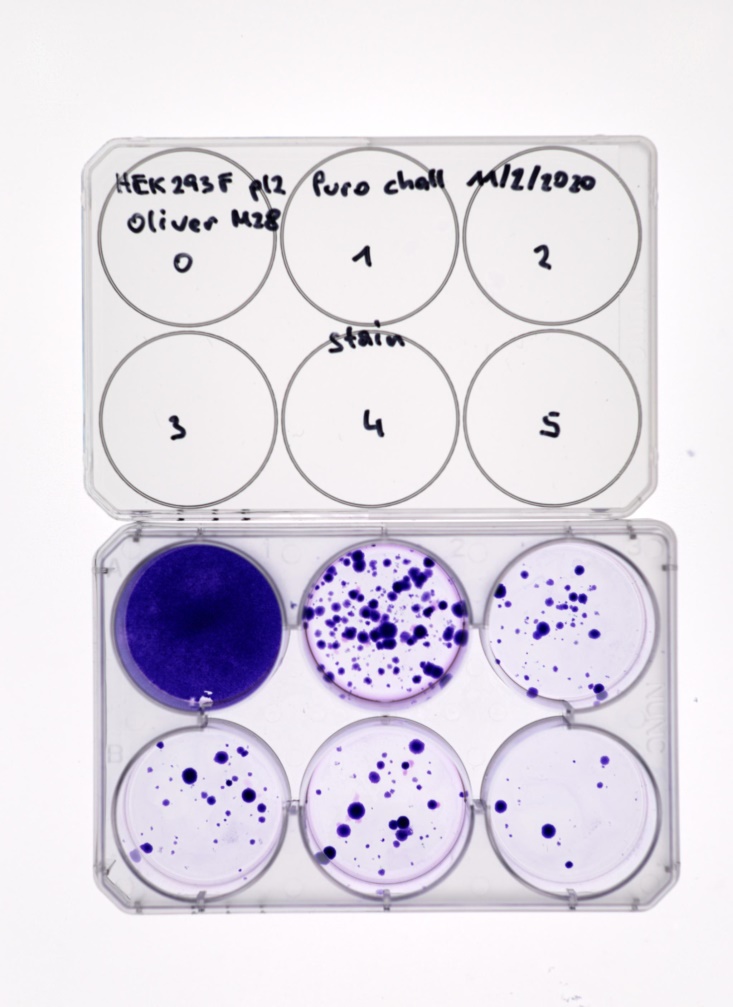 | 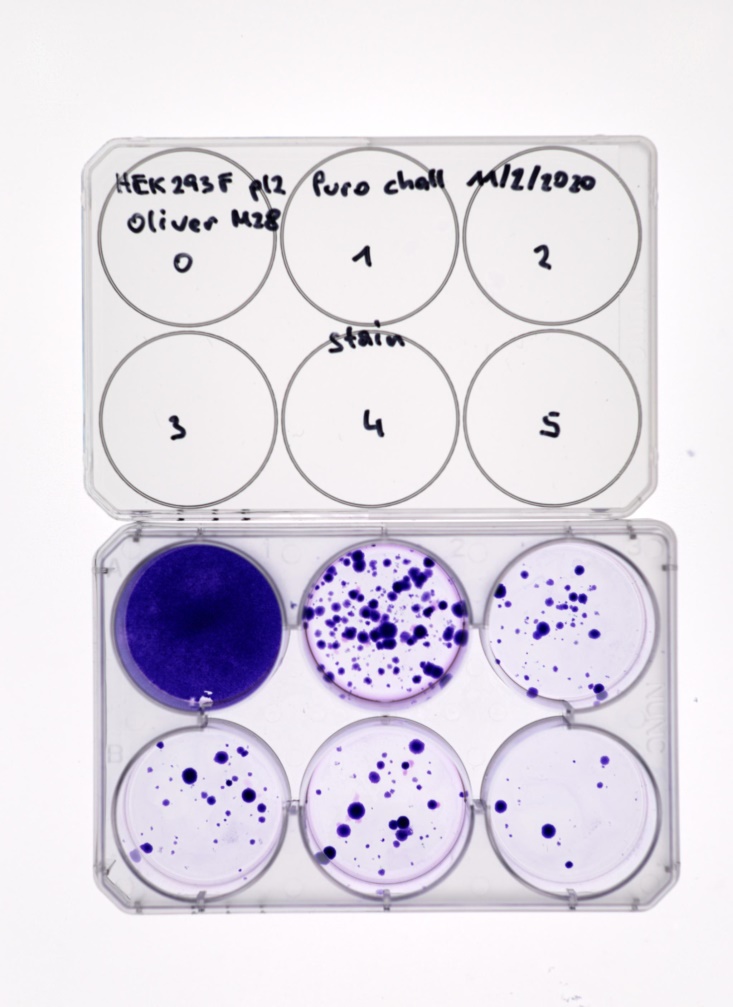 | 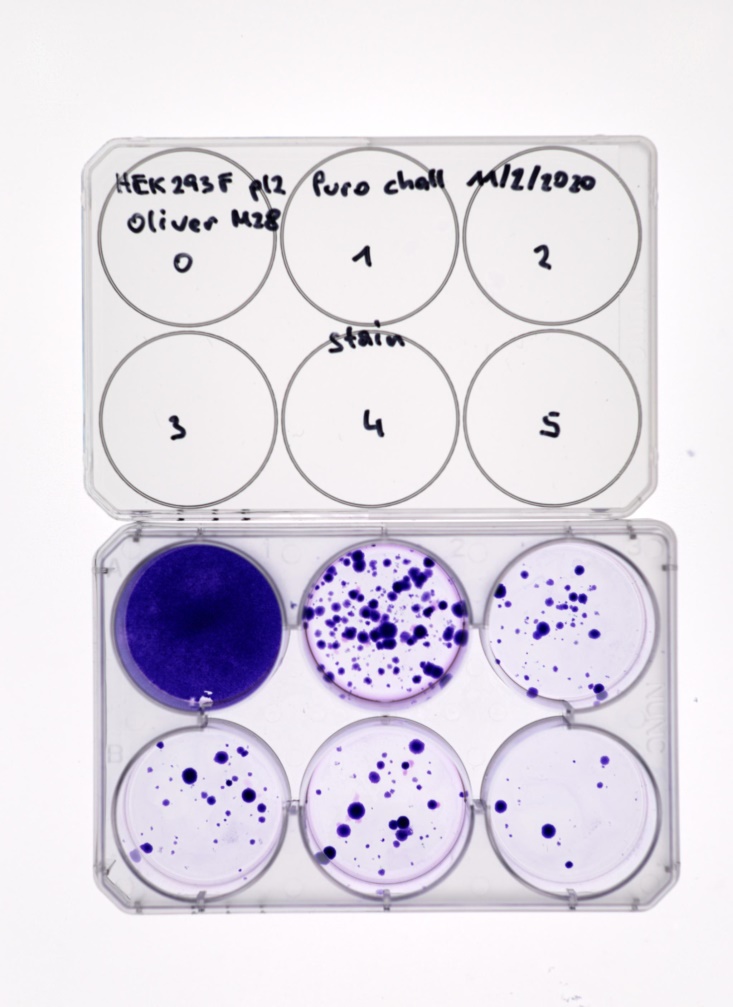 | 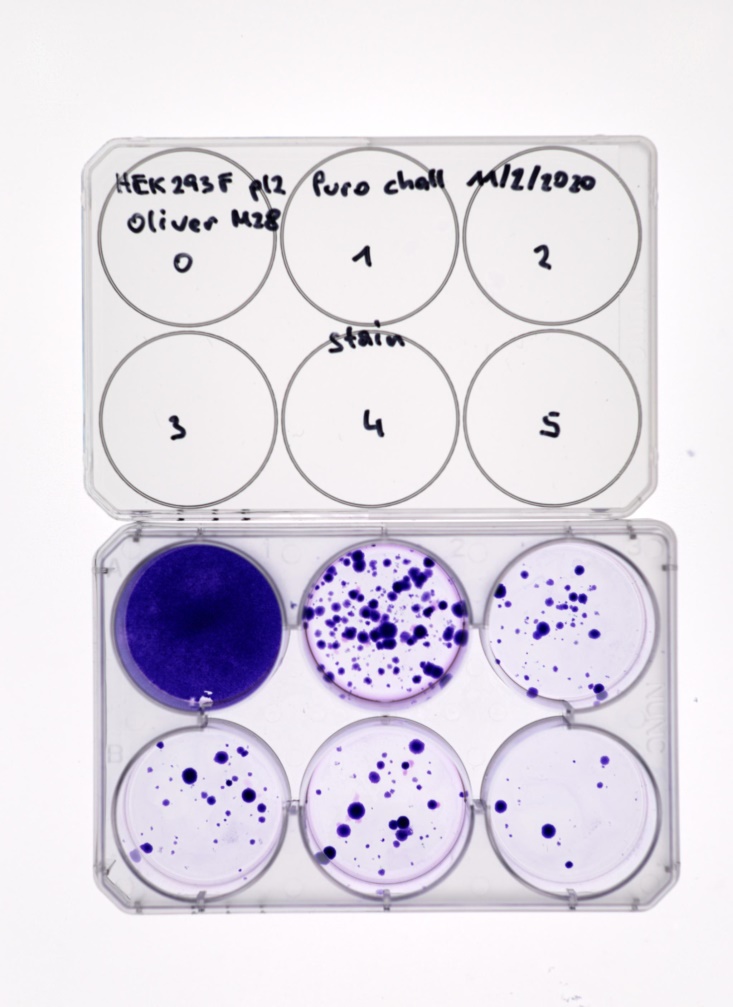 |
| L145D | 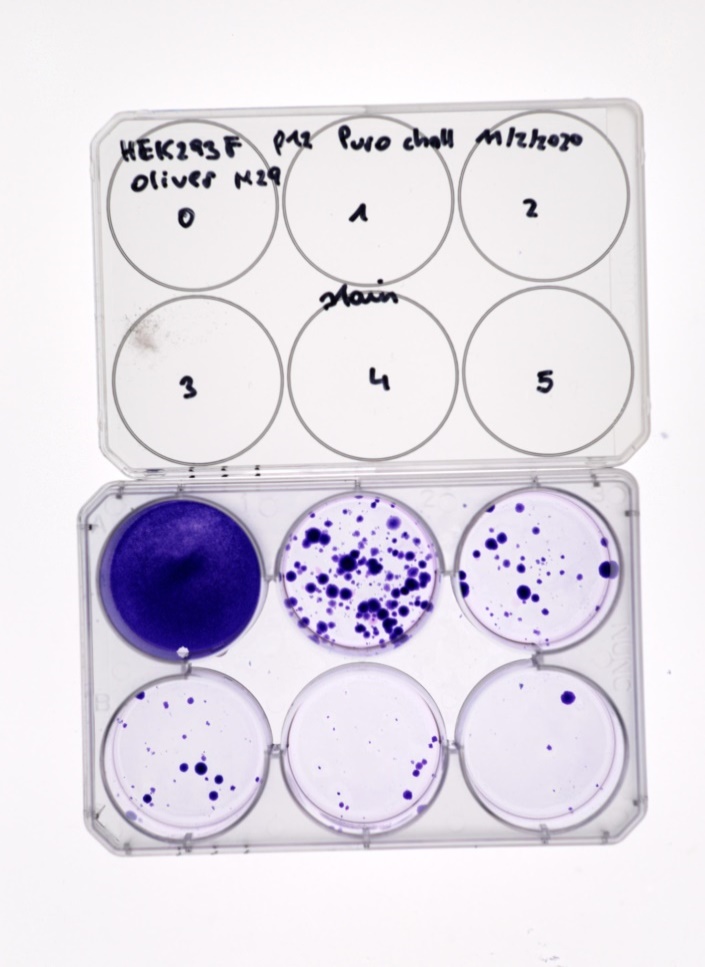 | 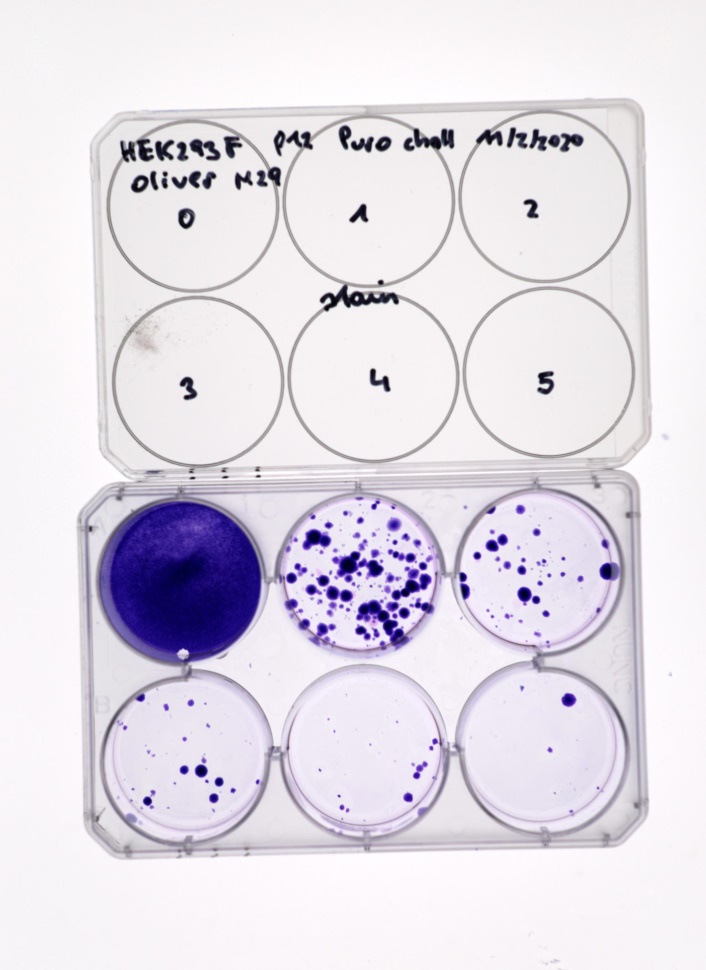 | 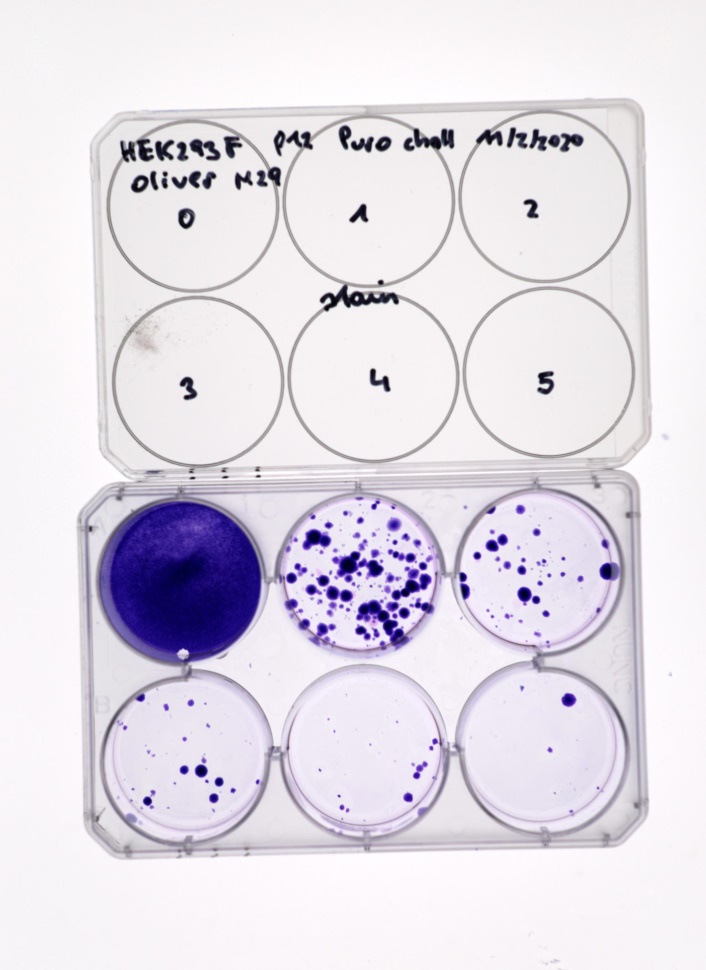 | 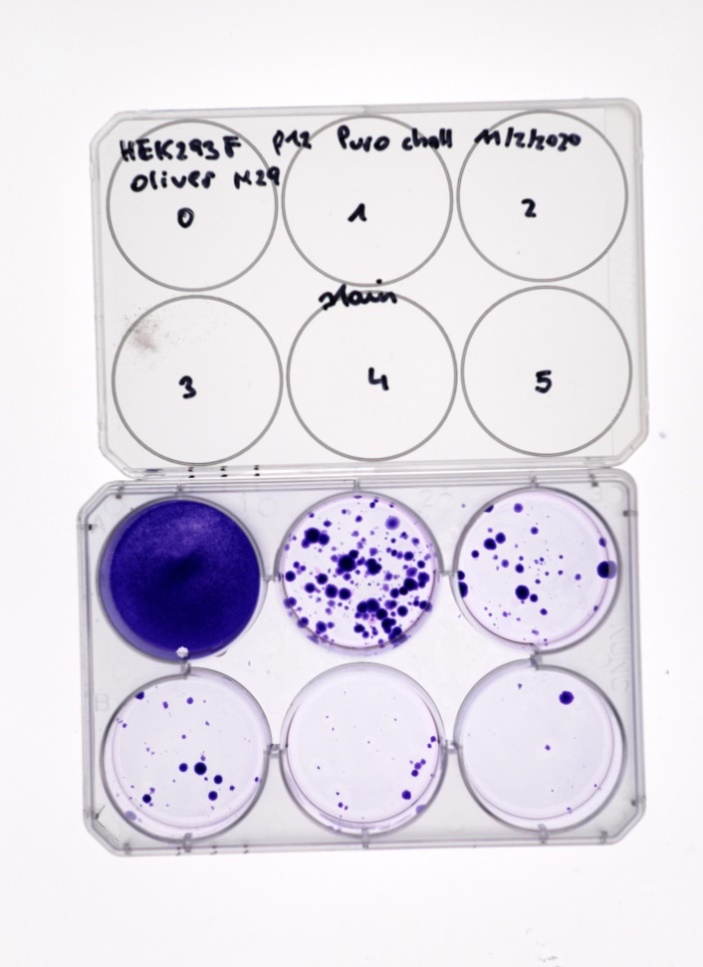 | 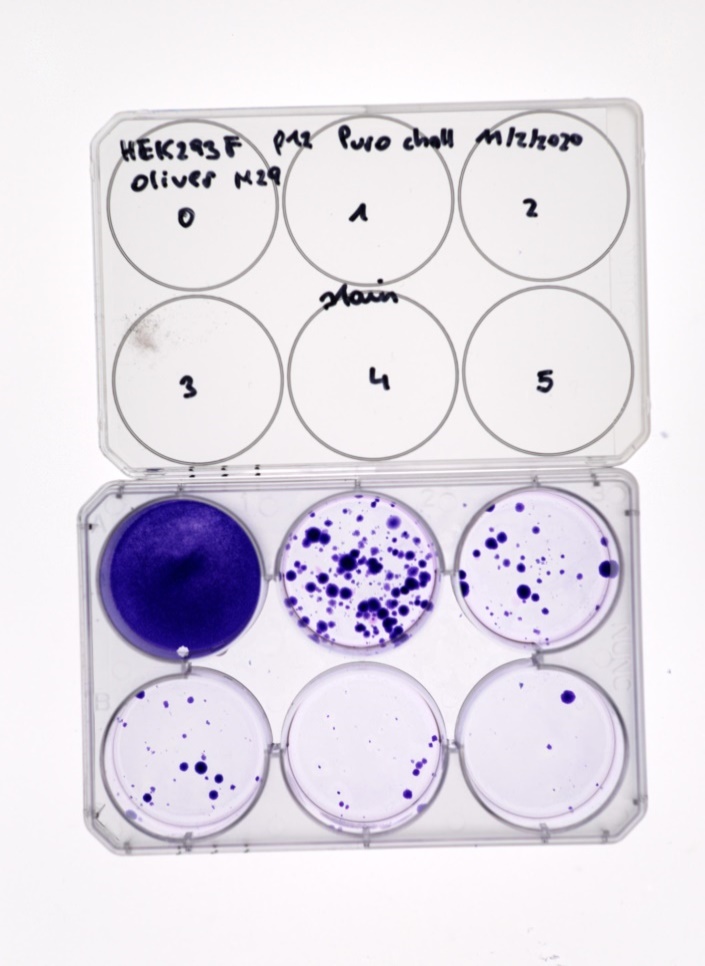 | 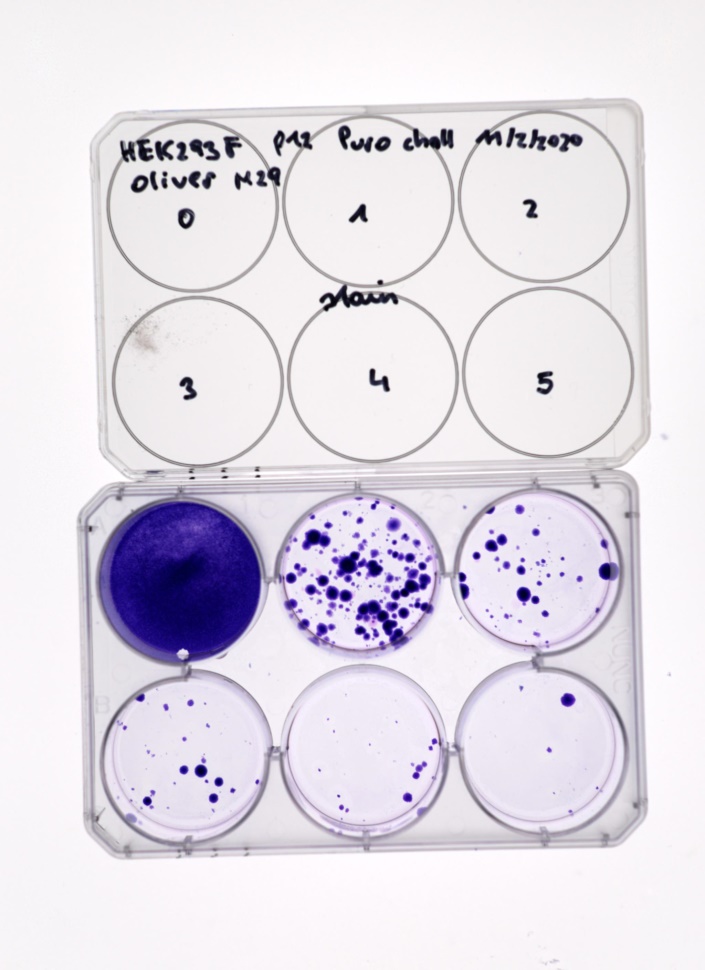 |

Supplementary Table S5: Oligonucleotide primers sets used in this study. Sequences in lowercase indicate regions of complementarity to destination vector in Gibson Assembly reactions.

| Primer purpose | Direction | Sequence (5’ > 3’) |
| --- | --- | --- |
| *pac* subcloning into pET43 | Forward | CGACGGGATCCACCGAGTACAAGCCCACGGTGCGC |
|  | Reverse | CGACGGCTAGCACCGGGCTTGCGGGTCATCCACCA |
| *pac* mutant amplification for Gibson Assembly | Forward | atttcgaggtcgaagcttccATGGGATCCACCGAGTACAAAC |
|  | Reverse | tcggatatcagtttttgctcGCTAGCACCCGGCTTACG |
| pME18s amplification for Gibson Assembly | Forward | GAGCAAAAACTGATATCCGAAG |
|  | Reverse | GGAAGCTTCGACCTCGAAATTC |
| *pac* RT-PCR | Forward | ATGGGATCCACCGAGTACAAAC |
|  | Reverse | CCTCTTCGGATATCAGTTTTTGCTC |
| *pac:erbB2* RT-PCR | Forward | N/A |
|  | Reverse | GAGGCTTGGAAGCTGCAG |
| GAPDH RT-PCR | Forward | TGAAGGTCGGAGTCAACGGATTTGG |
|  | Reverse | ATGTGGGCCATGAGGTCCACCAC |

Supplementary Table S6: Oligonucleotide primers sets used for mutagenesis in this study and the annealing temperatures (T_a_) used. The base changes indicated in lowercase.

| Mutation | Primer Name | Sequence (5’ > 3’) | T_a_ (^o^C) |
| --- | --- | --- | --- |
| Y171F | F_Pac_Mut1_Y171-F | CCTGCCGTTCtttGAACGTCTGGGTTTTACCG | 71 |
|  | R_Pac_Mut1_Y171-F | TTACGCGGCGCGCTGGTT |  |
| Y171H | F_PAC_18_Y171H | CCTGCCGTTCcACGAACGTCTGGG | 72 |
|  | R_PAC_18_Y171H | TTACGCGGCGCGCTGGTT |  |
| Y30F | F_Pac_Mut2_Y30-F | GTTTGCGGACtttCCGGCGACCCGTC | 72 |
|  | R_Pac_Mut2_Y30-F | GCCGCCGCCAGGGTACGC |  |
| T162V | F_Pac_Mut3_T162-V | GTTCCTGGAAgtgAGCGCGCCGCGTAAC | 71 |
|  | R_Pac_Mut3_T162-V | GCCGGAACACCCGCACGT |  |
| T162A | F_Pac_Mut4_T162-A | GTTCCTGGAAgcgAGCGCGCCGC | 72 |
|  | R_Pac_Mut4_T162-A | GCCGGAACACCCGCACGT |  |
| N167D | F_Pac_Mut5_N167-D | CGCGCCGCGTgatCTGCCGTTCT | 72 |
|  | R_Pac_Mut5_N167-D | CTGGTTTCCAGGAACGCCGG |  |
| N167Q | F_Pac_Mut6_N167-Q | CGCGCCGCGTcagCTGCCGTTCT | 72 |
|  | R_Pac_Mut6_N167-Q | CTGGTTTCCAGGAACGCCGG |  |
| N167A | F_Pac_Mut10_N167-A | CGCGCCGCGTgcgCTGCCGTTCT | 67 |
|  | R_Pac_Mut10_N167-A | CTGGTTTCCAGGAACGCC |  |
| A26S | F_Pac_Mut7_A26-S | CCTGGCGGCGagcTTTGCGGACTATCCGGC | 72 |
|  | R_Pac_Mut7_A26-S | GTACGCACCGCACGCGGA |  |
| T77S | F_Pac_Mut9_T77-S | GGCGGTTTGGagcACCCCGGAGA | 72 |
|  | R_Pac_Mut9_T77-S | ACCGCCGCACCATCATCCG |  |
| E152Q | F_PAC_27_E152Q | GGAGGCGGCGcagCGTGCGGGTG | 72 |
|  | R_PAC_27_E152Q | ACGCCCGGCAGAACCACC |  |
| A142D | F_PAC_28_A142D | CTGGGTAGCGatGTGGTTCTGC | 64 |
|  | R_PAC_28_A142D | GCCTTTACCCTGGTGGTC |  |
| L145D | F_PAC_29_L145D | CGCGGTGGTTgatCCGGGCGTGG | 67 |
|  | R_PAC_29_L145D | CTACCCAGGCCTTTACCC |  |

References

1. Krissinel, E. & Henrick, K. Computational Life Sciences, First International Symposium, CompLife 2005, Konstanz, Germany, September 25-27, 2005. Proceedings. 67–78 (2005) doi:10.1007/11560500_7.

2. Krissinel, E. & Henrick, K. Secondary-structure matching (SSM), a new tool for fast protein structure alignment in three dimensions. *Acta Crystallogr Sect D Biological Crystallogr* **60**, 2256–2268 (2004).

3. Krissinel, E. B. *et al.* The new CCP4 Coordinate Library as a toolkit for the design of coordinate-related applications in protein crystallography. *Acta Crystallogr Sect D Biological Crystallogr* **60**, 2250–2255 (2004).
